# Supplementary material for: Learning from pandemics: Feature selection and clustering for robust digital disease nowcasting
Source: PLoS One. 2026 Oct 1;21(10):e0358063. doi: 10.1371/journal.pone.0358063 (PMC13630223; doi:10.1371/journal.pone.0358063)
Supplement: Supplementary file 1 [file pone.0358063.s001.pdf]

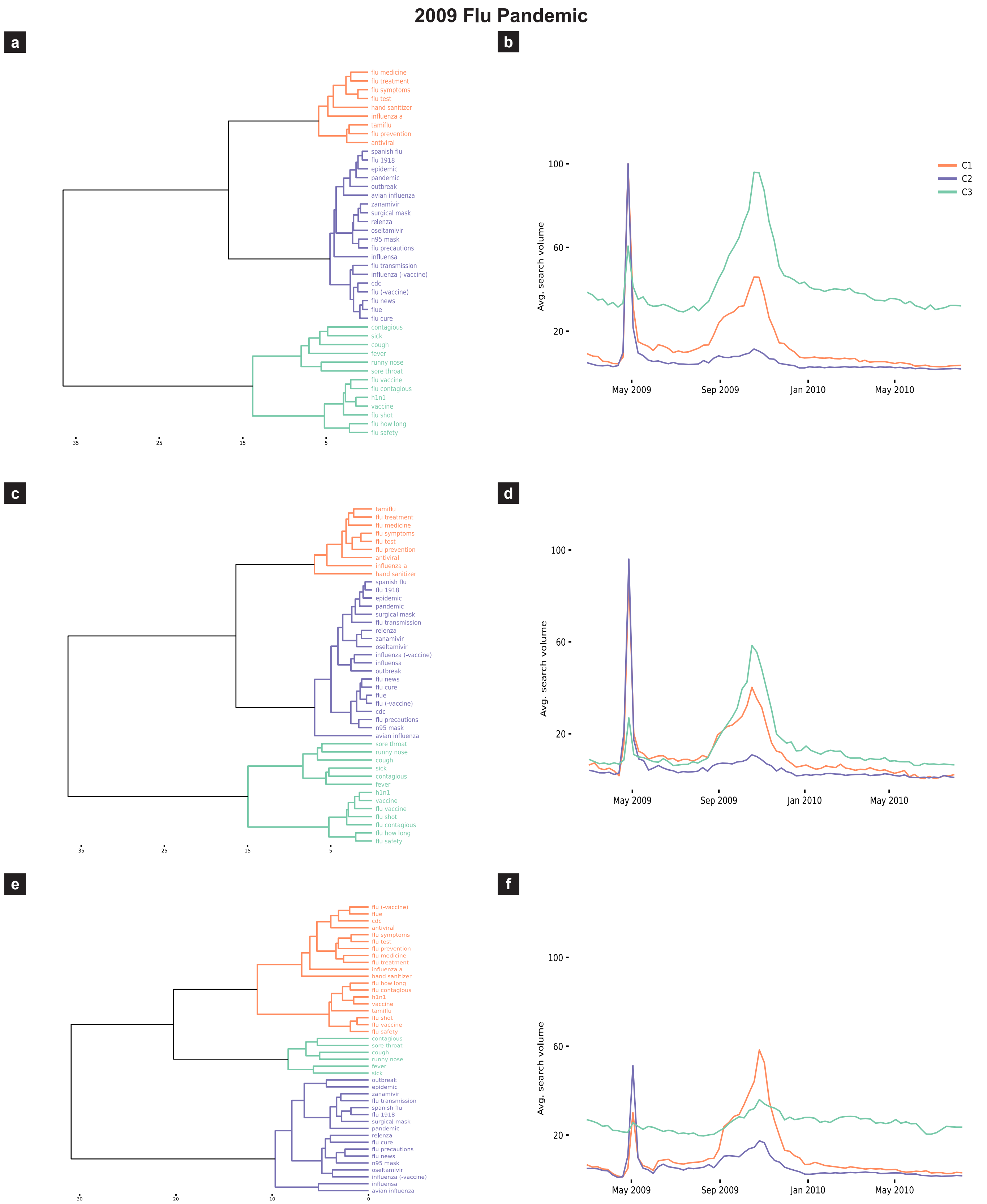

S1 Fig. Stability of clustering and centroid patterns across independent Google Trends data extractions. Panels a) and b) correspond to the first data extraction from Google Trends, with a) showing the hierarchical clustering dendrogram and b) the resulting cluster centroids. Panels c) and d) correspond to a second independent extraction, and panels e) and f) to a third extraction, each performed at different times, and different years. Despite some search terms shifting between clusters across extractions, likely due to Google Trends' resampling, the temporal patterns of the centroids remain highly consistent. This preservation of the overall signal supports that the clustering captures real behavioral dynamics rather than artifacts of the data retrieval process.
