# Supplementary material for: Learning from pandemics: Feature selection and clustering for robust digital disease nowcasting
Source: PLoS One. 2026 Oct 1;21(10):e0358063. doi: 10.1371/journal.pone.0358063 (PMC13630223; doi:10.1371/journal.pone.0358063)
Supplement: Supplementary file 3 [file pone.0358063.s003.pdf]

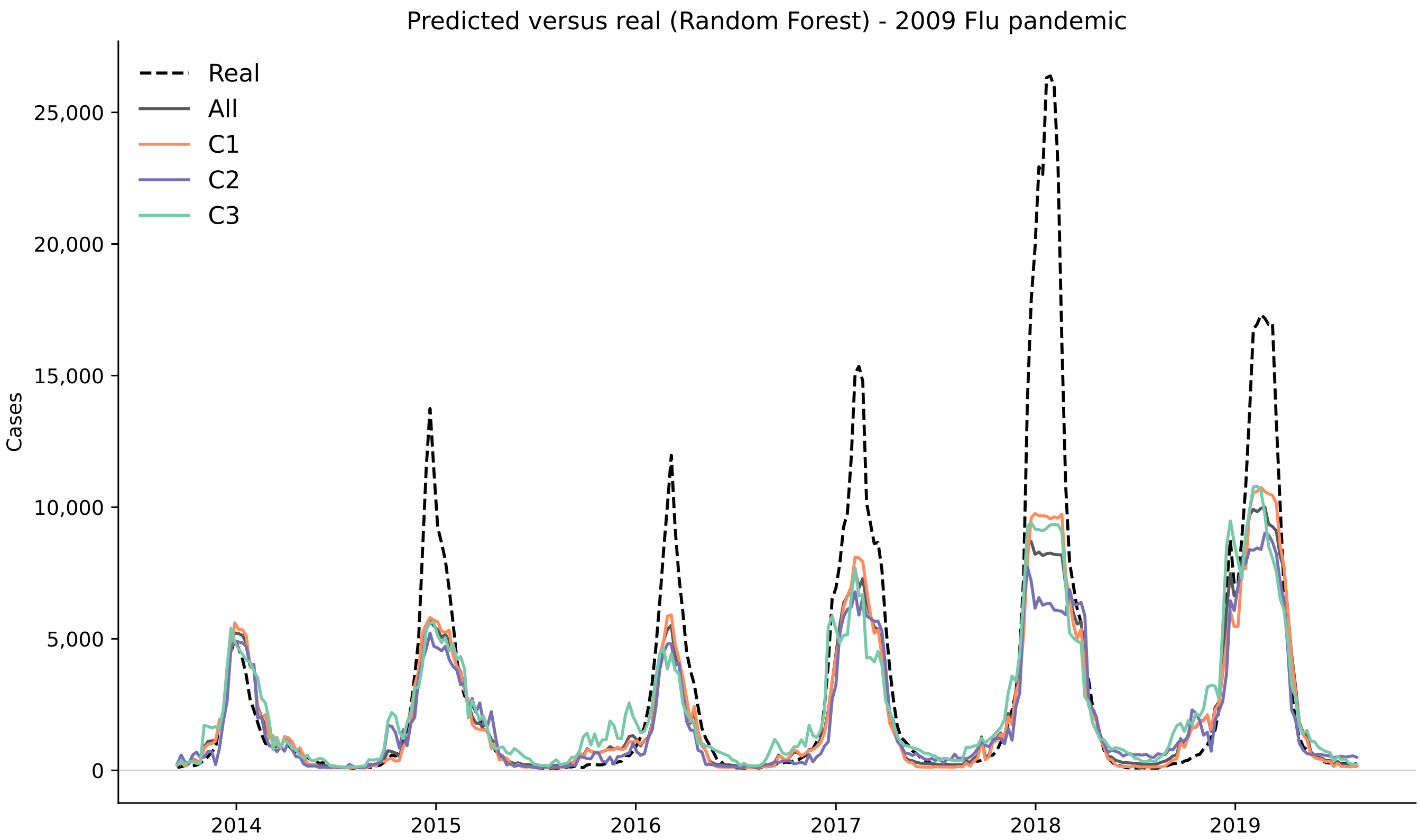

S3 Fig. Predicted and observed cases for the 2009 Flu pandemic using Random Forest model.
