# Supplementary material for: Learning from pandemics: Feature selection and clustering for robust digital disease nowcasting
Source: PLoS One. 2026 Oct 1;21(10):e0358063. doi: 10.1371/journal.pone.0358063 (PMC13630223; doi:10.1371/journal.pone.0358063)
Supplement: Supplementary file 4 [file pone.0358063.s004.pdf]

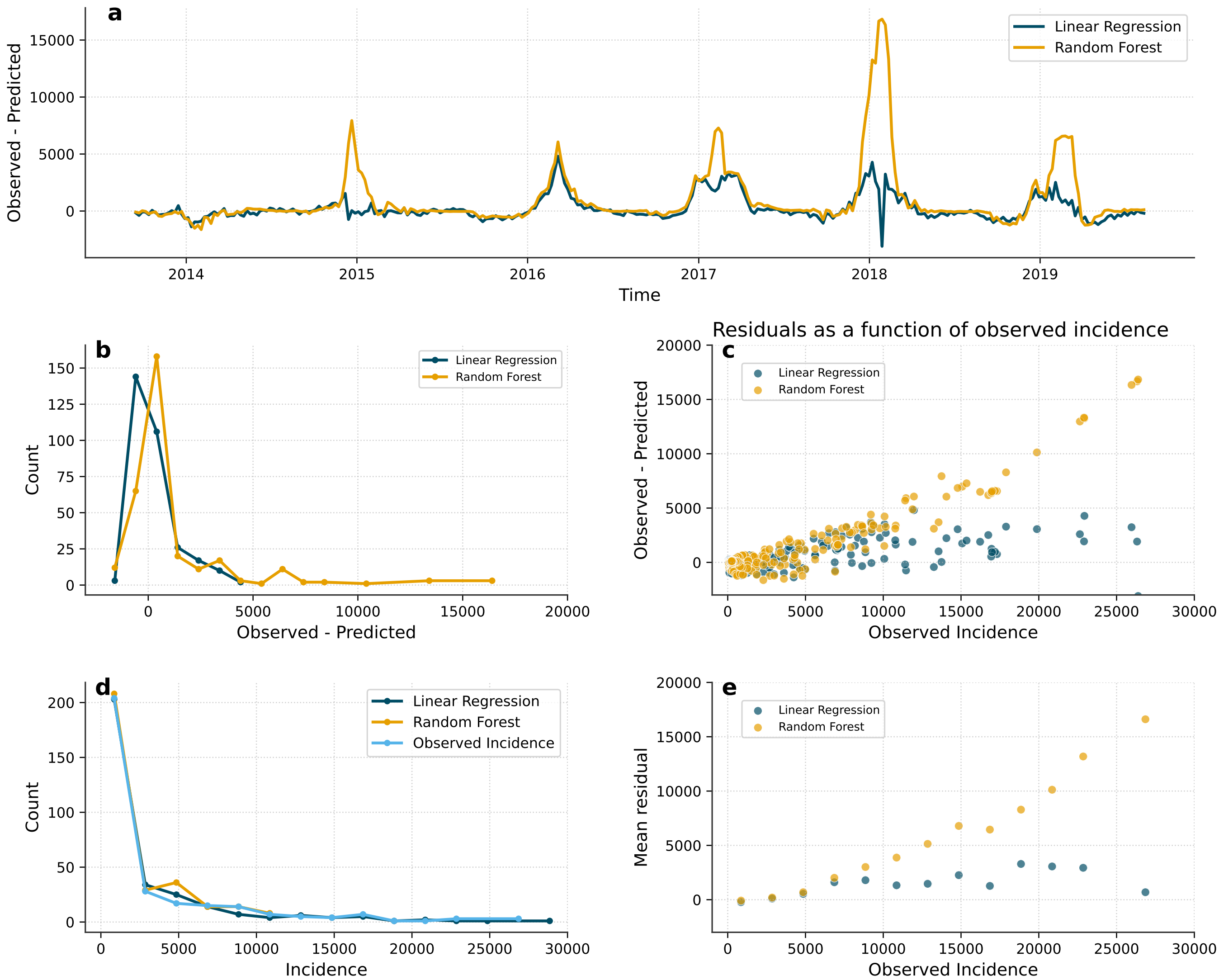

S4 Fig. Residual distribution for the flu predictions. Panel a) shows the temporal variation in residuals. We observe that both models have difficulty predicting the height of the flu outbreaks, and that the effect is more pronounced for the random forest. Panel b) shows the distribution of pooled residuals where we see that the linear regression has less extreme values than the random forest. Panels c) and e) show the correlation between residuals and the observed incidence (panel c) shows the raw data while e) shows the mean residual for bins of incidence). When incidence is low, the two models are equivalent, however, for high values of incidence, the random forest residuals are much higher. Panel d) shows the distribution of incidence using the same bins as e).
