# Supplementary material for: Learning from pandemics: Feature selection and clustering for robust digital disease nowcasting
Source: PLoS One. 2026 Oct 1;21(10):e0358063. doi: 10.1371/journal.pone.0358063 (PMC13630223; doi:10.1371/journal.pone.0358063)
Supplement: Supplementary file 6 [file pone.0358063.s006.pdf]

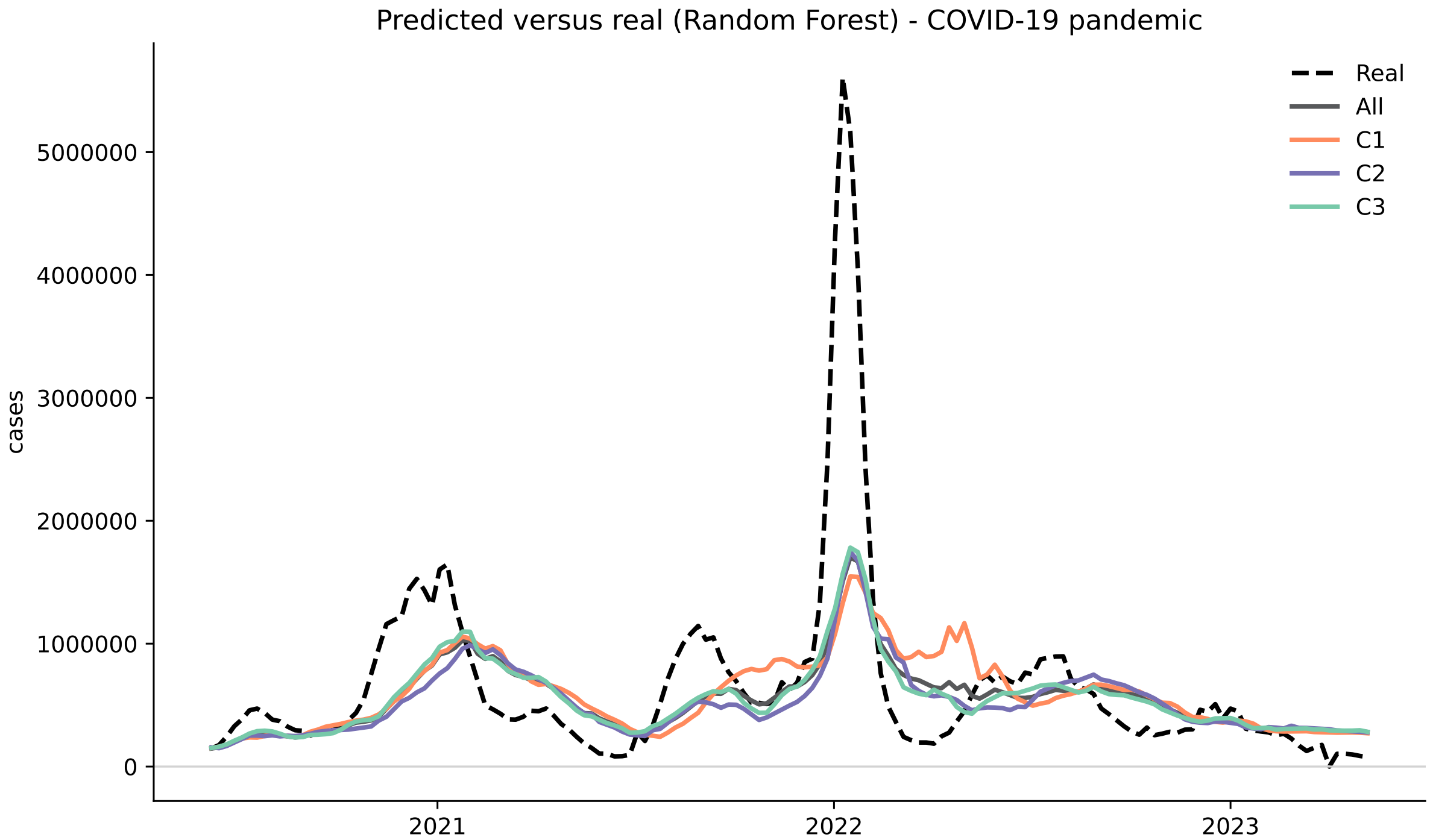

S6 Fig. Predicted and observed cases for COVID-19 pandemic using Random Forest model.
