# Supplementary material for: Learning from pandemics: Feature selection and clustering for robust digital disease nowcasting
Source: PLoS One. 2026 Oct 1;21(10):e0358063. doi: 10.1371/journal.pone.0358063 (PMC13630223; doi:10.1371/journal.pone.0358063)
Supplement: Supplementary file 7 [file pone.0358063.s007.pdf]

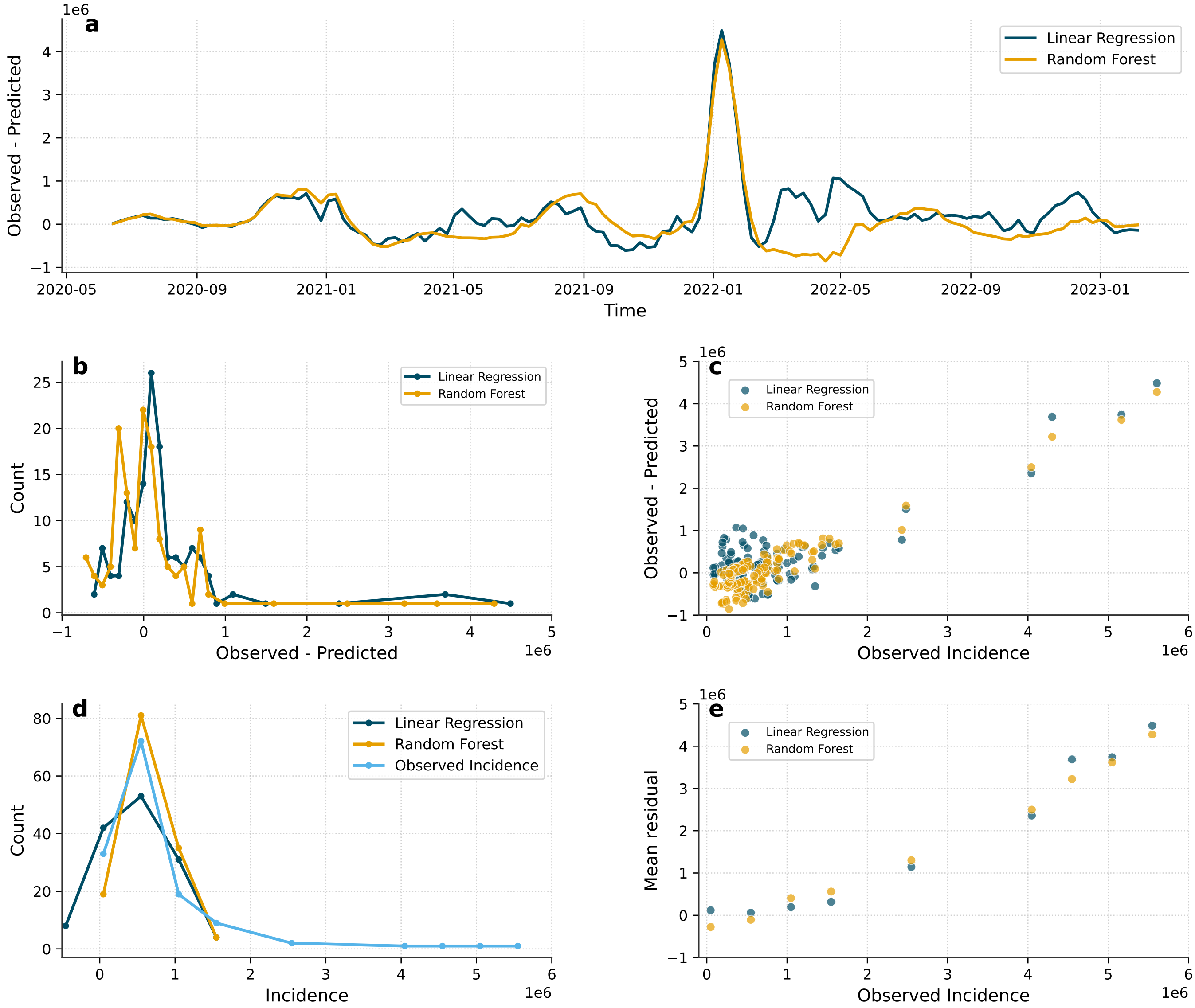

S7 Fig. Residual distribution for the COVID-19 predictions. The panels are the same as in S4 Fig. Panel a) shows the temporal variation in residuals. Panel b) shows the distribution of pooled residuals. Panels c) and e) show the correlation between residuals and the observed incidence (panel c) shows the raw data while e) shows the mean residual for bins of incidence). Panel d) shows the distribution of incidence using the same bins as e). In this case, the differences in the residuals of the two models are small.
