# Supplementary material for: Learning from pandemics: Feature selection and clustering for robust digital disease nowcasting
Source: PLoS One. 2026 Oct 1;21(10):e0358063. doi: 10.1371/journal.pone.0358063 (PMC13630223; doi:10.1371/journal.pone.0358063)
Supplement: Supplementary file 8 [file pone.0358063.s008.pdf]

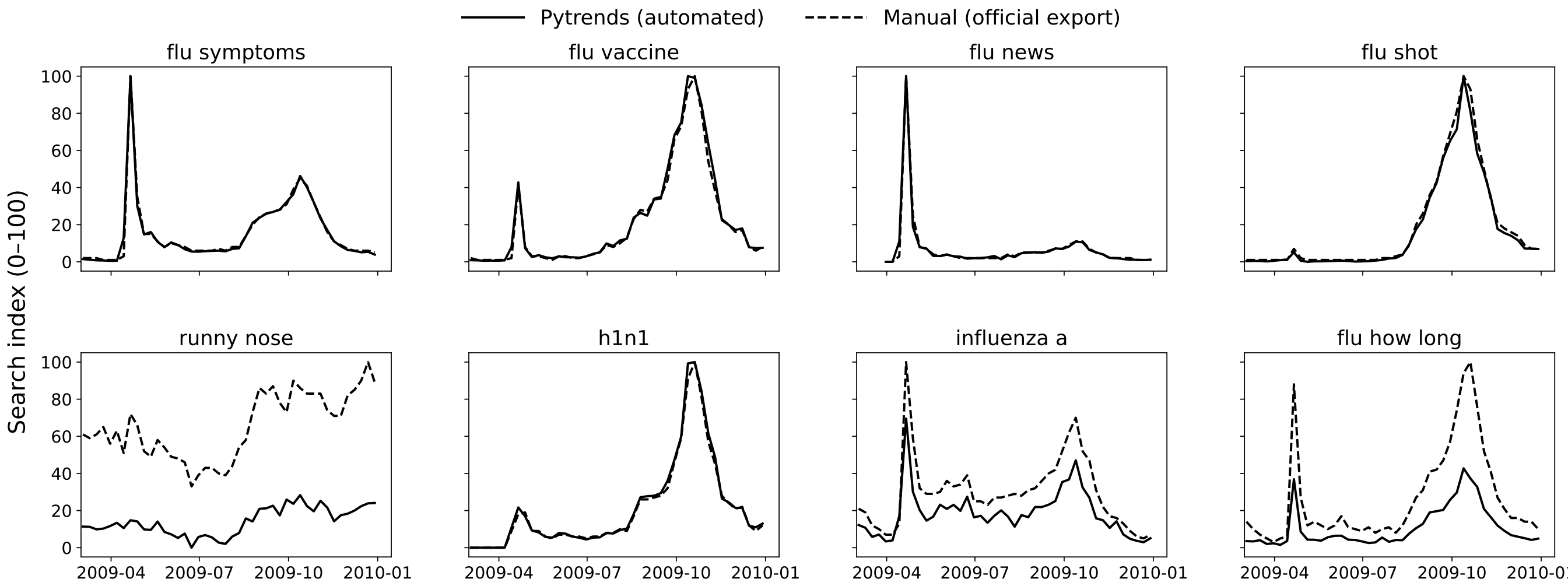

S8 Fig. Flu reproducibility: Pytrends (solid black) versus official Google Trends CSV exports (dashed black) for representative terms covering clusters C1–C3 (US, H1N1 window: 2009–03–01 to 2010–01–08). See S7 Table for paired Pearson/Spearman correlations and MAD values.
