# Supplementary material for: Learning from pandemics: Feature selection and clustering for robust digital disease nowcasting
Source: PLoS One. 2026 Oct 1;21(10):e0358063. doi: 10.1371/journal.pone.0358063 (PMC13630223; doi:10.1371/journal.pone.0358063)
Supplement: Supplementary file 9 [file pone.0358063.s009.pdf]

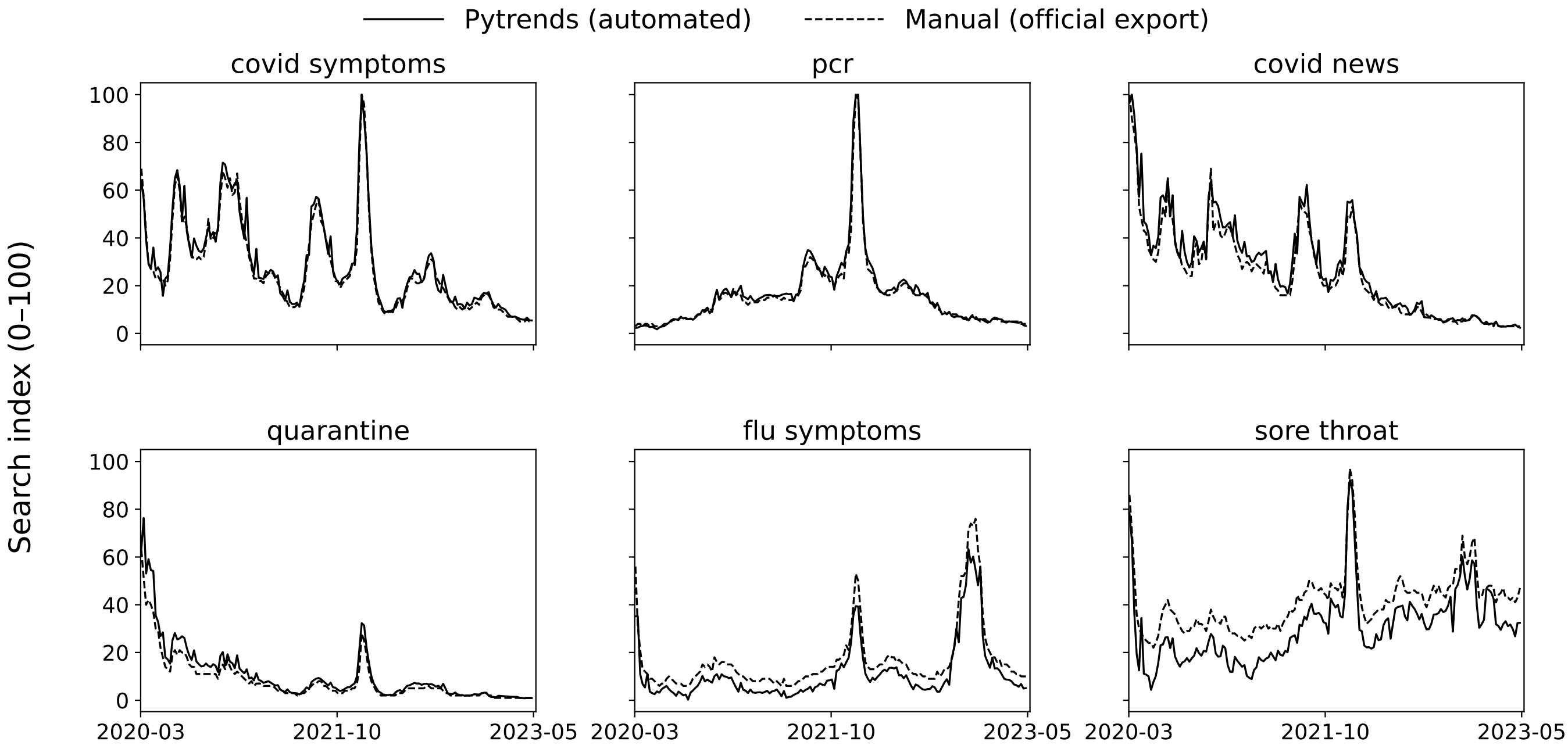

S9 Fig. COVID-19 reproducibility: Pytrends (solid black) versus official Google Trends CSV exports (dashed black) for search terms spanning clusters C1–C3 (US, pandemic period: 2020–03–15 to 2023–05–07). The visual overlap across terms indicates that *pytrends* retrievals closely reproduce the temporal patterns observed in official Google Trends data, confirming the robustness of automated queries for longitudinal analysis. See S8 Table for corresponding Pearson and Spearman correlations and MAD values.
