# Supplementary material for: Learning from pandemics: Feature selection and clustering for robust digital disease nowcasting
Source: PLoS One. 2026 Oct 1;21(10):e0358063. doi: 10.1371/journal.pone.0358063 (PMC13630223; doi:10.1371/journal.pone.0358063)
Supplement: Supplementary file 10 [file pone.0358063.s010.pdf]

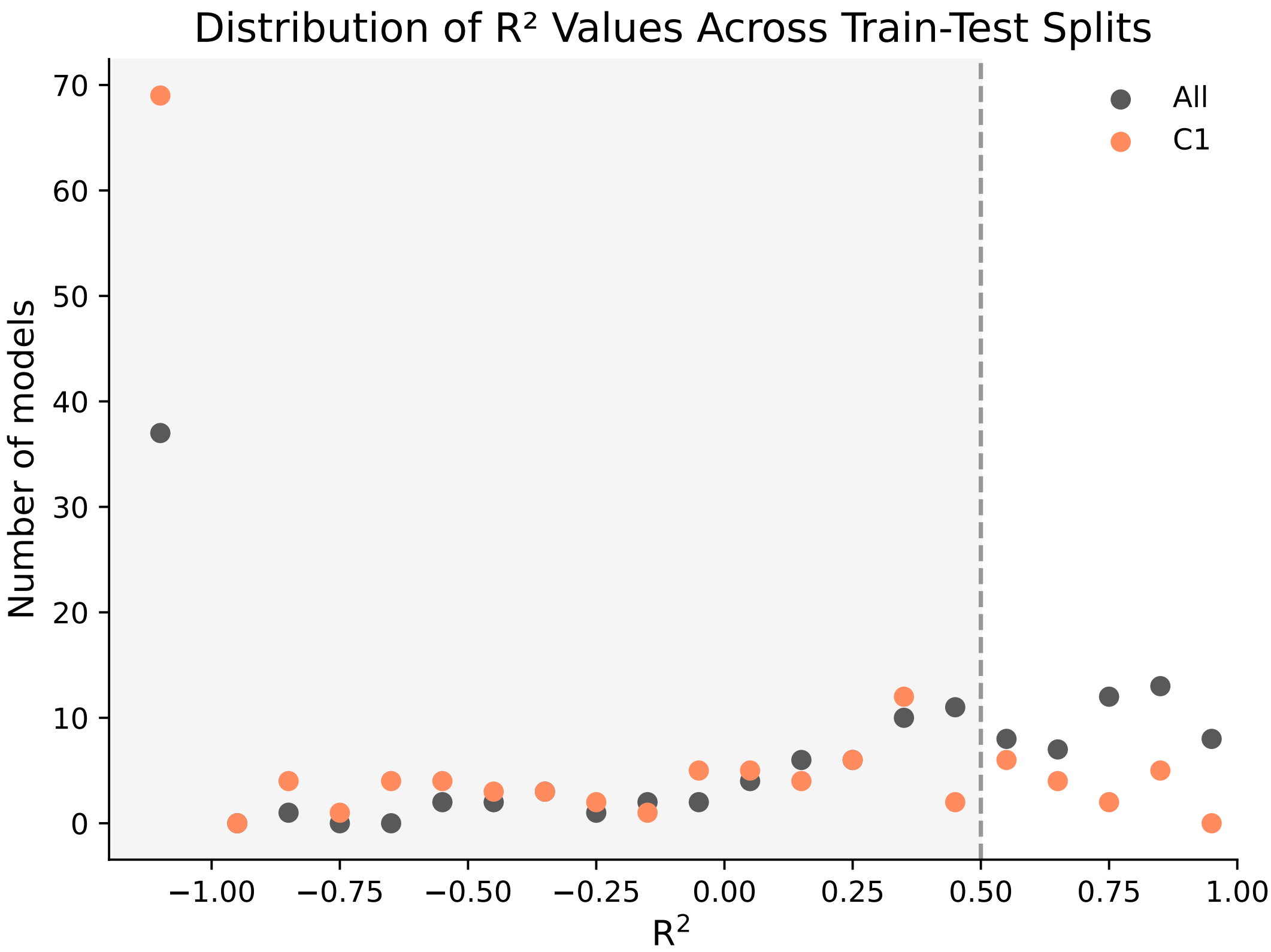


**S10 Fig. Distribution of $R^2$ Values Across Train-Test Splits.** Distribution of $R^2$ values across 142 rolling train-test splits for models using 'All' (black) and C1 (orange) search terms. The x-axis represents $R^2$ values, binned in increments of 0.1, while the y-axis shows the number of models falling within each bin. For clarity, values below -1 are binned together on the left. The distribution highlights significant variability in predictive performance, with some models achieving high $R^2$ values while others exhibit near-zero or negative scores. This variability suggests that model performance is highly sensitive to the train-test split selection.
