# Supplementary material for: Learning from pandemics: Feature selection and clustering for robust digital disease nowcasting
Source: PLoS One. 2026 Oct 1;21(10):e0358063. doi: 10.1371/journal.pone.0358063 (PMC13630223; doi:10.1371/journal.pone.0358063)
Supplement: Supplementary file 11 [file pone.0358063.s011.pdf]

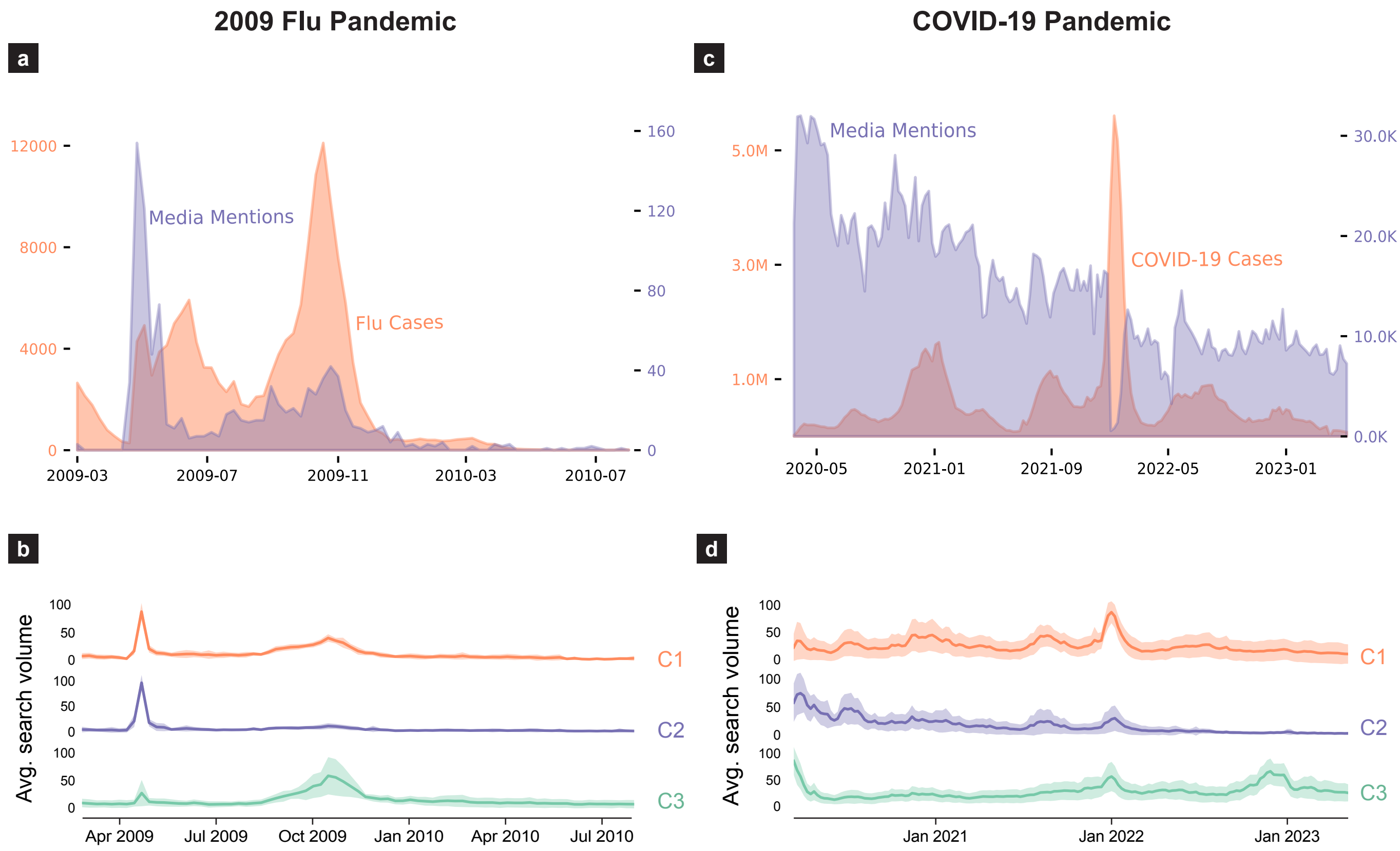

S11 Fig. Temporal relationship between media mentions, case numbers, and search term clusters during the 2009 Flu and COVID-19 pandemics. (a, c) Time series of reported infection cases (orange) and media mentions (purple) for flu (a) and COVID-19 (c). Media mentions tend to peak earlier in the pandemic period, while case numbers rise later and persist with multiple waves. (b, d) The temporal evolution of the three search term clusters (C1: orange, C2: purple, C3: green). C1 search trends closely follow infection case dynamics, while C2 exhibits similarities with media coverage patterns.
