# Supplementary material for: Learning from pandemics: Feature selection and clustering for robust digital disease nowcasting
Source: PLoS One. 2026 Oct 1;21(10):e0358063. doi: 10.1371/journal.pone.0358063 (PMC13630223; doi:10.1371/journal.pone.0358063)
Supplement: Supplementary file 12 [file pone.0358063.s012.pdf]

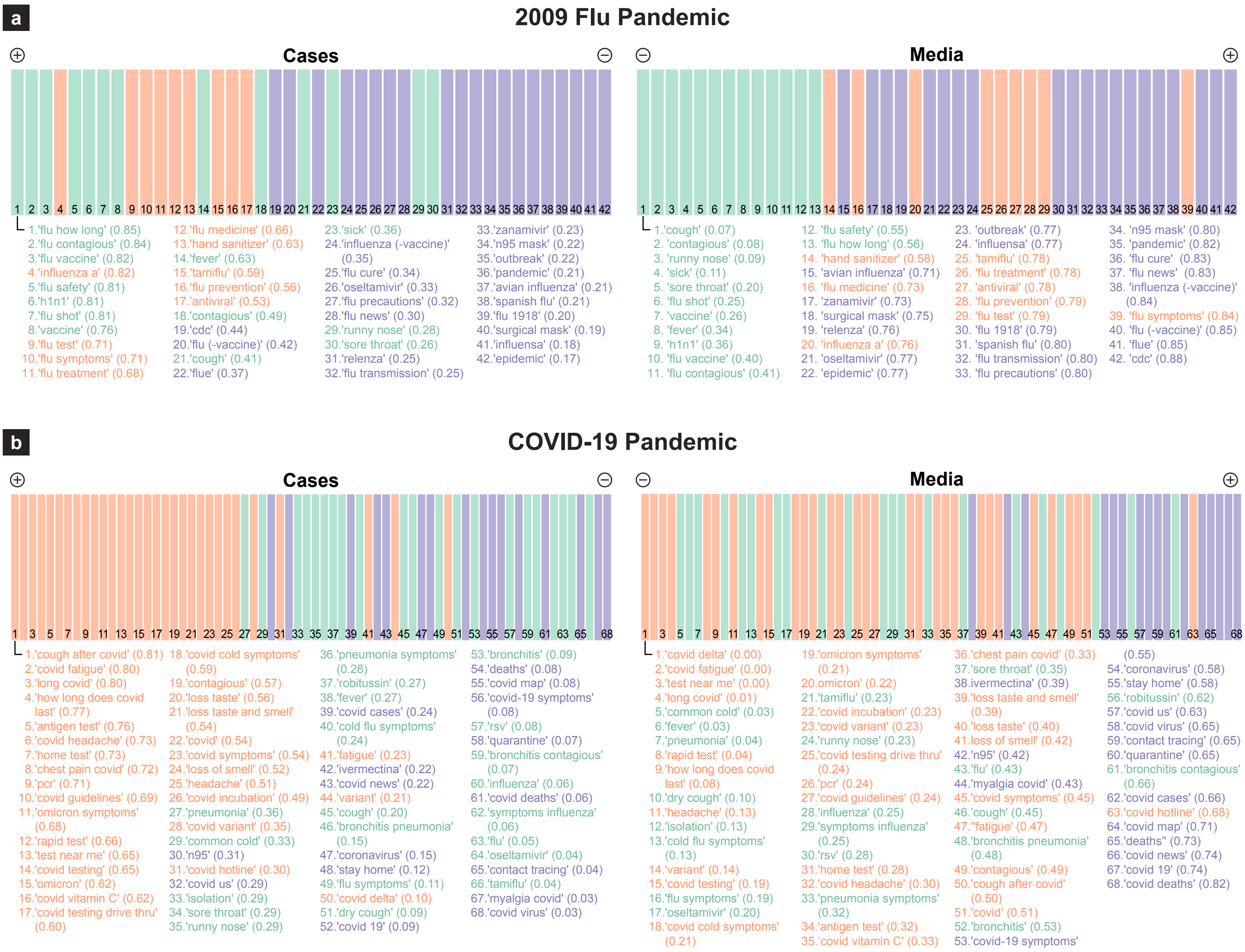

S12 Fig. Ranking of search terms by correlation with infection cases and media mentions for both pandemics. Ordering of search terms corresponding to Fig 3C. For each pandemic (2009 Flu and COVID-19), terms are ranked left to right by their correlation values. For cases, terms are arranged from the highest correlation (left) to the lowest correlation (right), as indicated by the "+" and "-" signs above the color bars. For media, the ranking is reversed, with terms ordered from the lowest correlation (left) to the highest (right). Clusters are color-coded as follows: C1 (orange), C2 (purple), and C3 (green). The exact correlation values for each term with cases and media are shown in parentheses.
