# Supplementary material for: Learning from pandemics: Feature selection and clustering for robust digital disease nowcasting
Source: PLoS One. 2026 Oct 1;21(10):e0358063. doi: 10.1371/journal.pone.0358063 (PMC13630223; doi:10.1371/journal.pone.0358063)
Supplement: Supplementary file 13 [file pone.0358063.s013.pdf]

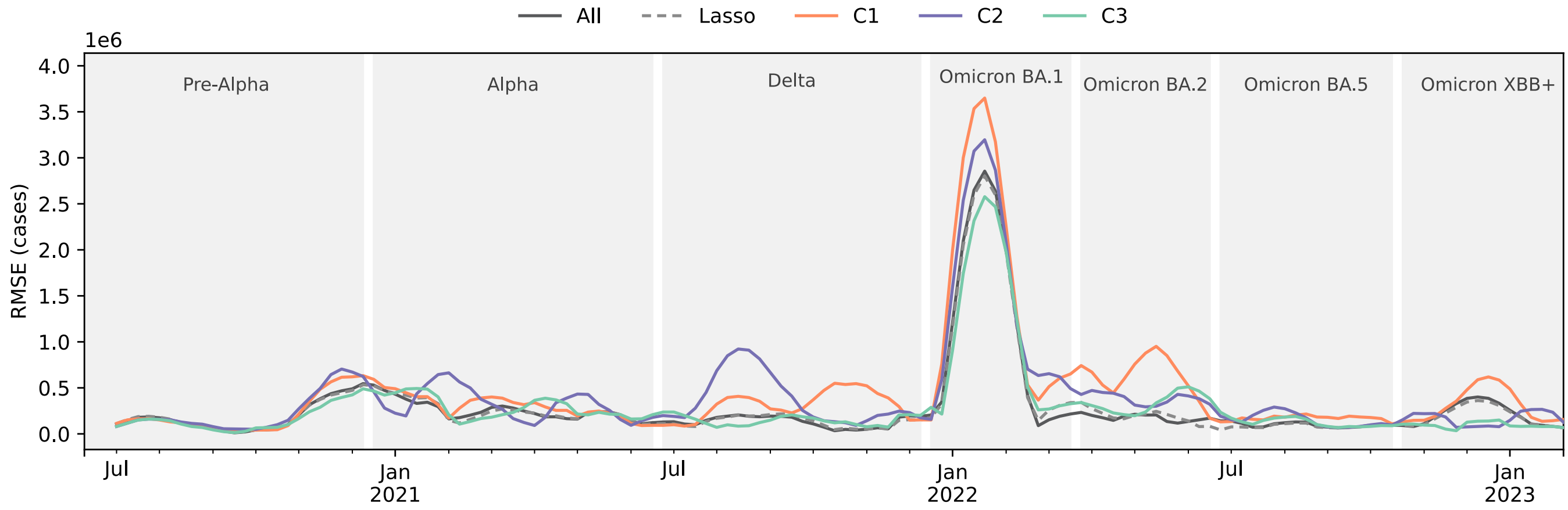

S13 Fig. Temporal instability across COVID-19 variants. Rolling RMSE (4-week window) for Linear Regression models using all search terms (black) and individual clusters (C1–C3; orange, purple, green), with Lasso shown as dashed grey. Shaded vertical bands mark dominant variant phases (Pre-Alpha through Omicron XBB +). Model error increases sharply during the Omicron BA.1 transition (winter 2021–2022), confirming a temporary degradation in predictive performance coinciding with shifts in testing behavior and symptom perception. We use a 4-week window as a compromise: longer than 2 weeks, it better attenuates weekly reporting cycles, holidays, and backlogs, but also shorter than 6+ weeks, preserving the timing and amplitude of month-scale transitions in dominant variants.
