# Supplementary material for: Learning from pandemics: Feature selection and clustering for robust digital disease nowcasting
Source: PLoS One. 2026 Oct 1;21(10):e0358063. doi: 10.1371/journal.pone.0358063 (PMC13630223; doi:10.1371/journal.pone.0358063)
Supplement: Supplementary file 14 [file pone.0358063.s014.pdf]

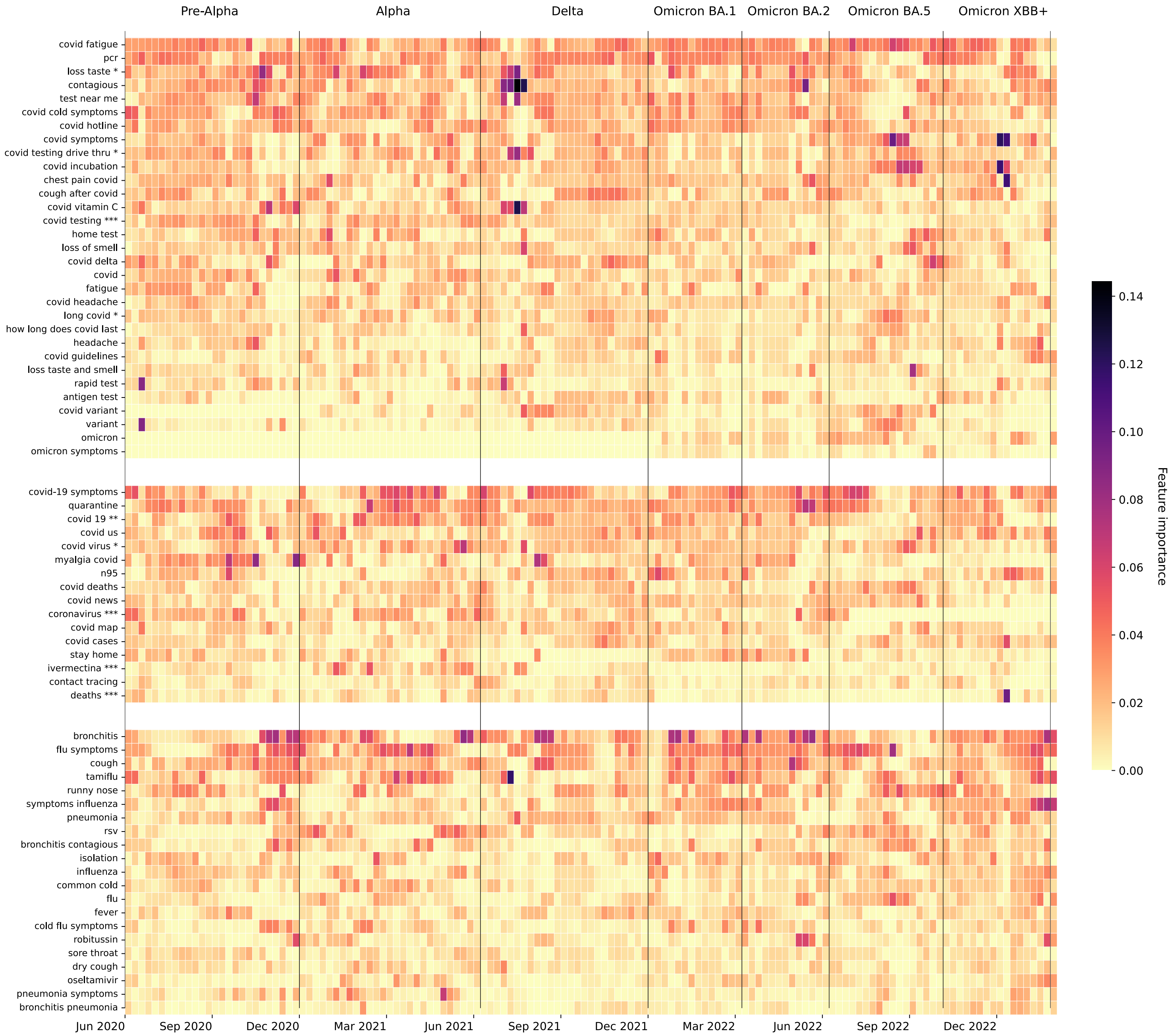

S14 Fig. Temporal evolution of feature importance across COVID-19 variant phases. Weekly Random Forest feature importances for all search terms (ALL model), grouped by previously defined clusters (C1–C3). Each row represents a search term, and each column corresponds to a week between March 2020 and early 2023. Colors indicate the relative contribution of each term to the model's predictions of COVID-19 cases. Vertical lines mark the dominant variant phases (Pre-Alpha through Omicron XBB +). Asterisks next to term labels indicate a significant decline in feature importance after January 2022, according to the one-tailed Mann–Whitney U test (Benjamini–Hochberg corrected): $*p < 0.05$, $**p < 0.01$, $***p < 0.001$. The analysis seem to show that symptom-specific terms such as *loss of smell* and *loss taste* lost predictive relevance after the onset of Omicron, whereas general COVID-related terms remained relatively stable.
