# Supplementary material for: Learning from pandemics: Feature selection and clustering for robust digital disease nowcasting
Source: PLoS One. 2026 Oct 1;21(10):e0358063. doi: 10.1371/journal.pone.0358063 (PMC13630223; doi:10.1371/journal.pone.0358063)
Supplement: Supplementary file 15 [file pone.0358063.s015.pdf]

Table S1: Search term clusters for the Flu pandemic.

**Cluster**	**Flu Terms**
C1	antiviral, flu medicine, flu prevention, flu symptoms, flu test, flu treatment, hand sanitizer, influenza a, tamiflu
C2	avian influenza, cdc, epidemic, flu (-vaccine), flu 1918, flu cure, flu news, flu precautions, flu transmission, flue, influensa, influenza (-vaccine), n95 mask, oseltamivir, outbreak, pandemic, relenza, spanish flu, surgical mask, zanamivir
C3	contagious, cough, fever, flu contagious, flu how long, flu safety, flu shot, flu vaccine, h1n1, runny nose, sick, sore throat, vaccine
