# Supplementary material for: Learning from pandemics: Feature selection and clustering for robust digital disease nowcasting
Source: PLoS One. 2026 Oct 1;21(10):e0358063. doi: 10.1371/journal.pone.0358063 (PMC13630223; doi:10.1371/journal.pone.0358063)
Supplement: Supplementary file 16 [file pone.0358063.s016.pdf]

Table S2: Search term clusters for the COVID-19 pandemic.

| **Cluster** | **COVID-19 Terms** |
|---|---|
| C1 | antigen test, chest pain covid, contagious, cough after covid, covid, covid cold symptoms, covid delta, covid fatigue, covid guidelines, covid headache, covid hotline, covid incubation, covid symptoms, covid testing, covid testing drive thru, covid variant, covid vitamin C, fatigue, headache, home test, how long does covid last, long covid, loss of smell, loss taste, loss taste and smell, omicron, omicron symptoms, pcr, rapid test, test near me, variant |
| C2 | contact tracing, coronavirus, covid 19, covid cases, covid deaths, covid map, covid news, covid us, covid virus, COVID-19 symptoms, deaths, ivermectina, myalgia covid, n95, quarantine, stay home |
| C3 | bronchitis, bronchitis contagious, bronchitis pneumonia, cold flu symptoms, common cold, cough, dry cough, fever, flu, flu symptoms, influenza, isolation, oseltamivir, pneumonia, pneumonia symptoms, robitussin, rsv, runny nose, sore throat, symptoms influenza, tamiflu |
