# Supplementary material for: Learning from pandemics: Feature selection and clustering for robust digital disease nowcasting
Source: PLoS One. 2026 Oct 1;21(10):e0358063. doi: 10.1371/journal.pone.0358063 (PMC13630223; doi:10.1371/journal.pone.0358063)
Supplement: Supplementary file 17 [file pone.0358063.s017.pdf]

Period	End of training	Start of test
P1	2013-07-21	2013-09-15
P2	2014-07-20	2014-08-31
P3	2015-09-06	2015-11-01
P4	2016-09-04	2016-09-11
P5	2017-07-23	2017-09-10
P6	2018-07-29	2018-09-16

Table S3: Training and test period splits for models using search terms collected during the 2009 flu pandemic to predict seasonal outbreaks. These splits were applied to both Linear Regression and Random Forest models.
