# Supplementary material for: Learning from pandemics: Feature selection and clustering for robust digital disease nowcasting
Source: PLoS One. 2026 Oct 1;21(10):e0358063. doi: 10.1371/journal.pone.0358063 (PMC13630223; doi:10.1371/journal.pone.0358063)
Supplement: Supplementary file 19 [file pone.0358063.s019.pdf]

Table S5: Bias-Variance trade-off for Flu (Linear Regression and Random Forest)

Cluster	Bias (LR)	Variance (LR)	Bias (RF)	Variance (RF)
All	0.7744	0.2256	0.952	0.048
C1	0.9276	0.0724	0.953	0.047
C2	0.8608	0.1392	0.914	0.086
C3	0.9375	0.0625	0.958	0.042
