# Supplementary material for: Learning from pandemics: Feature selection and clustering for robust digital disease nowcasting
Source: PLoS One. 2026 Oct 1;21(10):e0358063. doi: 10.1371/journal.pone.0358063 (PMC13630223; doi:10.1371/journal.pone.0358063)
Supplement: Supplementary file 20 [file pone.0358063.s020.pdf]

Table S6: Bias-Variance trade-off for COVID-19 (Linear Regression and Random Forest)

Cluster	Bias (LR)	Variance (LR)	Bias (RF)	Variance (RF)
All	0.5091	0.4909	0.9329	0.0671
C1	0.5279	0.4721	0.9457	0.0543
C2	0.5066	0.4934	0.9388	0.0612
C3	0.5223	0.4777	0.9349	0.0651
