# Supplementary material for: Learning from pandemics: Feature selection and clustering for robust digital disease nowcasting
Source: PLoS One. 2026 Oct 1;21(10):e0358063. doi: 10.1371/journal.pone.0358063 (PMC13630223; doi:10.1371/journal.pone.0358063)
Supplement: Supplementary file 21 [file pone.0358063.s021.pdf]

Table S7: Reproducibility of Google Trends extraction for *flu* terms: *pytrends* vs. official exports from the Google Trends web interface. Pearson and Spearman correlations and mean absolute difference (MAD) are reported for the H1N1 window (US, 2009–03–01 to 2010–08–01; 40–44 overlapping weeks). Correlations are high overall (median Pearson = 0.994).

Term	Pearson	Spearman	MAD
flu how long	0.991	0.917	15.383
flu news	0.994	0.944	0.632
flu shot	0.997	0.968	1.768
flu symptoms	0.995	0.966	0.907
flu vaccine	0.997	0.982	1.514
h1n1	0.998	0.994	1.204
influenza a	0.971	0.935	11.327
runny nose	0.936	0.941	51.020
