# Supplementary material for: Learning from pandemics: Feature selection and clustering for robust digital disease nowcasting
Source: PLoS One. 2026 Oct 1;21(10):e0358063. doi: 10.1371/journal.pone.0358063 (PMC13630223; doi:10.1371/journal.pone.0358063)
Supplement: Supplementary file 22 [file pone.0358063.s022.pdf]

Table S8: Reproducibility of Google Trends extraction using *pytrends* versus official exports using Google Trends online platform (two queries per cluster). Pearson and Spearman correlations and mean absolute difference (MAD) are shown. All correlations > 0.95, confirming negligible impact of Google Trends sampling variability.

Term	Pearson	Spearman	MAD
covid symptoms	0.985	0.984	2.55
pcr	0.994	0.988	1.30
covid news	0.982	0.986	3.37
quarantine	0.986	0.989	2.41
flu symptoms	0.979	0.958	5.52
sore throat	0.970	0.948	11.22
