# Supplementary material for: Learning from pandemics: Feature selection and clustering for robust digital disease nowcasting
Source: PLoS One. 2026 Oct 1;21(10):e0358063. doi: 10.1371/journal.pone.0358063 (PMC13630223; doi:10.1371/journal.pone.0358063)
Supplement: Supplementary file 23 [file pone.0358063.s023.pdf]

Table S9: Performance metrics of Lasso Regression across periods (P1–P6), including R^2, RMSE, Bias, and Variance. The last row shows the average performance.

**Model**	**Period**	$\mathbf{R^2}$	**RMSE**	**Bias**	**Variance**	**Pearson**
Lasso	1	0.904203	0.508189	0.951993	0.048007	0.983222
Lasso	2	0.872248	0.553815	0.974583	0.025417	0.986054
Lasso	3	0.920076	0.429158	0.974363	0.025637	0.984991
Lasso	4	0.967698	0.284454	0.842583	0.157417	0.985164
Lasso	5	0.965986	0.282358	0.869310	0.130690	0.990324
Lasso	6	0.932901	0.330100	0.979265	0.020735	0.990612
**Summary**	–	0.9272	0.3980	0.9320	0.0680	0.9867
