# Supplementary material for: Learning from pandemics: Feature selection and clustering for robust digital disease nowcasting
Source: PLoS One. 2026 Oct 1;21(10):e0358063. doi: 10.1371/journal.pone.0358063 (PMC13630223; doi:10.1371/journal.pone.0358063)
Supplement: Supplementary file 24 [file pone.0358063.s024.pdf]

Table S10: Lasso regression weights assigned to each search term across different periods (P1–P6). Search terms are grouped according to hierarchical clustering (C1, C2, C3).

**Search terms**	**P1**	**P2**	**P3**	**P4**	**P5**	**P6**
**C1**						
antiviral	0	8.47	6.36	0.67	-3.44	0
flu medicine	0	30.39	10.40	34.74	88.99	59.43
flu prevention	-68.77	0	0	-71.27	0	0
flu symptoms	0	-64.96	-50.10	-134.92	-312.76	-172.01
flu test	2.71	0	0	31.54	113.78	50.07
flu treatment	0	0	0	-36.78	-69.98	-79.55
hand sanitizer	0	3.12	0	-3.63	-2.71	-13.67
influenza a	202.85	193.92	219.85	300.81	249.92	283.06
tamiflu	0	0	0	0	0	0
**C2**						
avian influenza	0	-19.33	-3.17	-12.54	-9.58	0
cdc	0	-0.44	0	9.76	0	0
epidemic	-86.06	-55.99	-54.97	0	-18.85	0
flu (-vaccine)	0	0	0	0	0	0
flu 1918	0	0	0	0	0	0
flu cure	0	16.41	0	0	0	0
flu news	0	0	0	0	0	0
flu precautions	0	27.76	0	0	0	0
flu transmission	0	-8.80	0	-26.37	-39.04	0
flue	0	0	0	0	0	0
influensa	0	0	0	0	0	0
influenza (-vaccine)	0	0	0	0	0	0
n95 mask	0	0	0	0	0	0
oseltamivir	0	0	0	-53.23	17.21	0
outbreak	0	0	12.85	4.45	6.11	2.83
pandemic	0	0	0	-58.79	0	0
relenza	0	0	0	0	0	0
spanish flu	-20.96	-23.02	-28.78	-39.42	-22.11	-15.51
surgical mask	0	-22.16	-9.17	-2.95	0	0
zanamivir	0	0	0	0	0	0
**C3**						
contagious	-8.46	-4.69	0	0	-7.96	-2.46
cough	12.03	19.42	19.61	22.94	31.95	27.93
fever	5.49	10.91	1.87	12.63	23.50	8.60
flu contagious	46.17	30.97	48.40	5.06	20.41	6.61
flu how long	4.17	-4.71	0	0	0	0
flu safety	-6.34	-26.98	-19.16	0	0	-3.72
flu shot	-9.74	-9.85	-14.53	-19.81	-28.01	-26.32
flu vaccine	0	0	0	14.43	0	0
h1n1	0	-42.42	-130.78	-148.95	0	0
runny nose	0	-6.02	-1.64	-17.14	-10.18	-13.30
sick	0	-5.72	-24.12	3.36	-4.20	0
sore throat	-5.94	0	-6.48	-2.64	-9.82	0
vaccine	0	0	5.57	3.64	1.48	0
