# Supplementary material for: Learning from pandemics: Feature selection and clustering for robust digital disease nowcasting
Source: PLoS One. 2026 Oct 1;21(10):e0358063. doi: 10.1371/journal.pone.0358063 (PMC13630223; doi:10.1371/journal.pone.0358063)
Supplement: Supplementary file 25 [file pone.0358063.s025.pdf]

Table S11: Performance comparison of random feature selection models using 9 features across different flu seasons. Includes comparison to C1-only model performance and the percentile of the C1 model over the distribution of random models.

**Method**	**Season**	$\mathbf{R^2}$	**MSE**	**Variance**	**Bias**^{2}	**Percentile Over Random**
Random (9 features)	1	0.652	3.672	6.36e+18	3.20e+16	99.9
	2	0.641	4.699	2.50e+19	1.41e+17	95.6
	3	0.664	2.649	0.125	2.607	98.7
	4	0.664	2.694	0.154	2.648	99.7
	5	0.711	1.916	0.090	1.912	91.0
	6	0.835	0.332	0.014	0.331	62.7
