# Supplementary material for: Learning from pandemics: Feature selection and clustering for robust digital disease nowcasting
Source: PLoS One. 2026 Oct 1;21(10):e0358063. doi: 10.1371/journal.pone.0358063 (PMC13630223; doi:10.1371/journal.pone.0358063)
Supplement: Supplementary file 26 [file pone.0358063.s026.pdf]

Period	R^2	MSE	$Bias^2$	Variance
1	0.912964	0.900291	0.978909	0.021091
2	0.893321	1.429616	0.987725	0.012275
3	0.900820	0.765033	0.989954	0.010046
4	0.949273	0.426003	0.951774	0.048226
5	0.970067	0.203158	0.953326	0.046674
6	0.952828	0.099116	0.966712	0.033288

Table S12: Linear regression model performance when selecting search terms based on correlation with cases during the pandemic period. The table presents R^2 values for the model trained using the most correlated search terms across the full pandemic period, along with Bias-Variance tradeoff and Mean Squared Error (MSE). The selected search terms were: flu contagious, influenza a, flu how long, flu safety, and flu vaccine.
