# Supplementary material for: Learning from pandemics: Feature selection and clustering for robust digital disease nowcasting
Source: PLoS One. 2026 Oct 1;21(10):e0358063. doi: 10.1371/journal.pone.0358063 (PMC13630223; doi:10.1371/journal.pone.0358063)
Supplement: Supplementary file 27 [file pone.0358063.s027.pdf]

Period	Best_R2	Best_Num_Features	Best_Features	Final_MSE	Final_Bias	Final_Variance
1	0.964301	18	influenza a, tamiflu, flu contagious, flu cure, flu treatment, oseltamivir, flu medicine, flu test, flu symptoms, cough, relenza, fever, runny nose, contagious, sore throat	0.621497	0.562171	0.437829
2	0.933386	34	influenza a, tamiflu, flu contagious, flu cure, flu treatment, flu medicine, oseltamivir, flu symptoms, flu test, cough, relenza, fever, contagious, flu news, flu precautions, runny nose, sore throat, flu transmission, pandemic, avian influenza, surgical mask, n95 mask	1.178644	0.755472	0.244528
3	0.953725	9	influenza a, tamiflu, flu contagious, flu cure, flu treatment, flu medicine, flu test, oseltamivir, flu symptoms	0.429933	0.849248	0.150752
4	0.954374	1	influenza a	0.374314	0.954028	0.045972
5	0.973975	1	influenza a	0.180602	0.953434	0.046566
6	0.954989	1	influenza a	0.090958	0.980741	0.019259

Table S13: Linear regression model performance when selecting search terms based on seasonal correlation. R^2 values for the model trained using the most correlated search terms per season (P1–P6), Bias-Variance tradeoff, and Mean Squared Error (MSE), and the search terms used for each period. This table highlights that the required feature set varies considerably across seasons.
