# Supplementary material for: Learning from pandemics: Feature selection and clustering for robust digital disease nowcasting
Source: PLoS One. 2026 Oct 1;21(10):e0358063. doi: 10.1371/journal.pone.0358063 (PMC13630223; doi:10.1371/journal.pone.0358063)
Supplement: Supplementary file 28 [file pone.0358063.s028.pdf]

Table S14: Mean RMSE and R^2 by dominant COVID-19 variant phase for Linear Regression models. Higher RMSE and lower R^2 indicate reduced predictive accuracy.

**Variant phase**	**Model**	**RMSE**	$\mathbf{R^2}$
Pre-Alpha	All	2.71×10^5	0.523
Pre-Alpha	Lasso	2.67×10^5	0.539
Pre-Alpha	C1	3.18×10^5	0.344
Pre-Alpha	C2	3.19×10^5	0.338
Pre-Alpha	C3	2.32×10^5	0.650
Alpha	All	2.39×10^5	0.748
Alpha	Lasso	2.41×10^5	0.743
Alpha	C1	2.96×10^5	0.613
Alpha	C2	3.51×10^5	0.455
Alpha	C3	2.94×10^5	0.620
Delta	All	1.47×10^5	0.714
Delta	Lasso	1.44×10^5	0.723
Delta	C1	3.51×10^5	−0.632
Delta	C2	4.55×10^5	−1.746
Delta	C3	1.49×10^5	0.706
Omicron BA.1	All	1.56×10^6	0.317
Omicron BA.1	Lasso	1.53×10^6	0.338
Omicron BA.1	C1	2.04×10^6	−0.174
Omicron BA.1	C2	1.80×10^6	0.092
Omicron BA.1	C3	1.42×10^6	0.433
Omicron BA.2	All	1.70×10^5	0.358
Omicron BA.2	Lasso	1.73×10^5	0.339
Omicron BA.2	C1	6.17×10^5	−7.408
Omicron BA.2	C2	3.65×10^5	−1.945
Omicron BA.2	C3	3.46×10^5	−1.640
Omicron BA.5	All	9.16×10^4	0.834
Omicron BA.5	Lasso	8.91×10^4	0.843
Omicron BA.5	C1	1.71×10^5	0.421
Omicron BA.5	C2	1.61×10^5	0.489
Omicron BA.5	C3	1.20×10^5	0.717
Omicron XBB +	All	2.42×10^5	−6.960
Omicron XBB +	Lasso	2.21×10^5	−5.611
Omicron XBB +	C1	3.58×10^5	−16.424
Omicron XBB +	C2	1.82×10^5	−3.478
Omicron XBB +	C3	9.81×10^4	−0.306
