# Supplementary material for: Learning from pandemics: Feature selection and clustering for robust digital disease nowcasting
Source: PLoS One. 2026 Oct 1;21(10):e0358063. doi: 10.1371/journal.pone.0358063 (PMC13630223; doi:10.1371/journal.pone.0358063)
Supplement: Supplementary file 29 [file pone.0358063.s029.pdf]

PLOS ONE: Review assignment for PONE-D-25-28523

18 September 2025

I am pleased to review this article as I have been involved previously in textual Big Data analytics using API-access to Google texts global data storage.
https://link.springer.com/article/10.1007/s11625-020-00889-5

This kind of study can be called API-sociology, it combines computational linguistics approach with computational social science and (or) digital epidemiology.
https://accscience.com/journal/GHES/2/1/10.36922/ghes.2148

In my opinion, this research was done well, but the results of the work have too narrow a scope of the entire spectrum of new conclusions that could have been made. The authors identified an improved list of search words compared to Google Flu Trends, and also, by applying several mathematical tools, the authors identified the most relevant cluster of words C1 for predicting the onset of a seasonal flu or COVID epidemic. And although the authors mention social behavior in the article, which is determined through text analytics, and highlight differences in word clusters in their relationship to actual disease incidence or reactions to media reports (disease-related and attention-related search activity), this analytical aspect of the study is presented sparingly in comparison with the exhaustive technical part of the article. At the current stage of text Big Data analytics and digital trace analysis, the very use of cluster analysis is not the new to scientific research. Moreover, in similar studies, various authors emphasize the possibility of combining different Big Data sources. And of particular interest is the search for new digital traces – Dark Data – that have never been analyzed before and that have for the first time demonstrated correlations relevant to the problem of epidemics.

The authors themselves state in the article that «future work should explore the integration of additional data sources, such as mobility data, syndromic surveillance, and social media signals, to enhance robustness and generalizability» of model. But the authors did not propose the extended model with other types of data. Also, the authors mention that «the inexpensive nature and scalability of this method (search words) may make it particularly useful in low-resource settings where traditional disease surveillance is limited». I don't think it's very logical to contrast digital trace analysis methods with the lack of traditional resources, since the digital traces of a growing digital infrastructure of society are not so much about cheaper analysis, but rather about fundamentally new opportunities for obtaining insights faster and with greater predictive potential than analog studies. Big digital data, its velocity, variety, and ever-growing volume, should reveal the new discoveries in the collective behavior of society. There is also a lack of a linguistic approach to analyzing the social processes behind a particular search term, which is

especially important given the plasticity of language and the change of terms over time. Words can change, and processes then need to be guessed through new words.

It should be noted that this expression is not entirely correct: « The rationale being that if a cluster strongly correlates with case numbers, its terms are more likely to track actual disease burden, whereas a high correlation with media mentions suggests external information is shaping search behavior». The authors apparently meant that if a cluster strongly correlates with case numbers, its terms are more likely to actual the epidemiological burden on the population.

Overall, it is important to note the strong positive aspect of the study, which lies in the use of mathematical tools and the identification of relevant clusters of search terms. However, given the development of diverse digital infrastructure in the world and the existing complex models, a simple model with only search queries seems insufficient. The study requires major revision.

Olga Kolesnichenko, M.D., Candidate of Medical Sciences, Moscow

https://orcid.org/0000-0002-4523-6485
https://www.researchgate.net/profile/Olga-Kolesnichenko
