# Supplementary material for: Learning from pandemics: Feature selection and clustering for robust digital disease nowcasting
Source: PLoS One. 2026 Oct 1;21(10):e0358063. doi: 10.1371/journal.pone.0358063 (PMC13630223; doi:10.1371/journal.pone.0358063)
Supplement: Supplementary file 33 [file pone.0358063.s034.pdf]

PLOS One

Learning from Pandemics: Feature Clustering for Robust Digital Disease Nowcasting

--Manuscript Draft--

Manuscript Number:	PONE-D-25-28523R2
Article Type:	Research Article
Full Title:	Learning from Pandemics: Feature Clustering for Robust Digital Disease Nowcasting
Short Title:	Feature Clustering for Digital Nowcasting
Corresponding Author:	Joana Gonçalves-Sá, Ph.D.<br>LIP: Laboratorio de Instrumentacao e Fisica Experimental de Particulas<br>Lisbon, PORTUGAL
Keywords:	digital epidemiology; Now-casting; Search engine data; Behavioral confounders; Feature curation; Pandemic surveillance; Model overfitting; Infectious diseases monitoring; Public health monitoring
Abstract:	Disease now-casting increasingly relies on non-traditional data, such as online activity, which offer fast, inexpensive insights but are often noisy, unstructured, and influenced by behavioral confounders. Models using search engine data have frequently suffered from overfitting and unstable performance. However, pandemics may help distinguish true disease signals from behavioral noise, as both tend to surge during outbreaks. We propose a hybrid approach to improve prediction that combines model evaluation with behavioral insight. We apply simple supervised and unsupervised methods to different flu and COVID-19 datasets, using the 2009 H1N1 and 2020 COVID-19 pandemics as training periods. In both cases, selecting a curated subset of search terms improved model performance, reduced overfitting, and increased stability,<br>consistently outperforming models that used search terms indiscriminately.<br>These results suggest that feature curation informed by behavioral insights can enhance now-casting beyond purely statistical approaches. Our method offers a scalable<br>and adaptable framework for integrating digital epidemiology into public health surveillance.
Order of Authors:	Sara Mesquita
	João Loureiro
	Claudio Haupt-Vieira
	Lília Perfeito
	Joana Gonçalves-Sá, Ph.D.
Response to Reviewers:	We thank the Academic Editor and the reviewers for the time and effort invested in the review process and for their relevant comments and suggestions. We have carefully considered all comments and have revised the manuscript accordingly. Below, in blue, we provide a detailed, point-by-point response to each comment. Changes made to the manuscript are indicated in italics throughout this document and in blue in the manuscript.<br><br>Reviewer 3<br>The paper titled "Learning from Pandemics: Feature Selection for Robust Digital Disease Nowcasting" requires essential improvements. Some sections of the present version of the revision remained unclear and then reviewer is worrying about scientific and originality of the paper contents.<br>We thank R3 for the detailed comments and while we agree with several suggestions we respectfully try to justify why some others might be outside of the scope of the current work.<br>1-In abstract section, authors need to mention which Machine Learning Models (MLMs) were used in this study with their main purposes. The main findings along with its conditions of datasets can be merged to the abstract section.<br>To comply both with the reviewer's request and with the journal's word formatting

	requirements, the following sentence has been added to the abstract (in italics):<br>“We propose a hybrid approach to improve prediction that combines model evaluation with behavioral insight. We apply simple supervised and unsupervised methods to different flu and COVID-19 datasets, using the 2009 H1N1 and 2020 COVID-19 pandemics as training periods. In both cases, selecting a curated subset of search terms improved model performance, reduced overfitting, and increased stability, consistently outperforming models that used search terms indiscriminately.”<br>2-Introduction section requires diverse related research works, describing application of MLMs in the case of feature selection and capability of robust MLMs. Reviewer is now considering table containing literature review in this area whereas this table need a lot of major modifications.<br>For Feature Selection: -Unsupervised feature selection via graph-based proximity and structured autoencoder-like NMF -Joint sample-feature subspace learning via bidirectional reconstruction for feature selection For robustness: -Robust oblique projection and weighted NMF for hyperspectral unmixing -Simultaneous outlier detection and elimination in hyperspectral unmixing via weighted non-negative matrix tri-factorization<br>We thank the reviewer for these suggestions and carefully considered the recommended references. While some of these articles fall outside the scope of the present study, as the manuscript focuses on the application of machine learning methods to digital epidemiology and disease nowcasting rather than on methodological developments in machine learning algorithms, we see how the paper could benefit from a broader overview of recent work in this area. Accordingly, we have updated it with references covering (i) applications of machine learning in epidemiology, (ii) recent developments in feature selection methodologies, and (iii) recent advances in infectious disease forecasting using digital data. In particular, we have added:<br>1 - LLM-Assisted Keyword Curation for Early Disease Outbreak Detection Using Search Trends, by Brahmos and Princess (2025), which reinforces the importance of careful feature selection in search-based epidemiology and provides a complementary, LLM-assisted approach to keyword curation.<br>Added to the Introduction (Lines 19-23): "As the use of online search data for disease surveillance has matured, attention has shifted from whether these data are useful to how the most informative search terms should be selected. Even with recent advances in artificial intelligence, careful keyword curation remains essential for identifying the search terms that carry the most relevant information for disease prediction [19].”<br>2 - “Machine learning in epidemiology: An introduction, comparison with traditional methods, and a case study of predicting extreme longevity”, a recent review by Goldstein et al., which discusses the application of machine learning in epidemiology and reinforces the importance of selecting methods according to the epidemiological question being addressed<br>Added to the Discussion (Lines 663–665): “This is consistent with recent perspectives arguing that, in epidemiology, machine learning methods should be selected according to the question being addressed rather than solely on predictive performance [74].”<br>3 – “Mathematical methods in feature selection: A review”, by Kamalov et al. (2025), which classifies feature selection methods according to their mathematical foundations and discusses their respective strengths, limitations, and areas of application, providing additional context for the approaches evaluated in the present study.<br>4 – “Recent advances in feature selection and its applications”, by Li et al., which reviews recent developments in feature selection and highlights the importance of selecting relevant and non-redundant features to improve both predictive performance and model interpretability. This provides additional theoretical context for the behaviourally informed feature selection strategy proposed in the present study.<br>Citations 3 and 4 were added to the Introduction (Lines 88—90): "Recent reviews have emphasized that effective feature selection should identify informative predictors while reducing redundancy, thereby improving predictive performance, robustness, and model interpretability [52, 53].”<br>5 - Advancing real-time infectious disease forecasting using large language models, by Du et al. (2025), which provides a recent overview of advances in infectious disease forecasting using artificial intelligence and highlights the growing integration of digital and heterogeneous data sources to complement traditional surveillance systems.<br>Added to the Introduction (Lines 7--10): "As surveillance mechanisms relying solely on highly trained personnel are often expensive, limited, and slow, it has been extensively argued that traditional methods should be complemented with 'Digital Era' tools, such as online activity, mobility patterns, or digital contact-tracing [1, 2, 3, 4]."

Powered by Editorial Manager® and ProduXion Manager® from Aries Systems Corporation

	6 - Forecasting dengue cases through time-series modeling with Google Trends and deep neural networks, by Cheong et al. (2025), which demonstrates that parsimonious Google Trends feature sets can outperform larger collections of search terms in infectious disease forecasting. While their feature selection is based on statistical associations,<br>our work provides a complementary behavioural perspective that helps explain why the indiscriminate inclusion of search terms may reduce predictive performance.<br>Added to the Results (Lines 507--509): "This is consistent with recent work on dengue forecasting using Google Trends, where parsimonious search-term sets selected through statistical associations outperformed larger collections of search terms [72]."<br>3-Research gap and novelty should be deeply discussed in the introduction section.<br>We agree that clearly defining the research gap and novelty is important. To address this comment, we revised the final paragraph of the Introduction to more clearly distinguish our contribution from previous work. Specifically, we now explicitly identify the research gap and clarify the novelty of the proposed approach. The Introduction now reads:<br>"Recent reviews have emphasized that effective feature selection should identify informative predictors while reducing redundancy, thereby improving predictive performance, robustness, and model interpretability. However, relatively little attention has been paid to whether pandemic periods themselves can be exploited to improve feature selection. To address this gap, we propose a simple and explainable feature selection framework that leverages the high-intensity periods of pandemics as natural experiments to identify search terms with greater temporal robustness for disease nowcasting."(Lines 88–95).<br>4-Research organization, known as the last paragraph of introduction section should be deeply provided.<br>To address this comment, we expanded the final paragraph of the Introduction to include a brief overview of the organization of the manuscript. After introducing the research gap and the proposed method, we now briefly describe how the study is structured, from the presentation of the data sources and modelling approach to the evaluation of the proposed method and the discussion of its implications.<br>The Introduction now concludes with:<br>"To evaluate this approach, we first describe the data sources and modelling approach, then assess its performance across influenza and COVID-19 case studies, and finally discuss its implications for the design of robust and interpretable disease nowcasting systems." (Lines 98-101)<br>5- Delete subsections in the introduction section. And more, all the subsections should have had only single name as "Introduction" in which includes (i) importance of subject and background theorem, (ii) literature review with emphasize on major drawbacks of empirical/traditional methodologies equations, (iii) research gap and novelty, and (iv) research organization in which overview of the present research should be mentioned in a paragraph.<br>We ask the reviewer to confirm whether the current version of the manuscript complies with that request or offer more detail on how it should be improved.<br>6-Details of case study and some important discussions on the datasets should be detailed.<br>We ask the reviewer to confirm whether the current version of the manuscript complies with that request or offer more detail on how it should be improved.<br>7-Authors are emphatically recommended to use MLMs such as KNN, Model Tree (MT), AdaBoost, XGBoost, CatBoost, LightBoost, and RF for the training and testing stages.<br>Algorithms such as KNN, Model Trees, AdaBoost, XGBoost, CatBoost, and LightGBM are indeed powerful machine learning methods and can provide great predictive performance in many applications. However, since the primary objective of the present study was to evaluate whether behaviourally informed feature selection improves the robustness and generalizability of disease nowcasting models, we decided against testing a broad range of models and selected two complementary and widely used approaches: Linear Regression, as a simple and fully interpretable baseline, and Random Forest, as a nonlinear ensemble method capable of capturing more complex relationships.<br>The fact that the proposed feature selection strategy consistently improved performance across both model classes suggests that the observed gains arise from the quality of the selected features and not from a particular algorithm. While evaluating additional algorithms such as gradient boosting methods would certainly be

Powered by Editorial Manager® and ProduXion Manager® from Aries Systems Corporation

an interesting direction for future work, we respectfully argue that it falls outside the scope of the present study and would not substantially alter its main conclusions.
9-Descriptions on every MLM should be presented in a separate subsection along with the setting parameters of each predictive models and the corresponding justifications. In this section, authors should use AdaBoost, CatBoost, LighGBoost, XGBoost, KNN, SVM, RF, and other regression models based on machine learning techniques (ElasNet, Lasso, and Ridge).
To improve this section, authors need to use the material of the following papers:
- Vulnerability of the rip current phenomenon in marine environments using machine learning models
- Assessment of flood susceptibility in Minab County, Iran, through the integration of topographic, climatic, and land-surface indices using ensemble machine learning models
- Ultrasonic flowmeter fault early warning method based on Bayesian optimization XGBoost
- Evaluation of river water quality index using remote sensing and artificial intelligence models
- A Stacked Machine Learning Algorithm for Multi-Step Ahead Prediction of Soil Moisture
Please see our response to Comment 7 regarding the rationale for the selection of the predictive models evaluated in this study, as the same considerations apply here.
10- Instead using R2, authors are emphatically recommended to use Correlation of Coefficient (CC), RMSE, Mean Absolute Error (MAE), Mean Bias Error (MBE), Discrepancy Ratio (DR), and Scatter Index (SI) to this section. These statistical measures should be computed for the training and testing stages of the present predictive models. All the formulations and acceptable ranges of these statistical measures should be merged to this section. For this purpose, read the following papers and add them to the present revisions.
-A hybrid hourly natural gas demand forecasting method based on the integration of wavelet transform and enhanced Deep-RNN model
-A deep reinforcement learning-based method for predictive management of demand response in natural gas pipeline networks
-A Combinatorial Machine Learning Models -Based Method for Predicting the Viscosity-Temperature Relationship of Crude Oil
-Receiving More Accurate Predictions for Longitudinal Dispersion Coefficient in Water Pipelines: Training Group Method of Data Handling Using Extreme Learning Machine Conceptions
-A Method for Predicting the Gel Point of Mixed Crude Oil Based on Deep Learning
- A Large Language Model Based Knowledge Mining Method for Improving the Reliability of Fire Water Systems
- Optimization Method of Natural Gas Pipeline Network Emergency Dispatch Based on Particle Swarm Optimization
-Data-driven reliability assessment method of Integrated Energy Systems based on probabilistic deep learning and Gaussian mixture Model-Hidden Markov Model
-Interpretable Combinatorial Machine Learning-Based Shale Fracability Evaluation Methods
-Dynamic Prediction of Natural Gas Calorific Value Based on Deep Learning
-Water quality monitoring for coastal hypoxia: Integration of satellite imagery and machine learning models
-Dynamic damage functions for scour protection at mono-pile foundations: Application of ensemble machine learning models
-A new correlation for calculating carbon dioxide minimum miscibility pressure based on multi-gene genetic programming
We ask the reviewer to confirm whether the current version of the manuscript complies with that request. In addition to R2, and partially also in response to reviewer R1, we added bias-variance decomposition (Methods, Bias-variance trade-off subsection), and RMSE-based analyses, including phase-specific and rolling RMSE evaluations (Methods, Variant-stratified evaluation subsection; Results, Overfitting reduction, generalization, and predictive reliability in feature selection subsection; S14 Table).
11-Use Scatter plots, Taylor's diagrams, heat maps, and violin plots to illustrate the results of the training and testing stages.
We agree that visual assessment is very useful when evaluating models. In the current manuscript and Supporting Information, we already provide several complementary visualizations of model performance, including line plots comparing observed and

predicted trajectories for both Linear Regression and Random Forest models in the flu and COVID-19 analyses (S2–S3 and S5–S6 Figs), scatter plots relating residuals to observed incidence, line plots showing the temporal evolution of residuals, histograms illustrating residual and incidence distributions, and binned summary plots of mean residuals across incidence levels (S4 and S7 Figs). In the main manuscript, we also compare model performance across feature sets (Fig. 2). In addition, the manuscript includes heatmaps illustrating the longitudinal evolution of feature importance across SARS-CoV-2 variant periods, as requested by another reviewer.
It is our assessment that the current set of figures provides sufficient visual information to evaluate model performance and prediction errors across the training and testing settings. While Taylor diagrams and violin plots could provide an alternative way of visualizing some of these results, we are not sure that they would provide additional insight. If the reviewer feels that a particular aspect of model performance would benefit from additional visualization, we would appreciate more specific clarification so that we can better understand the concern and address it as effectively as possible.
12-The present results should be compared with those related papers (use more than 15 papers at least with quantitative and qualitative comparisons).
Please see answer to comment 2.

Reviewer #6:
Based on the manuscript contents and contexts, all the above questions seem to fulfill the PLOS requirements.
We thank the reviewer for the positive and constructive comments and overall recommendation to publish.
However, there are a few comments to the authors for consideration, which include: 1. The manuscript title is "Learning from Pandemics: Feature Selection for Robust Digital Disease Nowcasting." Discussions are focused on how to improve prediction that combines model evaluation with behavioral insight using curated features. Since hierarchical clustering is applied in data processing and the clustering results (C1, C2, C3) become the input for the rest of the analysis for 2009 H1N1, while for Covid-19 the predefined period (P1, P2, ..., P6) is applied, I would suggest revising the title to use "Feature Clustering" instead. For the "Feature Selection" to be used, discussions shall highlight how the number of searched terms is reduced in quantity (leaving only the significant terms) that would help improve the prediction. Consider this if it is viable.
We thank the reviewer for the comment. It is not only viable, but it also offers a better description of the work. We have updated the title as suggested.
2. Searched terms in the online may be an intercept between different aspects of symptoms, treatment, personal care, institutions, and pandemic-specific terms. As this led to redundancy, how does this study see this as a threat to the accuracy of nowcasting? Add your comments in the discussion if it has not been addressed.
We agree both with the note that search terms can have different natures and underlying motivations, and that redundancy is often a problem. We cluster similar terms and include two types of analysis (in the supplementary materials) in line with this point: LASSO regression and performance when selecting terms by correlation (pandemic and seasonal periods). We now also mention this in the discussion, as suggested.
It reads: (Lines 641–654) “In fact, including all available features can introduce variance that overwhelms the true signal. Instead, our approach prioritizes coherence, identifying subsets of search terms that behave consistently during outbreaks (clusters) and remain predictive beyond crisis contexts (...) That these pandemic-identified terms (particularly those in C1) continue to outperform others across seasonal flu periods suggests they capture underlying behavioral regularities that are robust across time. This finding stands in contrast to purely statistical feature selection methods, such as correlation filtering or LASSO regression, which may perform well within narrow windows but often yield unstable and inconsistent results across datasets”.
3. Redundancy among the searched terms is seen to be the source of bias-variance. For future nowcasting implementation, how is this issue to be addressed technically to increase predictability and accuracy? Include this in the discussion.
This is a very relevant point and in line with a previous comment from reviewer R1. We have added the following sentence to the discussion (in italics): “This also reinforces that feature selection alone does not guarantee temporal robustness; as search behaviour evolves, maintaining temporal robustness will require approaches that monitor changes in search patterns (e.g., feature drift) and rely on domain expertise for feature interpretation and validation.” (Lines 658-660)

	We also clarify that redundancy can be a source of bias-variance and the results section also reads (new text in italics): “when including too many or redundant predictors, models learn patterns that do not generalize beyond the training data” (Lines 466-467)<br><br>Again, we thank both the Editors and the Reviewers for their comments and hope that our answers address all the points raised during the review process.
**Additional Information:**	
**Question**	**Response**
**Competing Interests**<br><br>Use the instructions below to enter a competing interest statement for this submission. On behalf of all authors, disclose any competing interests that could be perceived to bias this work—acknowledging all financial support and any other relevant financial or non-financial competing interests.<br><br>This statement is required for submission and **will appear in the published article** if the submission is accepted. Please make sure it is accurate and that any funding sources listed in your Funding Information later in the submission form are also declared in your Financial Disclosure statement.<br><br>View published research articles from *PLOS ONE* for specific examples.<br><br>**NO authors have competing interests**<br><br>Enter: *The authors have declared that no competing interests exist.*<br><br>**Authors with competing interests**<br><br>Enter competing interest details beginning with this statement:<br><br>*I have read the journal's policy and the authors of this manuscript have the following competing interests: [insert competing interests here]*<br><br>* typeset	The authors have declared that no competing interests exist.

**Data Availability**<br><br>Authors are required to make all data underlying the findings described fully available, without restriction, and from the time of publication. PLOS allows rare exceptions to address legal and ethical concerns. See the PLOS Data Policy and FAQ for detailed information.<br><br>A Data Availability Statement describing where the data can be found is required at submission. Your answers to this question constitute the Data Availability Statement and **will be published in the article**, if accepted.<br><br>**Important:** Stating 'data available on request from the author' is not sufficient. If your data are only available upon request, select 'No' for the first question and explain your exceptional situation in the text box.<br><br>Do the authors confirm that all data underlying the findings described in their manuscript are fully available without restriction?	Yes - all data are fully available without restriction
Additional data availability information:	Tick here if the URLs/accession numbers/DOIs will be available only after acceptance of the manuscript for publication so that we can ensure their inclusion before publication.
**Financial Disclosure**<br><br>Enter a financial disclosure statement that describes the sources of funding for the work included in this submission. Review the submission guidelines for detailed requirements. View published research articles from *PLOS ONE* for specific examples.<br><br>This statement is required for submission and **will appear in the published article** if the submission is accepted. Please make sure it is accurate.	Yes

**Funded studies**<br>Enter a statement with the following details:<br>• Initials of the authors who received each award<br>• Grant numbers awarded to each author<br>• The full name of each funder<br>• URL of each funder website<br>• Did the sponsors or funders play any role in the study design, data collection and analysis, decision to publish, or preparation of the manuscript?<br><br>Did you receive funding for this work?	
Please add funding details.<br>as follow-up to "**Financial Disclosure**<br><br>Enter a financial disclosure statement that describes the sources of funding for the work included in this submission. Review the submission guidelines for detailed requirements. View published research articles from *PLOS ONE* for specific examples.<br><br>This statement is required for submission and **will appear in the published article** if the submission is accepted. Please make sure it is accurate.<br><br>**Funded studies**<br>Enter a statement with the following details:<br>• Initials of the authors who received each award<br>• Grant numbers awarded to each author<br>• The full name of each funder<br>• URL of each funder website<br>• Did the sponsors or funders play any role in the study design, data collection and analysis, decision to publish, or preparation of the manuscript?<br><br>Did you receive funding for this work?"	This study was funded by the Portuguese Foundation for Science and Technology (FCT) through the PhD fellowships SFRH/BD/139322/2018 and 2020.10157.BD awarded to CHV and SM, respectively. Additional support was provided by the FCT grant DSAIPA/AI/0087/2018 to JGS, and by the European Union – NextGenerationEU through the project grant 2024.07331.IACDC. The funders had no role in the study design, data collection, analysis, interpretation of results, or manuscript preparation.
Please select the country of your main research funder (please select carefully as in some cases this is used in fee calculation).	PORTUGAL - PT

Powered by Editorial Manager® and ProduXion Manager® from Aries Systems Corporation

as follow-up to "**Financial Disclosure**<br><br>Enter a financial disclosure statement that describes the sources of funding for the work included in this submission. Review the submission guidelines for detailed requirements. View published research articles from *PLOS ONE* for specific examples.<br><br>This statement is required for submission and **will appear in the published article** if the submission is accepted. Please make sure it is accurate.<br><br>**Funded studies**<br>Enter a statement with the following details:<br>• Initials of the authors who received each award<br>• Grant numbers awarded to each author<br>• The full name of each funder<br>• URL of each funder website<br>• Did the sponsors or funders play any role in the study design, data collection and analysis, decision to publish, or preparation of the manuscript?<br><br>Did you receive funding for this work?"	
**Ethics Statement**<br><br>Enter an ethics statement for this submission. This statement is required if the study involved:<br><br>• Human participants<br>• Human specimens or tissue<br>• Vertebrate animals or cephalopods<br>• Vertebrate embryos or tissues<br>• Field research<br><br>Write "N/A" if the submission does not require an ethics statement.<br><br>General guidance is provided below. Consult the submission guidelines for detailed instructions. **Make sure that all information entered here is included in the**	N/A

Powered by Editorial Manager® and ProduXion Manager® from Aries Systems Corporation

**Methods section of the manuscript.**<br><br>**Format for specific study types**<br><br>**Human Subject Research (involving human participants and/or tissue)**<br>• Give the name of the institutional review board or ethics committee that approved the study<br>• Include the approval number and/or a statement indicating approval of this research<br>• Indicate the form of consent obtained (written/oral) or the reason that consent was not obtained (e.g. the data were analyzed anonymously)<br><br>**Animal Research (involving vertebrate animals, embryos or tissues)**<br>• Provide the name of the Institutional Animal Care and Use Committee (IACUC) or other relevant ethics board that reviewed the study protocol, and indicate whether they approved this research or granted a formal waiver of ethical approval<br>• Include an approval number if one was obtained<br>• If the study involved *non-human primates*, add *additional details* about animal welfare and steps taken to ameliorate suffering<br>• If anesthesia, euthanasia, or any kind of animal sacrifice is part of the study, include briefly which substances and/or methods were applied<br><br>**Field Research**<br><br>Include the following details if this study involves the collection of plant, animal, or other materials from a natural setting:<br>• Field permit number<br>• Name of the institution or relevant body that granted permission	
**Data Availability**<br><br>Before publication, Authors are required to make fully available and without restriction all data underlying their findings. Sharing the data underlying your findings increases the visibility, credibility, and impact of your work, while supporting	All data and code are available at https://doi.org/10.5281/zenodo.21236065 for editorial analysis and will be made publicly accessible upon acceptance.

Powered by Editorial Manager® and ProduXion Manager® from Aries Systems Corporation

transparency and future scientific discovery. Please see our PLOS Data Policy page for detailed information on this policy.<br><br>A **Data Availability Statement**, detailing where the data can be accessed, is required at first submission. Insert your Data Availability Statement in the box below.<br><br>Please see the data reporting section of our submission guidelines for instructions on what you need to include in your Data Availability Statement. Examples of acceptable Data Availability Statements are included here for reference:<br>1. The minimal data set [and accompanying code, where generated] is available at [repository name] via [DOI hyperlink]<br>2. No data was generated by this study. The following existing data sources were used: [dataset name] from [dataset location] available via [web address or DOI hyperlink].<br>3. For Study Protocols: No datasets were generated or analysed during the current study. All relevant data from this study will be made available upon study completion.<br><br>*PLOS allows rare exemptions to address legal and ethical concerns. If you have legal or ethical restrictions, please use the box below to detail these in full sentences for the Journal team to consider.*<br><br>This statement will be typeset if the manuscript is accepted for publication.	

Manuscript
Click here to access/download;Manuscript;PLOS_One_Manuscript.pdf

Learning from Pandemics: Feature Clustering for Robust Digital Disease Nowcasting

Sara Mesquita[1,2], João Loureiro[1], Cláudio Haupt Vieira[3], Lília Perfeito[1], Joana Gonçalves-Sá[1,3,4*]

1 LIP – Laboratory for Instrumentation and Experimental Particle Physics, Avenida Prof. Gama Pinto 2, 1600-078 Lisboa, Portugal
2 NOVA Medical School, Universidade NOVA de Lisboa, Campo dos Mártires da Pátria 130, 1169-056 Lisboa, Portugal
3 NOVA School of Business and Economics, Universidade NOVA de Lisboa, Rua da Holanda, 2775-405 Carcavelos, Portugal
4 NOVA LINCS, Faculdade de Ciências e Tecnologia, Universidade NOVA de Lisboa, 2829-516 Caparica, Portugal

* Corresponding author: joanagsa@lip.pt

Abstract

Disease now-casting increasingly relies on non-traditional data, such as online activity, which offer fast, inexpensive insights but are often noisy, unstructured, and influenced by behavioral confounders. Models using search engine data have frequently suffered from overfitting and unstable performance. However, pandemics may help distinguish true disease signals from behavioral noise, as both tend to surge during outbreaks.

We propose a hybrid approach to improve prediction that combines model evaluation with behavioral insight. We apply simple supervised and unsupervised methods to different flu and COVID-19 datasets, using the 2009 H1N1 and 2020 COVID-19 pandemics as training periods. In both cases, selecting a curated subset of search terms improved model performance, reduced overfitting, and increased stability, consistently outperforming models that used search terms indiscriminately.

These results suggest that feature curation informed by behavioral insights can enhance now-casting beyond purely statistical approaches. Our method offers a scalable and adaptable framework for integrating digital epidemiology into public health surveillance.

Introduction

Infectious diseases present significant health risks worldwide. To mitigate these risks, public health institutions have established surveillance and response systems designed to detect and ideally stop outbreaks quickly and accurately. Typical monitoring includes complementary approaches, such as sentinel doctors and testing laboratories, but even when these mechanisms are effectively in place, detection and mitigation efforts can fail, leading to epidemics and, in rarer cases, pandemics. As surveillance mechanisms relying solely on highly trained personnel are often expensive, limited, and slow, it has been extensively argued that traditional methods should be complemented with ”Digital Era” tools, such as online activity, mobility patterns, or digital contact-tracing [1–4].

In particular, internet searches for disease-related information have shown great promise [5] when used to follow epidemics such as Dengue [6–9], Avian Influenza [10],

and Zika [11]. Launched in 2008, Google Flu Trends (GFT) [12] had already showcased the potential of online-based surveillance by predicting the timing and magnitude of human influenza activity through the aggregation of flu-related search trends. Unlike traditional surveillance methods, GFT provided near real-time reports without relying on clinical or lab data [13]. More recent studies have found strong evidence that collective online searches for health information from flu-infected individuals, can be a reliable indicator of flu activity [14–18].As the use of online search data for disease surveillance has matured, attention has shifted from whether these data are useful to how the most informative search terms should be selected. Even with recent advances in artificial intelligence, careful keyword curation remains essential for identifying the search terms that carry the most relevant information for disease prediction [19].

However, flu infection is not the sole (and perhaps not even the strongest) motivation behind flu-related online searches [20], and distinguishing between searches related to actual cases and searches performed for other reasons (e.g. fear, hype) is challenging. This is particularly true during extraordinary events when individuals are expected to have varying degrees of interest, ranging from curiosity to anxiety, to actual disease [21], resulting in high signal (infection-driven searches) and high noise (searches driven by other factors). In fact, the GFT model missed the first wave of the 2009 flu pandemic and overestimated the magnitude of the severe 2013 seasonal flu outbreak in the USA [20, 22]. Similarly, when studying the 2016 Zika epidemic, Tizzoni et al. found a poor correlation between information-seeking and disease dynamics, with media attention being an important confounding factor [23]. As a result, several researchers have become skeptical about the potential of online searches as a monitoring tool, reasonably arguing that it is too prone to overfitting and spurious correlations. Others have proposed improvements, such as including temporal dependencies through autoregressive terms (e.g., ARGO [18, 24]). These approaches refine predictions, but are often still limited by timely data availability, assume typical seasonality, with current events resembling past ones, or rely on black-box models that are difficult to interpret. Here, we argue that pandemics provide a natural experiment as they amplify not only cases, but also fears, online behaviors, media hype, etc. This high intensity can be leveraged to disentangle between behaviors, particularly online searches driven by actual disease (signal) from those driven by other factors (noise). Moreover, we hypothesize that the "true signal" identified during pandemics will be useful to nowcast seasonal disease outbreaks, as long as the disease characteristics (symptoms, progression) remains approximately the same.

The first pandemic of the 21st century was caused by the Influenza A strain (H1N1)09pdm (pH1N1 for short), which emerged in Mexico in February 2009 [25]. By June 2009, pH1N1 had spread globally, with around 30,000 confirmed cases in 74 countries. It exhibited a bi-phasic activity in most countries: a spring-summer wave and a more severe fall-winter wave [26, 27], coinciding with the common flu season in the Northern Hemisphere [28]. The pandemic was declared over in August 2010, with 18,449 laboratory-confirmed deaths, which were later revised by [29] to have been 15 times higher [30]. The second pandemic was caused by the SARS-CoV-2 virus, first identified in December 2019 in Wuhan, China. To date, COVID-19 has infected over 777 million people and killed more than 7 million worldwide [31]. While both the H1N1 and COVID-19 pandemics were global health crises triggered by respiratory viruses and taking place during the internet era, they differed significantly in virology, epidemiology, public health policy, and societal effects. Initially, both pandemics led to extensive research efforts and precise infection tracking through large-scale laboratory confirmations [32, 33]. Moreover, unlike the flu pandemic, in the case of COVID-19, testing policies varied widely between countries and over time, with laboratory testing eventually being supplanted by at-home rapid test kits. Negative results from the latter

were not systematically reported, leading to increased uncertainty in incidence estimates. Other important differences between the pandemics include the epidemiology of the various SARS-CoV-2 variants, which varied widely [34], the time it took from first infections to vaccination [35–37], duration of the pandemics, and peak dynamics, with COVID-19's spread being much less predictable, with large outbreaks occurring at irregular intervals, even during warm seasons. Additionally, SARS-CoV-2 proved to be much more infectious and severe than the pH1N1 flu strain, leading to a stronger public health response, including lockdowns, compulsory masking, and other restrictive non-pharmaceutical interventions [38].

Overall, COVID-19 raised significantly more public concern and anxiety [39, 40] and this was reflected in an increase in online activity, which became broadly used to explain and forecast COVID-19 disease dynamics [41–46]. Beyond volume, evidence shows that both real risk and risk perception shape online health information seeking [47], and that searching behaviors differ for people who feel ill versus healthy [48, 49]. Queries about institutions or public measures, by contrast, often rise with information exposure and broader public concern. Online searching is also positively associated with intentions to take preventive measures [50, 51].

Thus, we propose to learn from the variability and intensity (signal, noise, anxiety, etc.) of pandemic-related searches and ask: 1) Can these pandemic periods inform post-pandemic or seasonal disease trends? 2) Can we refine current supervised and unsupervised methods by incorporating behavioral insights?

We focus on the USA due to its high number of flu and COVID-19 cases, the availability of comprehensive data, and the occurrence of multiple COVID-19 waves. Recent reviews have emphasized that effective feature selection should identify informative predictors while reducing redundancy, thereby improving predictive performance, robustness, and model interpretability [52, 53]. However, relatively little attention has been paid to whether pandemic periods themselves can be exploited to improve feature selection. To address this gap, we propose a simple and explainable feature selection framework that leverages the high-intensity periods of pandemics as natural experiments to identify search terms with greater temporal robustness for disease nowcasting. Unlike conventional big data approaches, which primarily mitigate overfitting through statistical feature selection, our method prioritizes search terms that remain relevant across different outbreak phases, improving model interpretability and robustness. To evaluate this approach, we first describe the data sources and modelling approach, then assess its performance across influenza and COVID-19 case studies, and finally discuss its implications for the design of robust and interpretable disease nowcasting systems.

Materials and methods

Health data is considered sensitive under the EU GDPR. In this study only previously anonymized and aggregated data was used.

Data Sources

Infectious disease Data

Data for both the 2009 H1N1 flu pandemic and the COVID-19 pandemic were collected for the United States. Influenza: case numbers were obtained from the CDC's National Respiratory and Enteric Virus Surveillance System [54], covering March 2009 to August 2019, which includes the pandemic period (March 2009–August 2010) and post-pandemic period (August 2010–August 2019). The pandemic period was used

exclusively for feature selection (as described in the hierarchical clustering section below), while modeling was conducted on post-pandemic seasonal flu data. COVID-19: case numbers were collected from March 15, 2020, to May 7, 2023, from ECDC's Our World in Data [55]. This period aligns with key milestones: the declaration of COVID-19 as a global pandemic by the World Health Organization (WHO) on March 11, 2020, major outbreak waves and vaccination roll out, and the official end of the COVID-19 public health emergency in the United States on May 11, 2023. While there are already two years of cases after the official end of the pandemic, the way the data is gathered and reported changed considerably, making training models very difficult. Therefore, in the case of COVID-19 both clustering and modeling were restricted to within-pandemic data.

Search Trend data

Data extracted from GT returns the normalized, scaled and aggregated total number of searches for a particular query, in a specified geographical region and time range. All data is anonymous and it is virtually impossible to identify individual searchers. Since at the time of writing, Google did not provide an official API for Google Trends, the *pytrends* Python package was used instead. This automates requests to the same widgets shown in the public web interface (e.g., Interest Over Time) and returns the same normalized index values [56]. The analyses relied on the exported index values, not on any *pytrends*-specific transformation. Data collection was structured around the 'TrendReq' function, configured to retrieve search trends from the United States in English (using the parameters hl="en-us" for language and tz=360 for timezone). Details regarding the extraction dates can be found in the Data processing section. To verify consistency, the *pytrends* outputs were bench-marked against manual downloads from the Google Trends website for the same queries, locations, and dates (see S8–S9 Figs in the Supporting information), by calculating Spearman and Pearson correlations.

Search terms were selected to cover various aspects of pandemic and seasonal flu, and COVID-19, such as symptoms, treatments, personal care, institutions, and pandemic-specific terms. The selection process was conducted in three steps: First, a literature review was performed to identify commonly used terms, which were then supplemented with additional terms proposed by the authors. Second, these terms were queried in Google Trends, and those suggested by the pytrends 'related queries' option were added to the list. Third, terms with low search volumes, characterized by Google Trends data containing many 'zeros' interspersed with occasional high values were excluded. After this process, 42 flu-related weekly search trends and 68 COVID-19-related trends were kept. Both word lists are shown in Fig 1 panels A and E, and are provided in S1 and S2 Tables.

Media data

Flu: Mentions to 'flu' or 'influenza' were collected from 2008-08-10 to 2013-04-07. TV mentions were collected from the Vanderbilt Television News Archive [57], which includes major U.S. television networks: NBC, CBS, CNN, FOX, and MSNBC. Print media data were obtained from The New York Times API (https://developer.nytimes.com/). COVID19: Mentions of "COVID-19" from December 1, 2019, to August 31, 2023, were collected using Media Cloud [58], a platform that aggregates media coverage. The final dataset includes 249 sources, covering a diverse range of major national newspapers, online news platforms, and broadcast media. To align temporal resolution with other datasets, the raw Media Cloud data were resampled to a weekly scale, with weeks starting on Mondays. At the time of preparing

this article, Media Cloud did not provide data as far back as 2010, and could not be used to created the flu media dataset.

Data processing

Search trends data normalization

Google Trends normalizes search volume by dividing each data point by the maximum number of searches for that term within a given geographic region and time frame, resulting in all series being scaled to the same maximum value. While every search query includes a peak value of 100, not all contain a zero (i.e., if there were no weeks with search volumes below 1% of the maximum daily value). Additionally, Google Trends applies a predefined temporal resolution based on the extraction period (daily resolution for extractions covering less than 9 months, weekly if periods are between 9 months and 5 years, and monthly if longer than 5 years). To standardize the data a multi-step approach was implemented. 1) a long-period extraction was performed for each search term across the full study period. In the case of the flu, from 2005-01-01 to 2020-12-31, for COVID-19, from 2020-01-05 to 2023-05-28; 2) to capture daily variations in search frequency, month-to-month extractions were conducted; 3) the daily search volume was normalized so that the sum of all daily values within each month was 1; 4) the timeseries were combined by multiplying the normalized daily frequency by the corresponding monthly search volume; 5) daily data were aggregated into weekly values by computing the average daily volume for each week, resulting in an extended dataset with a consistent weekly resolution; 6) The entire dataset was rescaled between 0 and 100 to maintain comparability across time series. Code demonstrating this process is available in the repository under the name 'Extended Google Queries'.

Defining time windows

The modeling approach differed between the two pandemics due to data availability constraints and differences in disease seasonality. Flu: the pandemic period (defined as 2009-03-01 to 2010-08-01) was used only for cluster definition via hierarchical clustering. Modeling was conducted exclusively on post-pandemic seasonal flu data (2010-08-22 to 2019-08-11). For modeling, the different periods (P) for training and testing models were defined through visual inspection over nine years of data, ensuring they captured recurring seasonal patterns. To closely replicate real-time now-casting scenarios, six different breakpoints were selected, in the summers of 2013, 2014, 2015, 2016, 2017, and 2018 (Fig 1 D). The breakpoint was chosen to coincide specifically with the lowest number of cases so it could be used for training, and predict complete flu seasons. Exact breakpoint dates are provided in S3 Table.

COVID-19: data was extracted between 2020-03-15 and 2023-05-07. As there are several non-periodic peaks in the cases time series, there is no clear way to separate them. The choice was to use every time point as a breakpoint and train the model in 12 week sliding-windows.

Hierarchical clustering

Hierarchical clustering was applied to the (standardized) Google search terms timeseries, by computing the pairwise Euclidean distances between each term time series and using Ward's linkage method for clustering. Resulting dendrograms in Fig 1A and 3E.

Analyses were performed using Python, using the scipy.cluster.hierarchy.dendrogram function from the SciPy library [59]. The number of clusters was determined through visual inspection of the dendrogram structure, ensuring that meaningful groupings were captured. Cluster labels were assigned after clustering based on the most frequent terms

in each cluster and on their temporal behavior. They were not used as targets or inputs during model training, and they do not change how models are estimated or evaluated, they were only used to describe groups of search terms and to define the C1, C2, and C3 feature sets. This analysis was conducted separately for the H1N1 pandemic, using data from 2009-03-01 to 2010-08-01, and for COVID-19, using data from 2020-03-15 to 2023-05-07.

Modeling approach

Linear Regression

To model the relationship between search trends and infection cases, a linear regression model was implemented, of the form:

$$I_i = \beta_0 + \beta_1 \times W_1 + \beta_2 \times W_2 + ... + \beta_n \times W_n + \epsilon_i \tag{1}$$

Where I_i represents the number of infections in week i, β_0 is the intercept, β_1 to β_n are the coefficients of each search term and ϵ_i is the error. Here, W_1 to W_n denote the weekly search volumes for each selected search term, capturing fluctuations in online interest over time. These terms serve as predictor variables, allowing the model to quantify how variations in search behavior relate to changes in infection counts. The coefficients were estimated to minimize the sum of the square of the errors across all weeks. The regression was implemented in Python using sklearn.linear_model.LinearRegression [60] with default parameters. The input features consisted of Google search terms selected as previously described, while the target variable was the number of infection cases per week. To evaluate the contribution of different search term cluster dynamics, separate models were run using all search terms (All) as well as specific clusters: Cluster 1 (C1), Cluster 2 (C2), and Cluster 3 (C3). Each cluster model followed the same training and evaluation procedure to ensure comparability.

Lagged cases and search terms were introduced to the COVID-19 models to capture the delay between online search behavior and infection trends. As the pandemic was still ongoing, unlike the 2009 flu pandemic, it was necessary to incorporate temporal dependencies to improve predictive accuracy. To account for this, four weeks of lagged cases were included, and each search term was lagged by 0, 1, 2, or 3 weeks, following an approach similar to the implementation of the ARGO model [24]. The optimal lag for each search term was determined independently for each model as a hyperparameter, selecting the lag that best predicted cases in a univariate regression.

Random Forest

A Random Forest regression model was implemented to capture potential nonlinear relationships between search trends and infection cases. The model followed the same training and evaluation procedures as linear regression and LASSO. Training was performed using sklearn.ensemble.RandomForestRegressor [61], with hyperparameters—number of estimators, maximum features, and maximum depth—optimized through cross-validation using GridSearchCV. The tested values for these parameters were selected from the ranges [10, 20, 50, 100, 200, 500, 1000] for the number of estimators, [0.6, 0.8, "auto", "sqrt"] for maximum features, and [2, 4, 5, 6] for maximum depth.

To account for variability in model performance, multiple random seeds were used. For the 2009 Flu pandemic, each model was trained using 100 different random seeds, ensuring robust evaluation across different initial conditions. For COVID-19, given that

142 separate models were already running, 10 random seeds per model were used, balancing computational feasibility with performance reliability.

Evaluation metrics

Bias-variance trade-off

To estimate bias and variance, the mlxtend Python package [62] and the evaluate.bias_variance_decomp algorithm were used. This method decomposes the total error into bias and variance by performing bootstrapped resampling iterations over the training data. For both random forest and linear regression flu models, 200 resamples were performed; for Covid-19 random forest, only 10 were used because it would not be feasible to run hundreds of samples over 142 models. Bias was defined as the mean error of the models for each week, while variance was computed as the mean variance of the model predictions for each week. This decomposition allowed for a detailed assessment of the trade-off between underfitting and overfitting across different modeling approaches.

LASSO regression

Implemented as an alternative to linear regression to perform feature selection while minimizing multicollinearity. The model followed the same training and evaluation procedures as linear regression. Unlike standard linear regression, LASSO incorporates L1 and L2 regularization, shrinking less informative coefficients to zero. Hyperparameters were optimized using cross-validation with ElasticNetCV (in all cases, the regularization that had the best performance was L1), and the selected coefficients were analyzed to determine the most predictive search terms across different time frames.

Train-Test split sensitivity analysis

To assess the impact of train-test split selection on model performance, the variability in R^2 scores across different rolling window periods was analyzed (see Modeling Approach). This analysis was conducted only for COVID-19 using Linear Regression, as it provides an interpretable baseline to isolate the effects of train-test split choices without introducing additional complexity from nonlinear models. Results were compared for the "All" and C1 search term sets, evaluating whether certain train-test split points systematically led to better or worse performance. R^2 scores were computed across 142 overlapping models to quantify this variability.

Variant-stratified evaluation and longitudinal feature-importance tests

For the variant-stratified analyses, the COVID-19 timeline was divided into seven non-overlapping phases using inclusive start and end dates to tag each week: Pre-Alpha (March 15, 2020 to December 13, 2020), Alpha (December 14, 2020 to June 20, 2021), Delta (June 21, 2021 to December 12, 2021), Omicron BA.1 (December 13, 2021 to March 20, 2022), Omicron BA.2 (March 21, 2022 to June 19, 2022), Omicron BA.5 (June 20, 2022 to October 16, 2022), and Omicron XBB+ (October 17, 2022 to May 7, 2023) [63]. Phase-specific performance metrics (RMSE and R^2) were computed using only the weekly predictions within each phase and also calculated a four-week rolling RMSE to capture short-term fluctuations. To examine whether individual search terms changed in predictive relevance, weekly Random Forest feature importances from the "ALL" model were used, and plotted in a term-by-week heatmap with vertical lines marking the variant boundaries. For each term, it was then tested whether importance

decreased after January 2022 using a one-tailed Mann–Whitney U test (pre-2022 vs post-2022), applying Benjamini–Hochberg FDR correction [64] at 0.05. Terms with a significant decline are marked with an asterisk in the heatmap (S14 Fig). This procedure quantifies when specific search behaviors (e.g., "loss of smell") lost predictive value as public perception and testing practices evolved.

Random feature selection comparisons

To test whether the C1 feature set captures meaningful behavioral patterns, its performance was compared against randomly selected feature sets of the same size and evaluated C1's percentile rank within the resulting distribution of random models.

For each flu season, sets of 9 search terms were randomly sampled (matching the number of terms in C1) and models were trained on them. This process was repeated 1000 times per season to generate a distribution of R^2 scores. The performance of C1 was then compared with the average performance of these random models and C1's percentile rank was computed within the distribution to determine the proportion of random selections with lower performance. A percentile rank of 100% indicates that C1 outperformed all random selections, while lower ranks suggest that some random sets performed equally well or better.

All models were evaluated using R^2 and bias-variance decomposition to assess predictive performance and generalizability.

Correlation analysis

Correlation analyses were conducted across multiple time periods to evaluate the relationship between search trends, confirmed cases, and media coverage. While the primary feature selection step was performed using data from the pandemic periods (March 2009 to August 2010 for H1N1, and March 2020 to May 2023 for COVID-19), correlation analyses were also extended to post-pandemic flu seasons to assess the stability of these relationships over time.

For each period, Pearson correlation coefficients were computed between the weekly search volumes of each term and the number of confirmed cases. For the flu pandemic, correlations were calculated separately for each predefined training period (P1–P6) to determine how search term relevance varied across different flu seasons. This was implemented in Python using the `scipy.stats.pearsonr` function. Additionally, correlation analyses were performed between search trends and weekly media mentions of flu and COVID-19.

To evaluate the impact of correlation-based feature selection, two approaches were tested: selecting the 10% most correlated search terms during the pandemic period, filtered for statistical significance ($p < 0.05$), and identifying the minimum number of highly correlated search terms per flu season required to achieve maximum predictive performance.

Results

Online disease-related searches show distinct patterns

The primary objective of this study was to develop a simple, cost-effective, and reliable now-casting system that balances accuracy and interpretability. Following previous research [6–9], we used Google search volumes as a proxy for epidemic activity in the population. As a first step, we examined whether seemingly similar search terms

exhibited distinct temporal patterns, as differences in search dynamics may reflect variations in underlying motivations.

To explore this, we analyzed the temporal behavior of multiple related search terms during the 2009 H1N1 and COVID-19 pandemics in the USA. Pandemic periods were selected as they amplify both actual infection-driven searches and fear or other-driven interests, providing a clearer distinction between signal (searches linked to disease incidence) and noise (searches influenced by external factors). Applying hierarchical clustering to these terms identified three distinct groups in both pandemics (Fig 1A and E). Fig 1B and 1F) illustrate the average search volume of all search terms ("All"), with gray shading highlighting periods of higher variance in search dynamics. Cluster labels were assigned after clustering from the most frequent terms and their temporal behavior and are used only to aid interpretation at the population level.

For H1N1, Cluster 1 (C1, orange) exhibited a sharp peak in early 2009, followed by a broader secondary increase. Cluster 2 (C2, purple), showed a single peak in early 2009 with little subsequent activity, and Cluster 3 (C3, green), displayed a noisier pattern, with the largest peak occurring in late 2009 (Fig 1B).

For COVID-19, C1 (orange) initially fluctuated before reaching a sharp peak in December–January 2021, coinciding with the highest number of cases (Fig 1G). C2 (purple), exhibited an early pandemic peak followed by irregular spikes, and C3 (green) showed multiple peaks in early 2020, late 2021, and winter 2022, with a smoother trajectory overall (Fig 1F).

Since Google Trends extrapolates search volumes from sampled data and periodically updates its outputs, we performed two technical controls. First, we tested whether the observed clusters were robust to changes in data retrieval timing. Across different collection periods, including those spanning multiple years, the clustering structure remained stable (S1 Fig), indicating that the observed patterns likely reflected real dynamics. Second, we further tested whether the results were an artifact of the data retrieval tool (*pytrends*), by comparing these time series with the official Google Trends CSV exports (two queries per cluster). For all tested terms, correlations were very high (all Pearson and Spearman coefficients > 0.94, Tables S7 (flu) and S8 (COVID)), and temporal profiles were similar (S8 and S9 Figs). These controls confirm the reliability of the automated pipeline in producing consistent estimates of search interest over time, supporting its use for epidemiological applications that require periodic data updates. Thus, despite largely following epidemic waves (S7 Fig in the Supporting information), different search terms exhibit distinct temporal signatures.

For both pandemics, search terms were automatically grouped into three clusters , for each disease, with the COVID-19 clusters being less well-defined than H1N1's, as shown by higher variance in centroid curves (Fig 1F) and by shorter branches in the hierarchical clustering (Fig 1E). This suggests that different search motivations contribute to these dynamics. If so, some clusters may be more associated with actual disease and those should more predictive of disease trends than other clusters (and even than individual search-terms). Still, in what follows, cluster labels are used to summarize group behavior, and should not be read as claims about individual motivations.

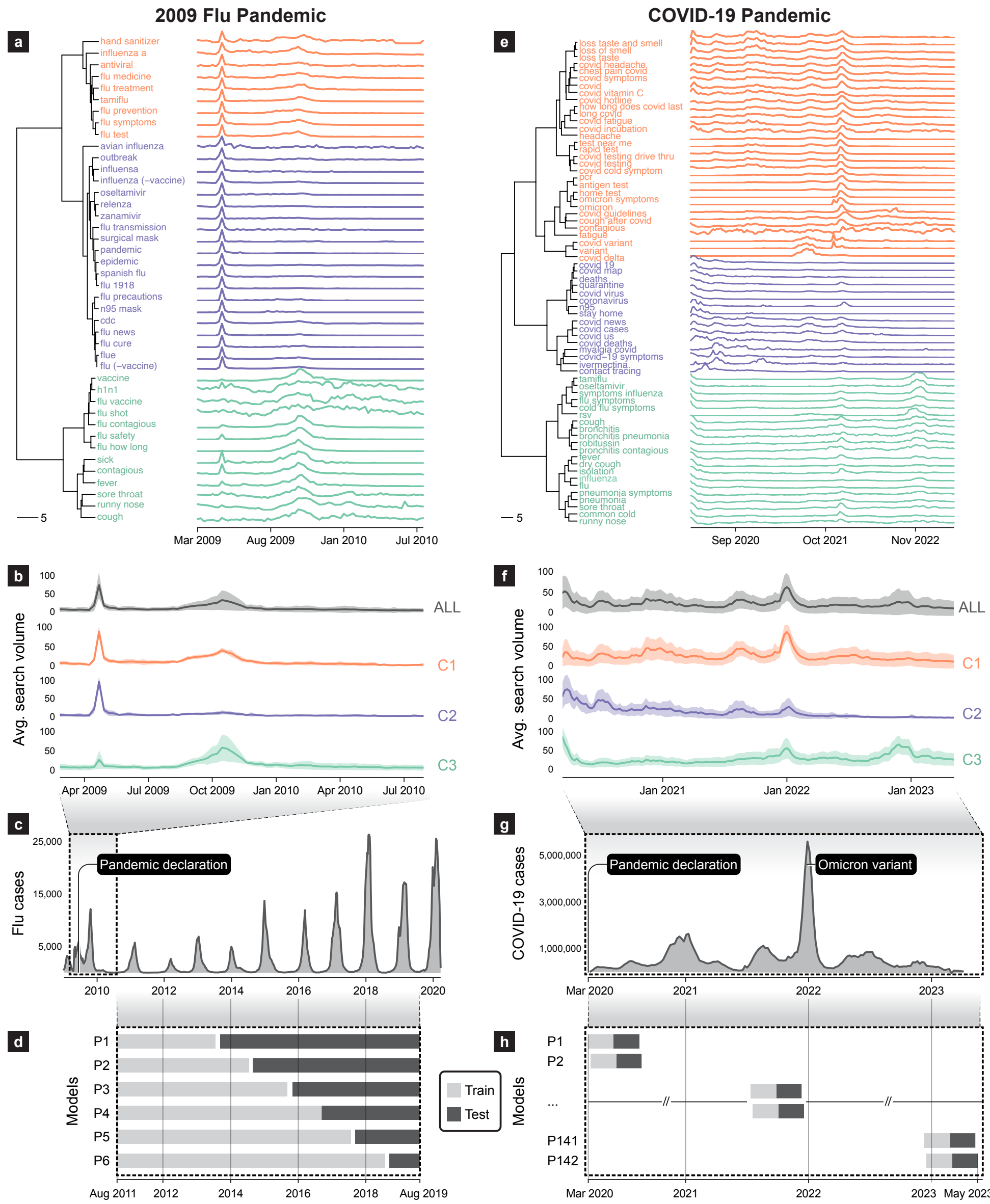

Fig 1. Search term clustering, infection trends, and modeling approach for the 2009 H1N1 and COVID-19 pandemics. (a, e) Hierarchical clustering of search terms into three clusters (C1, C2, C3) based on temporal search volume patterns. (b, f) Cluster centroids with variance indicated by shaded areas. (c, g) Reported flu (c) and COVID-19 (g) cases over time, with key pandemic milestones highlighted. Shaded areas indicate the timeframe used for clustering. (d, h) Training and testing periods for now-casting models. For H1N1 (d), post-pandemic flu seasons were used for train and test splits. For COVID-19 (h), a rolling window approach adapted models across pandemic phases.

Pandemic periods as a natural experiment for improving seasonal flu now-casting

We hypothesized that "true signal" search terms identified during pandemics remain predictive beyond them and could enhance seasonal now-casting, improving our ability

to track disease dynamics outside of crisis conditions.

To test this, we evaluated whether search terms identified during the 2009 flu pandemic could accurately predict seasonal flu trends in subsequent years. We compared models trained using all search terms with those trained using terms based on single clusters (Fig 1A). These models were validated across six seasonal flu periods (P1–P6), of different intensities (Fig 2C, D). Predictive performance was assessed using the coefficient of determination (R^2) for Linear Regression and Random Forest models (Fig 2A and B).

Overall, Linear Regression outperformed Random Forest in five out of six test cases, particularly when the validation period included severe flu seasons (i.e., seasons with high peaks of reported cases), with 2018 being the most glaring example. This can be explained by the large differences in amplitude between training and testing data. Random forests perform regression tasks by building trees where each branch partitions the dependent variable arbitrarily [65]. Since each tree will have its own cutoffs, this makes the models very good at tackling non-linear problems. However, they cannot learn any partition beyond the range present in the training data. If the features point to a very high (low) value, then the trained model will predict the highest (lowest) value present in the training data. If so, the expectation is that Random Forest will perform the poorest when the highest or lowest values were not present in the training data. Indeed, this is what S3 Fig shows, and has been observed by others (e.g. [66, 67]): the only exception to the Random Forest under-performance was P6, for which the test period did not include the unusually intense 2018 flu outbreak. Model performance and full comparisons between observed and predicted cases for each period are provided in S4 Table and S2–S4 Figs in the Supporting information.

Critically, C1 consistently achieved the highest R^2 values across models, outperforming other feature sets in 4 out of 6 seasonal flu periods (Fig 2 A). The two exceptions were P2, where C1 and "All" performed equally well, and P6, where "All" terms performed better under linear regression (see S4 Table in the Supporting information). Unlike models trained on all search terms ("All", gray), which showed greater variability in accuracy across flu seasons, C1 produced more stable performance for seasonal flu cases. The performance of C2 (purple) was intermediate, with R^2 values generally lower than C1 but higher than C3 (green).

These results suggest that search term selection based on clustering during a pandemic period can enhance seasonal flu now-casting by reducing noise and improving performance stability. In contrast, using all available search terms introduces additional variance, likely capturing both relevant and irrelevant signals, which may lead to less robust seasonal predictions. Still, before the COVID-19 pandemic, flu outbreaks had clear seasonality and similar symptoms, and these results may not hold in the case of less repetitive contexts.

Applying flu-based feature selection to improve COVID-19 now-casting

While flu-related searches have historically shown consistency between seasonal and pandemic contexts, COVID-19 presented unique challenges that complicate now-casting efforts. These include: (1) the absence of a pre-existing surveillance infrastructure, such as the sentinel doctor networks available for flu; (2) uncertainty regarding whether COVID-19 will become a seasonal disease, as its outbreak periodicity remains unclear [68, 69]; (3) rapid and evolving changes in reporting practices, testing availability, and public health responses throughout the pandemic; and (4) the lack of a long historical dataset for model training. Therefore, the described relationship between pandemic-period search terms and disease incidence for the flu may not be observed in

the case of COVID-19.

Despite these challenges, we tested whether the feature selection approach applied to flu search terms could also improve COVID-19 now-casting (see Methods). We collected official case counts from March 2020 to May 2023, marking the beginning and end of the pandemic period. Instead of using predefined seasons (or waves) to train and test models, we applied a rolling window approach with 142 train/test splits (Fig 1G and H), including all possible break points.

We then assessed the accuracy of Linear Regression and Random Forest models trained using all search terms versus the clusters identified through hierarchical clustering (Fig 1E and F). Unlike the flu models, where we used a standard linear regression approach, for COVID-19 we implemented ARGO (AutoRegression with GOogle search data) [24], which incorporates search volume and cases from previous weeks as additional predictors. In this case, the Linear Regression models also outperformed the Random Forest, but likely for different reasons than in the case of the flu. Here, both models are equally poor at forecasting the largest peak (S5–S7 Figs in the Supporting information), but the Random Forest predictions are generally worse, while the Linear Regression fails less consistently. One possible explanation is the ARGO model using past predictions as a feature, while the Random Forest does not include such auto-correlation inputs.

The results (Fig 2C and S5–S6 Figs in the Supporting information) confirm that, as in the flu analysis, C1 search terms provided better predictions than using all search terms, C2, or C3 alone. These findings further support the idea that semi-manual feature selection can enhance the performance of now-casting models, particularly in distinguishing terms that better reflect disease dynamics.

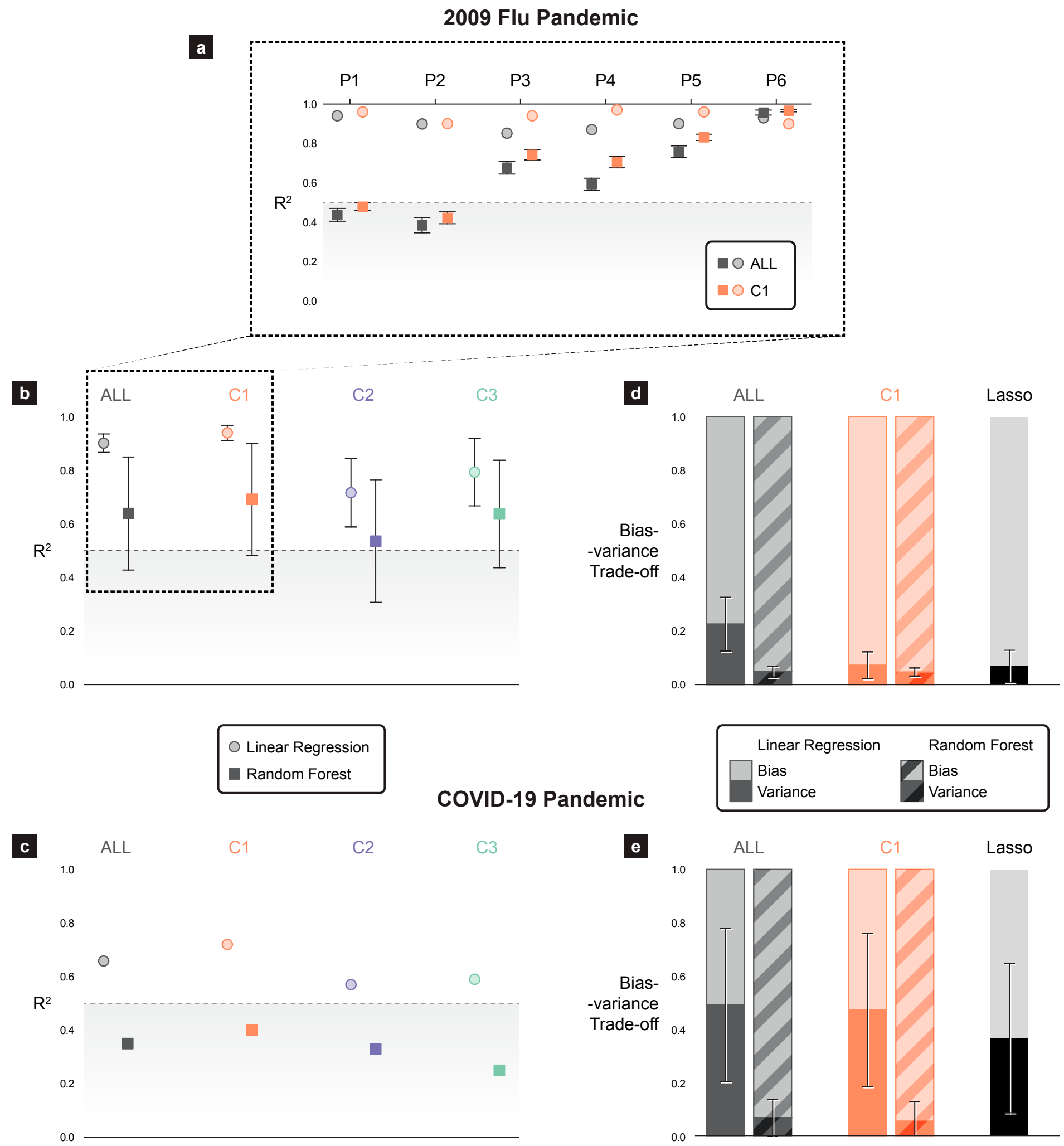

Fig 2. Comparison of now-casting model performance and bias-variance trade-off. Predictive performance of Linear Regression (circles) and Random Forest (squares) using different search-term clusters (All, C1, C2, C3) during the 2009 Flu and COVID-19 pandemics. Panels a), b), and d) correspond to flu, while c) and e) to COVID-19. For flu, error bars represent standard deviation across multiple seasons (P1–P6), reflecting model variability. Panel a) highlights C1's superior performance over "All". For COVID-19, standard deviations are not shown due to the rolling window approach, which results in overlapping training data and non-independent R^2 estimates. Instead, panel c) presents the R^2 of the mean prediction across models. Panels d) and e) illustrate the bias-variance trade-off. Random Forest has lower variance but slightly higher bias than Linear Regression. In flu (d), C1 reduces variance compared to "All", suggesting that selecting fewer, more relevant terms improves stability. In COVID-19 (e), bias remains high due to the pandemic's unpredictability. While here C1 also lowers variance relative to "All", this effect is weaker than in flu, where seasonal patterns allow for more consistent predictions. LASSO shows high bias and low variance.

Overfitting reduction, generalization, and predictive reliability in feature selection

The differences in performance observed between models using C1 search terms and those using all available terms are likely driven by overfitting: when including too many predictors, models learn patterns that do not generalize beyond the training data and exhibit high variance across different train/test splits [70, 71]. In contrast, using too few or poorly chosen features can lead to underfitting, where models miss meaningful relationships and display high bias. Managing the trade-off between reducing variance and introducing excessive bias, is essential to building robust models.To test this, we compared bias-variance trade-off of models trained on the terms from the C1 cluster versus those trained using all search terms (Fig 2D–E and S5–S6 Tables in the Supporting information).

Across both pandemics, bias tended to dominate variance, especially in the Linear Regression models, which is expected given their simplicity. Using all search terms increased variance compared to C1 for the flu, reinforcing the idea that removing less informative predictors stabilizes performance and suggesting that reducing feature count does not compromise performance but instead provides a more curated and stable feature set. As expected, Random Forest models consistently showed lower variance across conditions, regardless of the feature group used.

As choosing the right set of features is critical to balancing model complexity and predictive reliability, we further evaluated four alternative selection strategies (LASSO regression, correlation-based filtering, cluster size, and train-test split variability), also repeating the analysis by pandemic phase - temporal selection).

LASSO applies regularization by shrinking some coefficients to zero (L1), effectively performing feature selection by removing redundant terms. This typically reduces variance but can increase bias (Fig 2D and E). In both the flu and COVID-19 analyses, LASSO achieved performance levels comparable to C1 (see S9 Table in the Supporting information). However, most likely because LASSO selects terms based purely on statistical optimization, it led to inconsistent feature selection across the different flu seasons, (S8 Table). This variability reduces model interpretability, making it difficult to identify search terms that remain predictive over time.

LASSO regularization cannot be applied to the COVID-19 models in a straightforward way. Since we average the results of 142 models, and each model has its own ideal set of weights, it is unlikely any term would be removed from all models.

Second, and as C1 terms appeared to track infection trends well, we tested whether correlation-based selection could achieve similar results. The rationale is that similar results could have been achieved by simply identifying the search terms more correlated with the number of cases. We compared models using C1 and "All" pandemic-period terms to two correlation-based methods: (1) selecting the top 10% of search terms most correlated with flu cases during the pandemic period ($p < 0.05$), and (2) selecting the minimal number of terms per season needed to reach peak model performance during training.

The results show that selecting search terms based on correlation alone can yield strong predictive performance, at times exceeding C1 (see S11–S12 Tables in the Supporting information). This is consistent with recent work on dengue forecasting using Google Trends, where parsimonious search-term sets selected through statistical associations outperformed larger collections of search terms [72]. However, the effectiveness of this approach varied depending on whether selection was based on the pandemic period or adjusted for each period individually. When selecting the most correlated search terms during the pandemic period (five search terms), predictions remained strong but did not outperform seasonal selection or C1. For the seasonal selection of terms that achieve the peak model performance during training, the number

of predictors varied widely, from 34 search terms in one of the seasons to just one search term (“influenza a”) in others.

This indicates that selecting features by correlation is highly sensitive to period-specific variation, suggesting that while pandemic periods help identify relevant search behaviors that are long-lasting, filtering by correlation alone does not ensure the best results in terms of performance or stability across different seasons.

Third, a simple explanation is that C1 has better results just because it includes fewer terms and this lowers model complexity. This is unlikely, as C1’s size varies (smaller cluster in the case of the flu, largest in the case of COVID-19) yet systematically outperforms models trained with the other clusters (C2 and C3). Still, and to ensure that better predictive performance attributed to C1 was not simply due to the number of selected predictors, we compared performance, bias and variance of the C1 feature set against random selections of the same size. As S11 Table shows, across all flu seasons, C1 consistently outperformed models trained on random sets of nine terms. While some random selections performed well, their average R^2 was lower in every season (0.56–0.81 versus 0.90 - 0.97). Similarly, random models exhibited higher bias, indicating systematic underfitting, while C1 maintained a better bias / variance trade-off. In summary, C1 outperformed over 90% of random selections in 5 out of 6 seasons. Even in its weakest period, P6, it still performed better than 60% of random models, suggesting that any alternative high-performing sets emerge only by chance and lack interpretability. No random model using nine features matched or exceeded C1 across all seasons. We decided not to replicate the random feature control for COVID-19, as the C1 cluster in this case already included the largest number of terms, making it unlikely that its superior performance is due to model simplicity. Moreover, the flu analysis had already shown that random subsets of equal size rarely outperform behaviorally informed features, reducing the added value of repeating this analysis for COVID-19.

Fourth, we examined how train-test split variability affected model performance for COVID-19, focusing only on models trained with either the C1 feature set or all search terms. Unlike flu, where predefined seasonal periods provide a natural breakpoint structure, COVID-19 lacked clear seasonality, making model accuracy highly dependent on the specific division between training and testing data. In fact, performance varied substantially across different split points: some yielded strong predictions (R^2 ¿ 0.6), while others produced near-zero or even negative R^2 values (S6 Fig). Both C1 and “All” models followed similar trends, although the ”All” models exhibited a slightly wider spread in performance, suggesting that including more search terms can enhance signal detection in some cases but also increase instability. We would like to emphasize this finding: we are aware that previous models have reached higher performance and we can replicate those results here; however, this high performance is too dependent on arbitrary choices and cannot be achieved systematically.

Finally, to further examine the temporal stability of model performance, we stratified the analysis by dominant COVID-19 variant phases (see S13–S14 Figs and S14 Table in the Supporting information). An increase in RMSE was observed during the Omicron BA.1 transition (winter 2021–2022), indicating a temporary decline in predictive performance coinciding with shifts in testing behavior and symptom perception. In parallel, a longitudinal evaluation of feature importance from the Random Forest models showed that symptom-specific terms such as *loss of smell* and *loss taste* significantly declined in predictive relevance after early 2022, whereas more general COVID-related queries (e.g., *covid cases*, *testing*) remained relatively stable. These findings suggest that changes in strain dominance coincide with changes in search patterns, emphasizing the importance of continuously incorporating behavioral dynamics into nowcasting frameworks.

Overall, our findings show that hierarchical clustering offers a practical and robust approach to feature selection. While LASSO and correlation-based methods may perform well locally, they produce inconsistent feature sets across time. C1, in contrast, offers a reliable balance between accuracy and stability. These are important qualities for models designed to operate in real-time surveillance contexts.

An *a posteriori* analysis of flu-related search terms shows that C1 mainly includes queries tied to illness perception and treatment-seeking, such as "flu symptoms," "flu prevention," and "flu treatment." While similar terms appear in other clusters (e.g., "flu precautions" in C2, "flu safety" in C3), C1 is more consistently linked to self-diagnosis and prevention, suggesting that these searches are consistent with queries triggered by symptoms. For COVID-19, C1 mostly captures general symptoms, C2 includes societal-level terms, and C3 reflects testing and public health guidance. However, these interpretations are ad hoc: an *a priori* assessment would not necessarily anticipate the observed cluster structure and these clusters cannot be used to infer individual intent. Still, if certain search terms are more likely driven by personal illness and others by media or external cues, the clusters may reflect different underlying "real-world" behaviors.

To test this, we examined how each cluster correlates with confirmed case numbers and media mentions. The rationale being that if a cluster strongly correlates with case numbers, its terms are more likely to track actual disease burden, whereas a high correlation with media mentions suggests external information is shaping search behavior.

We collected media mentions of flu and COVID-19 over the same periods as case numbers and search trends (see Methods) and calculated their correlation with each search term cluster. Fig 3A and B shows that C1 exhibits the highest correlation with case numbers across both pandemics, whereas C2 is more strongly associated with media coverage. This pattern is further illustrated in S11 Fig in the Supporting information, where C1 closely follows case fluctuations while C2 aligns more with media mentions.

We previously showed that C1 offers greater stability in search term selection compared to using correlated terms alone (S11 Table).

Still, we identified the individual search terms more closely correlated with cases and media attention during the pandemic period, regardless of cluster, and examined their effect on predictive accuracy (Fig 3C and D). The left panel shows R^2 values for a linear regression model, adding search terms from most to least correlated with cases (left to right). The right panel shows R^2 values when adding search terms from least to most correlated with media coverage (right to left). Ranked search terms and R^2 cumulative values are provided in S12 Fig in the Supporting information.

Across both pandemics, model accuracy improves quickly when adding case-correlated terms but soon plateaus or declines, indicating that only a subset of terms contributes meaningfully to prediction, while additional terms introduce noise (likely due to an increase in variance as explained above). A similar pattern emerges for features correlated with media mentions (Fig 3C and D, right panel), where performance initially improves but quickly stabilizes or worsens as search terms more correlated with media are added, indicating that searches driven by media reduce model performance rather than improve it.

Looking at the order in which terms are added, we find that C3 terms (green) often appear early when ranked by case correlation, reflecting their link to general disease activity. However, these broad symptom searches (e.g., "fever," "sore throat," "runny nose") offer limited predictive value on their own. They provide a baseline signal but do not drive peak performance without the inclusion of more targeted C1 terms, which appear to be more outbreak-specific. This trend is especially evident during the flu pandemic (top panels, P1 to P6), but also holds for COVID-19, where peak accuracy is

only achieved once C1 terms are incorporated. Additionally, these high levels of performance are reached with few or no C2 terms (purple), which are more closely associated with media coverage. This suggests that the clustering approach seems to effectively distinguish between behaviorally distinct search patterns, those driven by disease experience and those driven by external information, while filtering out less informative signals.

Discussion

This study aimed to develop a cost-effective and interpretable now-casting model using online search data. By applying hierarchical clustering to Google Trends during two pandemic periods (H1N1 and COVID-19), we identified consistent groups of search terms with distinct temporal dynamics. These clusters revealed that even seemingly similar search terms may be driven by different underlying motivations — some aligned with disease incidence, others with media influence or general interest. We present evidence that one of these clusters systematically outperforms more common purely statistical approaches, not only in terms of accuracy but also in terms of stability.

These findings contribute to a growing body of work that seeks to leverage digital traces, particularly search data, for real-time disease surveillance, while tackling two central challenges: 1) distinguishing meaningful signals from behavioral or media-driven noise, and 2) model overfitting and performance instability.

Our results demonstrate that behaviorally informed feature selection, especially when guided by temporal clustering, offers a promising path for improving the reliability and generalizability of now-casting models, reinforcing the key empirical lesson that more data is not always better. In fact, including all available features can introduce variance that overwhelms the true signal. Instead, our approach prioritizes coherence, identifying subsets of search terms that behave consistently during outbreaks (clusters) and remain predictive beyond crisis contexts. This aligns with recent calls for more interpretable, behavior-aware modeling strategies in digital health surveillance [73]. Importantly, we show that pandemic periods, often treated as outliers in epidemiological modeling, can function as natural experiments for feature selection. These periods amplify both disease-related and attention-related search activity, making it easier to differentiate terms that reflect disease from those driven by news cycles or societal anxiety. That these pandemic-identified terms (particularly those in C1) continue to outperform others across seasonal flu periods suggests they capture underlying behavioral regularities that are robust across time. This finding stands in contrast to purely statistical feature selection methods, such as correlation filtering or LASSO regression, which may perform well within narrow windows but often yield unstable and inconsistent results across datasets. Consistent with this view, the variant-stratified analysis highlights that model performance can shift with behavior and symptom frequency (e.g., declining value of loss of smell after Omicron). This also reinforces that feature selection alone does not guarantee temporal robustness; as search behaviour evolves, maintaining temporal robustness will require approaches that monitor changes in search patterns (e.g., feature drift) and rely on domain expertise for feature interpretation and validation.

In summary, this study offers a practical middle ground between high-dimensional, black-box models and rigid feature engineering. This is consistent with recent perspectives arguing that, in epidemiology, machine learning methods should be selected according to the question being addressed rather than solely on predictive performance [74]. Clustering based on temporal dynamics reduces dimensionality while preserving interpretability, a crucial balance in public health settings where decisions must be explainable and traceable. While both model types benefited, the advantages were especially clear for Linear Regression, a model class whose transparency makes it

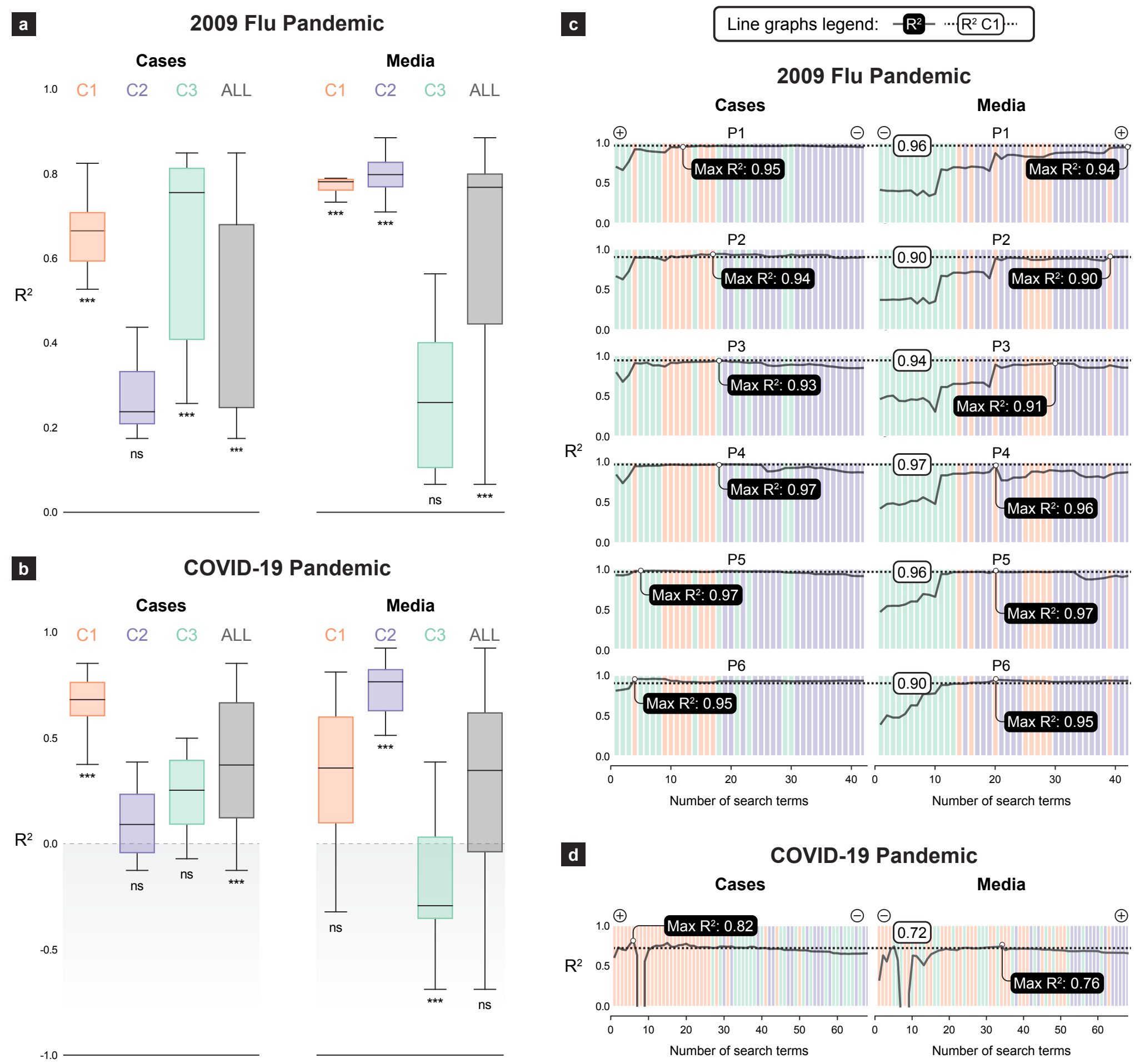

Fig 3. Correlation between search terms, infection cases, and media mentions. a) and b) show the Pearson correlation between each cluster and infection cases (left) and media mentions (right) during the pandemic period for both the flu (top) and COVID-19 (bottom). In contrast, c) and d) present the Linear Regression model's R^2 performance. The asterisks represent the p-value of a t-test with the null hypothesis that mean correlation is zero (*$0.01 < p < 0.05$; **$0.001 < p < 0.01$; ***$p < 0.0001$; ns $p > 0.05$). In a) and b), correlations can be both positive and negative, indicating whether search volumes increase or decrease with cases or media mentions. In c) and d), however, only the absolute correlation values are considered, as predictive power depends on correlation strength rather than direction. Also, c) and d) illustrates the cumulative contribution of individual search terms to the model's performance. Terms are added sequentially from the most to the least correlated with infection cases (left to right, in *Cases* panels) and from the least to the most correlated with media mentions (left to right, in *Media* panels). The black line represents the Linear Regression R^2, showing how predictive performance evolves as new terms are added, while the dashed line shows the R^2 obtained for C1 as a reference. We focus on the Linear Regression model here as it provides a more interpretable baseline for assessing the contribution of different feature sets. The colors of the vertical bars correspond to the clusters each term belongs to, allowing a comparison of cluster contributions.

particularly suitable for deployment in policy contexts. Importantly, as digital data grows in volume and heterogeneity, the risk of overfitting grows in parallel: prioritizing stable, interpretable features becomes increasingly relevant, particularly for emerging diseases with limited training data. By linking clusters to real-world signals such as case counts and media coverage, this approach supports more trustworthy, actionable machine learning in public health. Nevertheless, this method is not without limitations and we list five. First, while feature curation reduced variance and improved prediction for flu, the gains were more modest for COVID-19. This reflects a broader issue in digital surveillance: model performance is heavily dependent on the stability of underlying behaviors. In contexts with shifting testing protocols, public awareness, or policy environments, as seen in COVID-19, the mapping between search behavior and disease incidence becomes less predictable. This underscores the need for adaptive models that can recalibrate as behavioral and epidemiological baselines evolve (e.g., large scale vaccination, social distancing). Second, our clustering methodology relies on distinct temporal patterns (e.g., clear peaks), which may not exist for all outbreaks. In such cases, complementary approaches that incorporate additional contextual data, such as mobility patterns or syndromic surveillance, may enhance predictive performance. Third, while clustering reduces the inclusion of noisy predictors, it does not inherently infer causality. Some search terms may correlate with disease trends due to external factors rather than direct epidemiological relevance, as we saw with media mentions. Careful validation against traditional surveillance data remains essential to ensure reliability. Fourth, this study focused on a large country with broad access to online tools. Digital indicators may behave differently at finer spatial resolutions, and factors such as internet access, digital behavior, and public health communication strategies can influence the reliability of using search-terms in different geographic contexts. However, the inexpensive nature and scalability of this method may make it particularly useful in low-resource settings where traditional disease surveillance is limited [73]. Indeed, if deployed responsibly, this method also offers promise to help reduce health inequities by providing rapid insights. Curated search terms, if adjusted for local behaviors and disparities in digital access, can improve disease surveillance without reinforcing existing digital divides. Future research might assess the effectiveness of this approach across diverse geographic and socio-economic contexts. Finally, for public health use, analysis must remain consistent and reproducible. A method that relies on private platform data, as is the case of Google Trends and its API, risks interruptions or instability from changing access paths, shifting policies, quota limits, pricing, and even basic availability. Although interface sampling can introduce minor run-to-run variation, it can minimize some of these issues. In this work, repeated exports show that comparative results remain stable (see S8–S9 Figs in the Supporting information), and preference should be given to pipelines resilient to platform variability. Still, they do not solve the fundamental problem of relying on non-public data. Overall, future work should explore the integration of additional data sources, such as mobility data, syndromic surveillance, and social media signals, to enhance robustness and generalizability.

In sum, this study demonstrates that thoughtful, behaviorally grounded feature selection can improve the robustness, stability, and transparency of digital now-casting systems, and expanding this framework to real-time monitoring could further support public health decision-making, for both seasonal and emerging infectious diseases.

Supporting information

S1 Fig. Stability of clustering and centroid patterns across independent Google Trends data extractions.

S2 Fig. Predicted vs. observed cases for the 2009 Flu pandemic (Linear Regression).

S3 Fig. Predicted vs. observed cases for the 2009 Flu pandemic (Random Forest).

S4 Fig. Residual distribution for the flu predictions.

S5 Fig. Predicted vs. observed cases for the COVID-19 pandemic (Linear Regression).

S6 Fig. Predicted vs. observed cases for the COVID-19 pandemic (Random Forest).

S7 Fig. Residual distribution for the COVID-19 predictions.

S8 Fig. Flu reproducibility: Pytrends versus official Google Trends exports.

S9 Fig. COVID-19 reproducibility: Pytrends versus official Google Trends exports.

S10 Fig. Distribution of R^2 across 142 rolling train–test splits.

S11 Fig. Media mentions, cases, and search-term clusters over time (Flu and COVID-19).

S12 Fig. Ranking of search terms by correlation with cases and media mentions.

S13 Fig. Temporal instability across COVID-19 variants.

S14 Fig. Temporal evolution of Random Forest feature importance across variant phases.

S1 Table. Search term clusters for the Flu pandemic.

S2 Table. Search term clusters for the COVID-19 pandemic.

S3 Table. Training and test period splits for seasonal prediction using Flu-period terms.

S4 Table. R^2 values for Linear Regression and Random Forest (Flu and COVID-19).

S5 Table. Bias–Variance trade-off for Flu.

S6 Table. Bias–Variance trade-off for COVID-19.

S7 Table. Reproducibility of Google Trends extraction for Flu (pytrends vs. official exports).

S8 Table. Reproducibility of Google Trends extraction for COVID-19 (pytrends vs. official exports).

S9 Table. LASSO Regression performance across periods (P1–P6).

S10 Table. LASSO weights per search term across periods (clustered by C1–C3).

S11 Table. Random feature selection (9 features) vs. C1-only across seasons.

S12 Table. Performance when selecting terms by correlation with cases (pandemic period).

S13 Table. Performance when selecting terms by seasonal correlation (P1–P6).

S14 Table. Mean RMSE and R^2 by dominant COVID-19 variant phase (Linear Regression models).

Acknowledgments

We thank the members of the Social Physics and Complexity (SPAC) group at LIP for their comments and critical reading of the manuscript.

References

1. Hay SI, George DB, Moyes CL, Brownstein JS. Big data opportunities for global infectious disease surveillance. PLoS med. 2013;10(4):e1001413.

2. Ferretti L, Wymant C, Kendall M, Zhao L, Nurtay A, Abeler-Dörner L, et al. Quantifying SARS-CoV-2 transmission suggests epidemic control with digital contact tracing. Science. 2020;368(6491).

3. Salathe M, Bengtsson L, Bodnar TJ, Brewer DD, Brownstein JS, Buckee C, et al. Digital epidemiology. PLoS Comput Biol. 2012;8(7):e1002616.

4. Du H, Zhao Y, Zhao J, Xu S, Lin X, Chen Y, et al. Advancing real-time infectious disease forecasting using large language models. Nature Computational Science. 2025;5(6):467-80.

5. Fox S. Online health search 2006. Pew Internet & American Life Project; 2006.

6. Gluskin RT, Johansson MA, Santillana M, Brownstein JS. Evaluation of Internet-based dengue query data: Google Dengue Trends. PLoS neglected tropical diseases. 2014;8(2):e2713.

7. Chan EH, Sahai V, Conrad C, Brownstein JS. Using web search query data to monitor dengue epidemics: a new model for neglected tropical disease surveillance. PLoS neglected tropical diseases. 2011;5(5):e1206.

8. Althouse BM, Ng YY, Cummings DA. Prediction of dengue incidence using search query surveillance. PLoS Negl Trop Dis. 2011;5(8):e1258.

9. Husnayain A, Fuad A, Lazuardi L. Correlation between Google Trends on dengue fever and national surveillance report in Indonesia. Global Health Action. 2019;12(1):1552652.

10. Chen Y, Zhang Y, Xu Z, Wang X, Lu J, Hu W. Avian Influenza A (H7N9) and related Internet search query data in China. Scientific reports. 2019;9(1):10434.

11. Teng Y, Bi D, Xie G, Jin Y, Huang Y, Lin B, et al. Dynamic forecasting of Zika epidemics using Google trends. PloS one. 2017;12(1):e0165085.

12. Google Flu Trends. Google Flu Trends [Internet]. Google.org; 2013 [cited 2020 Dec 22]. Available from: `https://web.archive.org/web/20121022154915/http://www.google.org/flutrends/about/how.html`.

13. Ginsberg J, Mohebbi MH, Patel RS, Brammer L, Smolinski MS, Brilliant L. Detecting influenza epidemics using search engine query data. Nature. 2009;457(7232):1012-4.

14. Won M, Marques-Pita M, Louro C, Gonçalves-Sá J. Early and real-time detection of seasonal influenza onset. PLoS computational biology. 2017;13(2):e1005330.

15. De Toni G, Consonni C, Montresor A. A general method for estimating the prevalence of influenza-like-symptoms with Wikipedia data. Plos one. 2021;16(8):e0256858.

16. Liu J, Suzuki S. Real-Time Detection of Flu Season Onset: A Novel Approach to Flu Surveillance. International Journal of Environmental Research and Public Health. 2022;19(6):3681.

17. Morris M, Hayes P, Cox IJ, Lampos V. Neural network models for influenza forecasting with associated uncertainty using Web search activity trends. PLoS Computational Biology. 2023;19(8):e1011392.

18. Ning S, Hussain A, Wang Q. Incorporating connectivity among Internet search data for enhanced influenza-like illness tracking. Plos one. 2024;19(8):e0305579.

19. Princess PJB, et al. LLM-Assisted Keyword Curation for Early Disease Outbreak Detection Using Search Trends. In: 2025 4th International Conference on Applied Artificial Intelligence and Computing (ICAAIC). IEEE; 2025. p. 478-83.

20. Lazer D, Kennedy R, King G, Vespignani A. The parable of Google Flu: traps in big data analysis. Science. 2014;343(6176):1203-5.

21. Towers S, Afzal S, Bernal G, Bliss N, Brown S, Espinoza B, et al. Mass media and the contagion of fear: the case of Ebola in America. PloS one. 2015;10(6):e0129179.

22. Olson DR, Konty KJ, Paladini M, Viboud C, Simonsen L. Reassessing Google Flu Trends data for detection of seasonal and pandemic influenza: a comparative epidemiological study at three geographic scales. PLoS Comput Biol. 2013;9(10):e1003256.

23. Tizzoni M, Panisson A, Paolotti D, Cattuto C. The impact of news exposure on collective attention in the United States during the 2016 Zika epidemic. PLoS computational biology. 2020;16(3):e1007633.

24. Yang S, Santillana M, Kou SC. Accurate estimation of influenza epidemics using Google search data via ARGO. Proceedings of the National Academy of Sciences. 2015;112(47):14473-8.

25. Mena I, Nelson MI, Quezada-Monroy F, Dutta J, Cortes-Fernández R, Lara-Puente JH, et al. Origins of the 2009 H1N1 influenza pandemic in swine in Mexico. Elife. 2016;5:e16777.

26. Brammer L, Blanton L, Epperson S, Mustaquim D, Bishop A, Kniss K, et al. Surveillance for influenza during the 2009 influenza A (H1N1) pandemic–United States, April 2009–March 2010. Clinical Infectious Diseases. 2011;52(suppl_1):S27-35.

27. Devaux I, Kreidl P, Penttinen P, Salminen M, Zucs P, Ammon A, et al. Initial surveillance of 2009 influenza A (H1N1) pandemic in the European Union and European economic area, April–September 2009. Eurosurveillance. 2010;15(49):19740.

28. Fox SJ, Miller JC, Meyers LA. Seasonality in risk of pandemic influenza emergence. PLoS computational biology. 2017;13(10):e1005749.

29. World Health Organization. Pandemic (H1N1) 2009 – update 112. World Health Organization; 2010. Accessed: YYYY-MM-DD. Available from: https://iris.who.int/bitstream/handle/10665/78414/9789241503051_eng.pdf?sequence=1.

30. Dawood FS, Iuliano AD, Reed C, Meltzer MI, Shay DK, Cheng PY, et al. Estimated global mortality associated with the first 12 months of 2009 pandemic influenza A H1N1 virus circulation: a modelling study. The Lancet infectious diseases. 2012;12(9):687-95.

31. World Health Organization. WHO COVID-19 Dashboard: Deaths [Internet]. World Health Organization; 2025 [cited 2025 Feb 11]. Available from: https://data.who.int/dashboards/covid19/deaths.

32. Panning M, Eickmann M, Landt O, Monazahian M, Ölschläger S, Baumgarte S, et al. Detection of influenza A (H1N1) v virus by real-time RT-PCR. Eurosurveillance. 2009;14(36):19329.

33. Teymouri M, Mollazadeh S, Mortazavi H, Ghale-Noie ZN, Keyvani V, Aghababaei F, et al. Recent advances and challenges of RT-PCR tests for the diagnosis of COVID-19. Pathology-Research and Practice. 2021.

34. Rosen JM, Warzecha HR. Coronavirus Variant Emergence and Epidemiology. In: The COVID-19 Pandemic: Science, Technology, and the Future of Healthcare Delivery. Springer; 2024. p. 193-206.

35. Wang Y, Tang CY, Wan XF. Antigenic characterization of influenza and SARS-CoV-2 viruses. Analytical and Bioanalytical Chemistry. 2022;414(9):2841-81.

36. World Health Organization. Pandemic influenza vaccine manufacturing process and timeline [Internet]. World Health Organization; 2009 [cited 2025 Feb 11]. Available from: https://www.who.int/news/item/06-08-2009-pandemic-influenza-vaccine-manufacturing-process-and-timeline.

37. King ML. How manufacturing won or lost the COVID-19 vaccine race. Vaccine. 2024.

38. Da Costa VG, Saivish MV, Santos DER, de Lima Silva RF, Moreli ML. Comparative epidemiology between the 2009 H1N1 influenza and COVID-19 pandemics. Journal of infection and public health. 2020;13(12):1797-804.

39. Wasim A, Truong J, Bakshi S, Majid U. A systematic review of fear, stigma, and mental health outcomes of pandemics. Journal of Mental Health. 2023;32(5):920-34.

40. Peláez-Ballestas I, Infante-Castañeda C, Giraldo-Rodríguez L. Comparison between Covid-19 and influenza A (H1N1) pandemic experiences and risk perception in a Mexican university. salud pública de méxico. 2021;63(5):619-29.

41. Kogan NE, Clemente L, Liautaud P, Kaashoek J, Link NB, Nguyen AT, et al. An early warning approach to monitor COVID-19 activity with multiple digital traces in near real-time. arXiv preprint arXiv:200700756. 2020.

42. Dewhurst DR, Alshaabi T, Arnold MV, Minot JR, Danforth CM, Dodds PS. Divergent modes of online collective attention to the COVID-19 pandemic are associated with future caseload variance. arXiv preprint arXiv:200403516. 2020.

43. Liu D, Clemente L, Poirier C, Ding X, Chinazzi M, Davis JT, et al. A machine learning methodology for real-time forecasting of the 2019-2020 COVID-19 outbreak using Internet searches, news alerts, and estimates from mechanistic models. arXiv preprint arXiv:200404019. 2020.

44. Ayyoubzadeh SM, Ayyoubzadeh SM, Zahedi H, Ahmadi M, Kalhori SRN. Predicting COVID-19 incidence through analysis of google trends data in iran: data mining and deep learning pilot study. JMIR Public Health and Surveillance. 2020;6(2):e18828.

45. Lu T, Reis BY. Internet Search Patterns Reveal Clinical Course of Disease Progression for COVID-19 and Predict Pandemic Spread in 32 Countries. medRxiv. 2020.

46. Effenberger M, Kronbichler A, Shin JI, Mayer G, Tilg H, Perco P. Association of the COVID-19 pandemic with internet search volumes: a google trendstm analysis. International Journal of Infectious Diseases. 2020.

47. Grasso KL, Bell RA. Understanding health information seeking: A test of the risk perception attitude framework. Journal of health communication. 2015;20(12):1406-14.

48. Weaver III JB, Mays D, Weaver SS, Hopkins GL, Eroğlu D, Bernhardt JM. Health information–seeking behaviors, health indicators, and health risks. American journal of public health. 2010;100(8):1520-5.

49. Hone T, Palladino R, Filippidis FT. Association of searching for health-related information online with self-rated health in the European Union. The European Journal of Public Health. 2016;26(5):748-53.

50. Link E, Baumann E, Klimmt C. Explaining online information seeking behaviors in people with different health statuses: German representative cross-sectional survey. Journal of medical Internet research. 2021;23(12):e25963.

51. Li J, Zheng H. Online information seeking and disease prevention intent during COVID-19 outbreak. Journalism & Mass Communication Quarterly. 2022;99(1):69-88.

52. Kamalov F, Sulieman H, Alzaatreh A, Emarly M, Chamlal H, Safaraliev M. Mathematical methods in feature selection: A review. Mathematics. 2025;13(6):996.

53. Li Y, Li T, Liu H. Recent advances in feature selection and its applications. Knowledge and Information Systems. 2017;53(3):551-77.

54. FluNet;. Accessed: 2020-06-18. https://www.who.int/influenza/gisrs_laboratory/flunet/en/.

55. Our World in data;. Accessed: 2020-08-20. https://github.com/owid/covid-19-data/tree/master/public/data.

56. Google Trends. Google Trends; 2025. Accessed: 2025-02-10. Available from: https://trends.google.com/trends/.

57. Sood G, Laohaprapanon S. Vanderbilt TV News Abstracts. Harvard Dataverse; 2020. Available from: https://doi.org/10.7910/DVN/BP2JXU. doi:10.7910/DVN/BP2JXU.

58. Cloud M. Media Cloud: Global News Database; 2023. Accessed: 2023-05-15. https://search.mediacloud.org/search.

59. SciPy Community. SciPy: Scientific Computing Tools for Python; 2023. Accessed: 2025-02-09. Available from: https://scipy.org.

60. LinearRegression;. https://scikit-learn.org/stable/modules/generated/sklearn.linear_model.LinearRegression.htmll.

61. RandomForestRegressor;. https://scikit-learn.org/stable/modules/generated/sklearn.ensemble.RandomForestRegressor.html.

62. Raschka S. Mlxtend: A library of Python tools and extensions for data science; 2023. Version retrieved: 2023-05-15. Available from: https://pypi.org/project/mlxtend/.

63. Ma KC. Genomic surveillance for SARS-CoV-2 variants: circulation of omicron lineages—United States, January 2022–May 2023. MMWR Morbidity and mortality weekly report. 2023;72.

64. Benjamini Y, Hochberg Y. Controlling the false discovery rate: a practical and powerful approach to multiple testing. Journal of the Royal statistical society: series B (Methodological). 1995;57(1):289-300.

65. Breiman L, Friedman J, Olshen RA, Stone CJ. Classification and regression trees. Chapman and Hall/CRC; 2017.

66. Meyer H, Pebesma E. Predicting into unknown space? Estimating the area of applicability of spatial prediction models. Methods in Ecology and Evolution. 2021;12(9):1620-33.

67. Hengl T, Nussbaum M, Wright MN, Heuvelink GB, Gräler B. Random forest as a generic framework for predictive modeling of spatial and spatio-temporal variables. PeerJ. 2018;6:e5518.

68. Wiemken TL, Khan F, Puzniak L, Yang W, Simmering J, Polgreen P, et al. Seasonal trends in COVID-19 cases, hospitalizations, and mortality in the United States and Europe. Scientific reports. 2023;13(1):3886.

69. Quinn GA, Connolly M, Fenton NE, Hatfill SJ, Hynds P, Ohaiseadha C, et al. Influence of seasonality and public-health interventions on the COVID-19 pandemic in northern Europe. Journal of Clinical Medicine. 2024;13(2):334.

70. Doupe P, Faghmous J, Basu S. Machine learning for health services researchers. Value in Health. 2019;22(7):808-15.

71. Lever J, Krzywinski M, Altman N. Points of significance: model selection and overfitting. Nature methods. 2016;13(9):703-5.

72. Cheong KH, Li K, Yu D, Zhao X. Forecasting dengue cases through time-series modeling with Google Trends and deep neural networks. Chaos, Solitons & Fractals. 2025;201:117290.

73. Mesquita S, Perfeito L, Paolotti D, Gonçalves-Sá J. Epidemiological methods in transition: Minimizing biases in classical and digital approaches. PLOS Digital Health. 2025;4(1):e0000670.

74. Atias D, Ashri S, Goldbourt U, Benyamini Y, Gilad-Bachrach R, Hasin T, et al. Machine learning in epidemiology: An introduction, comparison with traditional methods, and a case study of predicting extreme longevity. Annals of Epidemiology. 2025.

Figure 1

Click here to access/download;Figure;Figure_1.tif

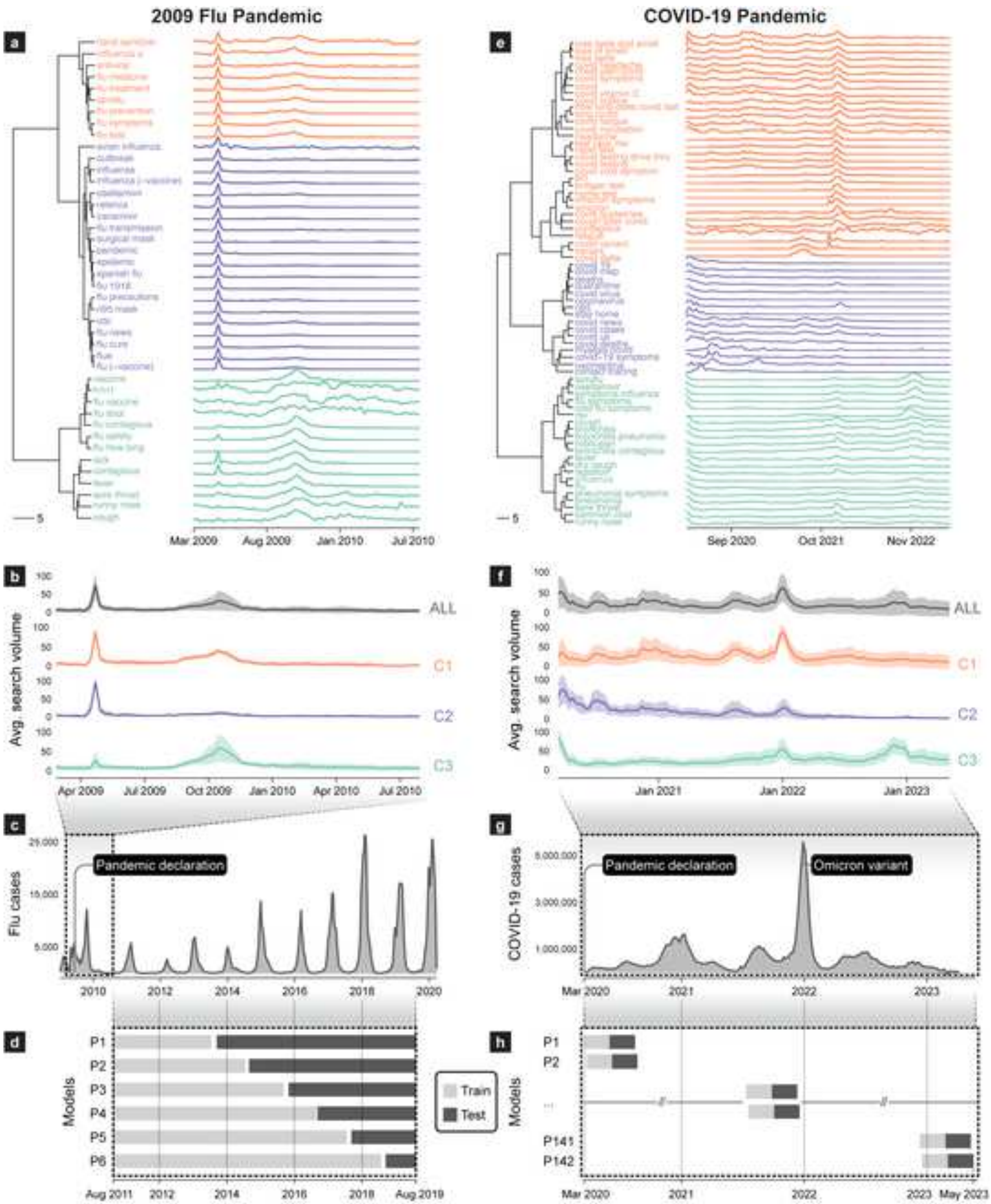

Figure 2

Click here to access/download;Figure;Figure_2.tif

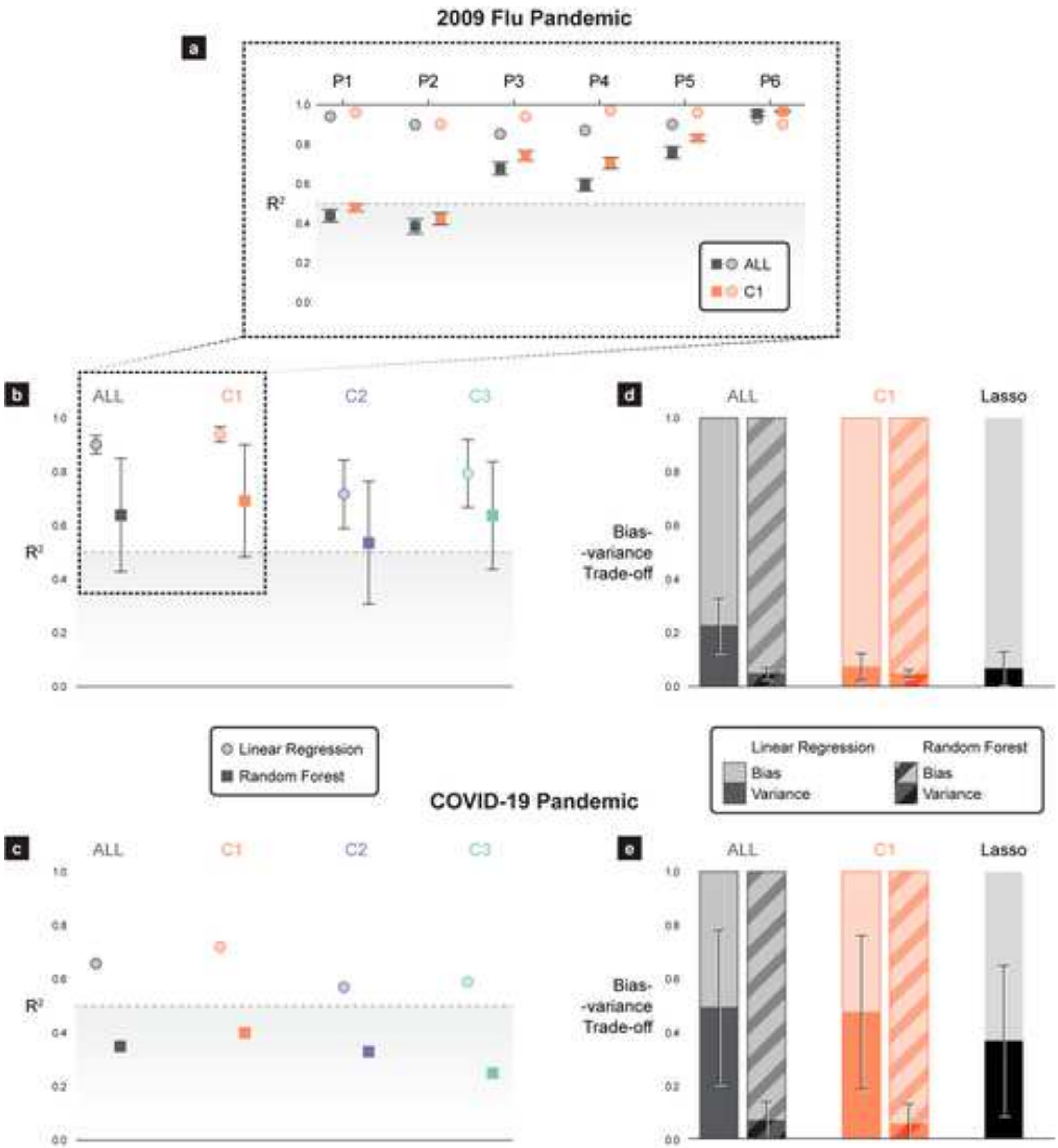

Figure 3

Click here to access/download;Figure;Figure_3.tif

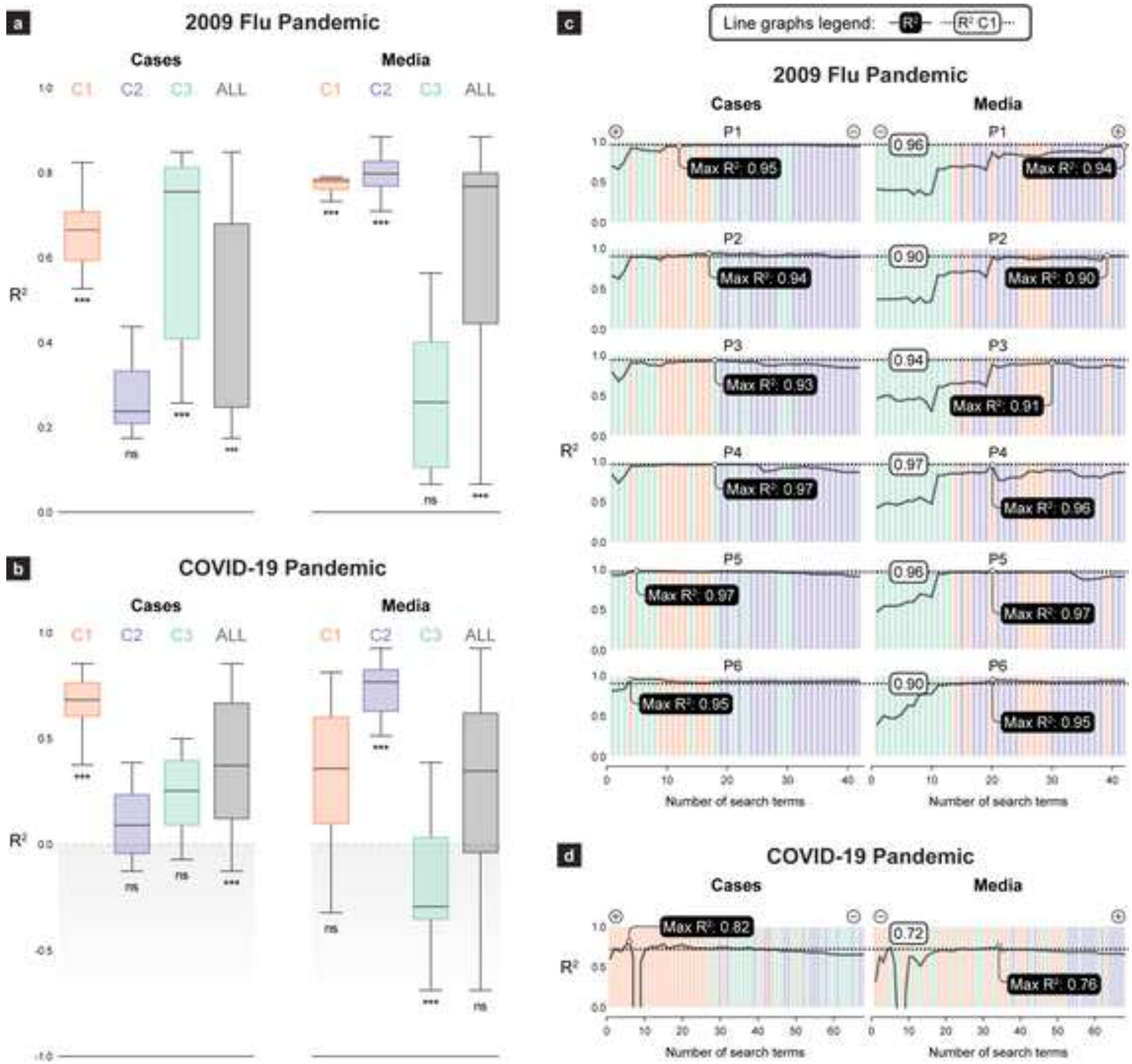

Click here to access/download
Supporting Information
Supporting information.pdf

Learning from Pandemics: Feature Clustering for Robust Digital Disease Nowcasting

Sara Mesquita[1,2], João Loureiro[1], Cláudio Haupt Vieira[3], Lília Perfeito[1], Joana Gonçalves-Sá[1,3,4*]

1 LIP – Laboratory for Instrumentation and Experimental Particle Physics, Avenida Prof. Gama Pinto 2, 1600-078 Lisboa, Portugal
2 NOVA Medical School, Universidade NOVA de Lisboa, Campo dos Mártires da Pátria 130, 1169-056 Lisboa, Portugal
3 NOVA School of Business and Economics, Universidade NOVA de Lisboa, Rua da Holanda, 2775-405 Carcavelos, Portugal
4 NOVA LINCS, Faculdade de Ciências e Tecnologia, Universidade NOVA de Lisboa, 2829-516 Caparica, Portugal

* Corresponding author: joanagsa@lip.pt

Abstract

Disease now-casting increasingly relies on non-traditional data, such as online activity, which offer fast, inexpensive insights but are often noisy, unstructured, and influenced by behavioral confounders. Models using search engine data have frequently suffered from overfitting and unstable performance. However, pandemics may help distinguish true disease signals from behavioral noise, as both tend to surge during outbreaks.

We propose a hybrid approach to improve prediction that combines model evaluation with behavioral insight. We apply simple supervised and unsupervised methods to different flu and COVID-19 datasets, using the 2009 H1N1 and 2020 COVID-19 pandemics as training periods. In both cases, selecting a curated subset of search terms improved model performance, reduced overfitting, and increased stability, consistently outperforming models that used search terms indiscriminately.

These results suggest that feature curation informed by behavioral insights can enhance now-casting beyond purely statistical approaches. Our method offers a scalable and adaptable framework for integrating digital epidemiology into public health surveillance.

Introduction

Infectious diseases present significant health risks worldwide. To mitigate these risks, public health institutions have established surveillance and response systems designed to detect and ideally stop outbreaks quickly and accurately. Typical monitoring includes complementary approaches, such as sentinel doctors and testing laboratories, but even when these mechanisms are effectively in place, detection and mitigation efforts can fail, leading to epidemics and, in rarer cases, pandemics. As surveillance mechanisms relying solely on highly trained personnel are often expensive, limited, and slow, it has been extensively argued that traditional methods should be complemented with "Digital Era" tools, such as online activity, mobility patterns, or digital contact-tracing [1–4].

In particular, internet searches for disease-related information have shown great promise [5] when used to follow epidemics such as Dengue [6–9], Avian Influenza [10],

and Zika [11]. Launched in 2008, Google Flu Trends (GFT) [12] had already showcased the potential of online-based surveillance by predicting the timing and magnitude of human influenza activity through the aggregation of flu-related search trends. Unlike traditional surveillance methods, GFT provided near real-time reports without relying on clinical or lab data [13]. More recent studies have found strong evidence that collective online searches for health information from flu-infected individuals, can be a reliable indicator of flu activity [14–18].As the use of online search data for disease surveillance has matured, attention has shifted from whether these data are useful to how the most informative search terms should be selected. Even with recent advances in artificial intelligence, careful keyword curation remains essential for identifying the search terms that carry the most relevant information for disease prediction [19].

However, flu infection is not the sole (and perhaps not even the strongest) motivation behind flu-related online searches [20], and distinguishing between searches related to actual cases and searches performed for other reasons (e.g. fear, hype) is challenging. This is particularly true during extraordinary events when individuals are expected to have varying degrees of interest, ranging from curiosity to anxiety, to actual disease [21], resulting in high signal (infection-driven searches) and high noise (searches driven by other factors). In fact, the GFT model missed the first wave of the 2009 flu pandemic and overestimated the magnitude of the severe 2013 seasonal flu outbreak in the USA [20, 22]. Similarly, when studying the 2016 Zika epidemic, Tizzoni et al. found a poor correlation between information-seeking and disease dynamics, with media attention being an important confounding factor [23]. As a result, several researchers have become skeptical about the potential of online searches as a monitoring tool, reasonably arguing that it is too prone to overfitting and spurious correlations. Others have proposed improvements, such as including temporal dependencies through autoregressive terms (e.g., ARGO [18, 24]). These approaches refine predictions, but are often still limited by timely data availability, assume typical seasonality, with current events resembling past ones, or rely on black-box models that are difficult to interpret. Here, we argue that pandemics provide a natural experiment as they amplify not only cases, but also fears, online behaviors, media hype, etc. This high intensity can be leveraged to disentangle between behaviors, particularly online searches driven by actual disease (signal) from those driven by other factors (noise). Moreover, we hypothesize that the "true signal" identified during pandemics will be useful to nowcast seasonal disease outbreaks, as long as the disease characteristics (symptoms, progression) remains approximately the same.

The first pandemic of the 21st century was caused by the Influenza A strain (H1N1)09pdm (pH1N1 for short), which emerged in Mexico in February 2009 [25]. By June 2009, pH1N1 had spread globally, with around 30,000 confirmed cases in 74 countries. It exhibited a bi-phasic activity in most countries: a spring-summer wave and a more severe fall-winter wave [26, 27], coinciding with the common flu season in the Northern Hemisphere [28]. The pandemic was declared over in August 2010, with 18,449 laboratory-confirmed deaths, which were later revised by [29] to have been 15 times higher [30]. The second pandemic was caused by the SARS-CoV-2 virus, first identified in December 2019 in Wuhan, China. To date, COVID-19 has infected over 777 million people and killed more than 7 million worldwide [31]. While both the H1N1 and COVID-19 pandemics were global health crises triggered by respiratory viruses and taking place during the internet era, they differed significantly in virology, epidemiology, public health policy, and societal effects. Initially, both pandemics led to extensive research efforts and precise infection tracking through large-scale laboratory confirmations [32, 33]. Moreover, unlike the flu pandemic, in the case of COVID-19, testing policies varied widely between countries and over time, with laboratory testing eventually being supplanted by at-home rapid test kits. Negative results from the latter

were not systematically reported, leading to increased uncertainty in incidence estimates. Other important differences between the pandemics include the epidemiology of the various SARS-CoV-2 variants, which varied widely [34], the time it took from first infections to vaccination [35–37], duration of the pandemics, and peak dynamics, with COVID-19's spread being much less predictable, with large outbreaks occurring at irregular intervals, even during warm seasons. Additionally, SARS-CoV-2 proved to be much more infectious and severe than the pH1N1 flu strain, leading to a stronger public health response, including lockdowns, compulsory masking, and other restrictive non-pharmaceutical interventions [38].

Overall, COVID-19 raised significantly more public concern and anxiety [39, 40] and this was reflected in an increase in online activity, which became broadly used to explain and forecast COVID-19 disease dynamics [41–46]. Beyond volume, evidence shows that both real risk and risk perception shape online health information seeking [47], and that searching behaviors differ for people who feel ill versus healthy [48, 49]. Queries about institutions or public measures, by contrast, often rise with information exposure and broader public concern. Online searching is also positively associated with intentions to take preventive measures [50, 51].

Thus, we propose to learn from the variability and intensity (signal, noise, anxiety, etc.) of pandemic-related searches and ask: 1) Can these pandemic periods inform post-pandemic or seasonal disease trends? 2) Can we refine current supervised and unsupervised methods by incorporating behavioral insights?

We focus on the USA due to its high number of flu and COVID-19 cases, the availability of comprehensive data, and the occurrence of multiple COVID-19 waves. Recent reviews have emphasized that effective feature selection should identify informative predictors while reducing redundancy, thereby improving predictive performance, robustness, and model interpretability [52, 53]. However, relatively little attention has been paid to whether pandemic periods themselves can be exploited to improve feature selection. To address this gap, we propose a simple and explainable feature selection framework that leverages the high-intensity periods of pandemics as natural experiments to identify search terms with greater temporal robustness for disease nowcasting. Unlike conventional big data approaches, which primarily mitigate overfitting through statistical feature selection, our method prioritizes search terms that remain relevant across different outbreak phases, improving model interpretability and robustness. To evaluate this approach, we first describe the data sources and modelling approach, then assess its performance across influenza and COVID-19 case studies, and finally discuss its implications for the design of robust and interpretable disease nowcasting systems.

Materials and methods

Health data is considered sensitive under the EU GDPR. In this study only previously anonymized and aggregated data was used.

Data Sources

Infectious disease Data

Data for both the 2009 H1N1 flu pandemic and the COVID-19 pandemic were collected for the United States. Influenza: case numbers were obtained from the CDC's National Respiratory and Enteric Virus Surveillance System [54], covering March 2009 to August 2019, which includes the pandemic period (March 2009–August 2010) and post-pandemic period (August 2010–August 2019). The pandemic period was used

exclusively for feature selection (as described in the hierarchical clustering section below), while modeling was conducted on post-pandemic seasonal flu data. COVID-19: case numbers were collected from March 15, 2020, to May 7, 2023, from ECDC's Our World in Data [55]. This period aligns with key milestones: the declaration of COVID-19 as a global pandemic by the World Health Organization (WHO) on March 11, 2020, major outbreak waves and vaccination roll out, and the official end of the COVID-19 public health emergency in the United States on May 11, 2023. While there are already two years of cases after the official end of the pandemic, the way the data is gathered and reported changed considerably, making training models very difficult. Therefore, in the case of COVID-19 both clustering and modeling were restricted to within-pandemic data.

Search Trend data

Data extracted from GT returns the normalized, scaled and aggregated total number of searches for a particular query, in a specified geographical region and time range. All data is anonymous and it is virtually impossible to identify individual searchers. Since at the time of writing, Google did not provide an official API for Google Trends, the *pytrends* Python package was used instead. This automates requests to the same widgets shown in the public web interface (e.g., Interest Over Time) and returns the same normalized index values [56]. The analyses relied on the exported index values, not on any *pytrends*-specific transformation. Data collection was structured around the 'TrendReq' function, configured to retrieve search trends from the United States in English (using the parameters hl="en-us" for language and tz=360 for timezone). Details regarding the extraction dates can be found in the Data processing section. To verify consistency, the *pytrends* outputs were bench-marked against manual downloads from the Google Trends website for the same queries, locations, and dates (see S8–S9 Figs in the Supporting information), by calculating Spearman and Pearson correlations.

Search terms were selected to cover various aspects of pandemic and seasonal flu, and COVID-19, such as symptoms, treatments, personal care, institutions, and pandemic-specific terms. The selection process was conducted in three steps: First, a literature review was performed to identify commonly used terms, which were then supplemented with additional terms proposed by the authors. Second, these terms were queried in Google Trends, and those suggested by the pytrends 'related queries' option were added to the list. Third, terms with low search volumes, characterized by Google Trends data containing many 'zeros' interspersed with occasional high values were excluded. After this process, 42 flu-related weekly search trends and 68 COVID-19-related trends were kept. Both word lists are shown in Fig 1 panels A and E, and are provided in S1 and S2 Tables.

Media data

Flu: Mentions to 'flu' or 'influenza' were collected from 2008-08-10 to 2013-04-07. TV mentions were collected from the Vanderbilt Television News Archive [57], which includes major U.S. television networks: NBC, CBS, CNN, FOX, and MSNBC. Print media data were obtained from The New York Times API (https://developer.nytimes.com/). COVID19: Mentions of "COVID-19" from December 1, 2019, to August 31, 2023, were collected using Media Cloud [58], a platform that aggregates media coverage. The final dataset includes 249 sources, covering a diverse range of major national newspapers, online news platforms, and broadcast media. To align temporal resolution with other datasets, the raw Media Cloud data were resampled to a weekly scale, with weeks starting on Mondays. At the time of preparing

this article, Media Cloud did not provide data as far back as 2010, and could not be used to created the flu media dataset.

Data processing

Search trends data normalization

Google Trends normalizes search volume by dividing each data point by the maximum number of searches for that term within a given geographic region and time frame, resulting in all series being scaled to the same maximum value. While every search query includes a peak value of 100, not all contain a zero (i.e., if there were no weeks with search volumes below 1% of the maximum daily value). Additionally, Google Trends applies a predefined temporal resolution based on the extraction period (daily resolution for extractions covering less than 9 months, weekly if periods are between 9 months and 5 years, and monthly if longer than 5 years). To standardize the data a multi-step approach was implemented. 1) a long-period extraction was performed for each search term across the full study period. In the case of the flu, from 2005-01-01 to 2020-12-31, for COVID-19, from 2020-01-05 to 2023-05-28; 2) to capture daily variations in search frequency, month-to-month extractions were conducted; 3) the daily search volume was normalized so that the sum of all daily values within each month was 1; 4) the timeseries were combined by multiplying the normalized daily frequency by the corresponding monthly search volume; 5) daily data were aggregated into weekly values by computing the average daily volume for each week, resulting in an extended dataset with a consistent weekly resolution; 6) The entire dataset was rescaled between 0 and 100 to maintain comparability across time series. Code demonstrating this process is available in the repository under the name 'Extended Google Queries'.

Defining time windows

The modeling approach differed between the two pandemics due to data availability constraints and differences in disease seasonality. Flu: the pandemic period (defined as 2009-03-01 to 2010-08-01) was used only for cluster definition via hierarchical clustering. Modeling was conducted exclusively on post-pandemic seasonal flu data (2010-08-22 to 2019-08-11). For modeling, the different periods (P) for training and testing models were defined through visual inspection over nine years of data, ensuring they captured recurring seasonal patterns. To closely replicate real-time now-casting scenarios, six different breakpoints were selected, in the summers of 2013, 2014, 2015, 2016, 2017, and 2018 (Fig 1 D). The breakpoint was chosen to coincide specifically with the lowest number of cases so it could be used for training, and predict complete flu seasons. Exact breakpoint dates are provided in S3 Table.

COVID-19: data was extracted between 2020-03-15 and 2023-05-07. As there are several non-periodic peaks in the cases time series, there is no clear way to separate them. The choice was to use every time point as a breakpoint and train the model in 12 week sliding-windows.

Hierarchical clustering

Hierarchical clustering was applied to the (standardized) Google search terms timeseries, by computing the pairwise Euclidean distances between each term time series and using Ward's linkage method for clustering. Resulting dendrograms in Fig 1A and 3E.

Analyses were performed using Python, using the scipy.cluster.hierarchy.dendrogram function from the SciPy library [59]. The number of clusters was determined through visual inspection of the dendrogram structure, ensuring that meaningful groupings were captured. Cluster labels were assigned after clustering based on the most frequent terms

in each cluster and on their temporal behavior. They were not used as targets or inputs during model training, and they do not change how models are estimated or evaluated, they were only used to describe groups of search terms and to define the C1, C2, and C3 feature sets. This analysis was conducted separately for the H1N1 pandemic, using data from 2009-03-01 to 2010-08-01, and for COVID-19, using data from 2020-03-15 to 2023-05-07.

Modeling approach

Linear Regression

To model the relationship between search trends and infection cases, a linear regression model was implemented, of the form:

$$I_i = \beta_0 + \beta_1 \times W_1 + \beta_2 \times W_2 + ... + \beta_n \times W_n + \epsilon_i \quad (1)$$

Where I_i represents the number of infections in week i, β_0 is the intercept, β_1 to β_n are the coefficients of each search term and ϵ_i is the error. Here, W_1 to W_n denote the weekly search volumes for each selected search term, capturing fluctuations in online interest over time. These terms serve as predictor variables, allowing the model to quantify how variations in search behavior relate to changes in infection counts. The coefficients were estimated to minimize the sum of the square of the errors across all weeks. The regression was implemented in Python using sklearn.linear_model.LinearRegression [60] with default parameters. The input features consisted of Google search terms selected as previously described, while the target variable was the number of infection cases per week. To evaluate the contribution of different search term cluster dynamics, separate models were run using all search terms (All) as well as specific clusters: Cluster 1 (C1), Cluster 2 (C2), and Cluster 3 (C3). Each cluster model followed the same training and evaluation procedure to ensure comparability.

Lagged cases and search terms were introduced to the COVID-19 models to capture the delay between online search behavior and infection trends. As the pandemic was still ongoing, unlike the 2009 flu pandemic, it was necessary to incorporate temporal dependencies to improve predictive accuracy. To account for this, four weeks of lagged cases were included, and each search term was lagged by 0, 1, 2, or 3 weeks, following an approach similar to the implementation of the ARGO model [24]. The optimal lag for each search term was determined independently for each model as a hyperparameter, selecting the lag that best predicted cases in a univariate regression.

Random Forest

A Random Forest regression model was implemented to capture potential nonlinear relationships between search trends and infection cases. The model followed the same training and evaluation procedures as linear regression and LASSO. Training was performed using sklearn.ensemble.RandomForestRegressor [61], with hyperparameters—number of estimators, maximum features, and maximum depth—optimized through cross-validation using GridSearchCV. The tested values for these parameters were selected from the ranges [10, 20, 50, 100, 200, 500, 1000] for the number of estimators, [0.6, 0.8, ”auto”, ”sqrt”] for maximum features, and [2, 4, 5, 6] for maximum depth.

To account for variability in model performance, multiple random seeds were used. For the 2009 Flu pandemic, each model was trained using 100 different random seeds, ensuring robust evaluation across different initial conditions. For COVID-19, given that

142 separate models were already running, 10 random seeds per model were used, balancing computational feasibility with performance reliability.

Evaluation metrics

Bias-variance trade-off

To estimate bias and variance, the mlxtend Python package [62] and the evaluate.bias_variance_decomp algorithm were used. This method decomposes the total error into bias and variance by performing bootstrapped resampling iterations over the training data. For both random forest and linear regression flu models, 200 resamples were performed; for Covid-19 random forest, only 10 were used because it would not be feasible to run hundreds of samples over 142 models. Bias was defined as the mean error of the models for each week, while variance was computed as the mean variance of the model predictions for each week. This decomposition allowed for a detailed assessment of the trade-off between underfitting and overfitting across different modeling approaches.

LASSO regression

Implemented as an alternative to linear regression to perform feature selection while minimizing multicollinearity. The model followed the same training and evaluation procedures as linear regression. Unlike standard linear regression, LASSO incorporates L1 and L2 regularization, shrinking less informative coefficients to zero. Hyperparameters were optimized using cross-validation with ElasticNetCV (in all cases, the regularization that had the best performance was L1), and the selected coefficients were analyzed to determine the most predictive search terms across different time frames.

Train-Test split sensitivity analysis

To assess the impact of train-test split selection on model performance, the variability in $\mathbf{R^2}$ scores across different rolling window periods was analyzed (see Modeling Approach). This analysis was conducted only for COVID-19 using Linear Regression, as it provides an interpretable baseline to isolate the effects of train-test split choices without introducing additional complexity from nonlinear models. Results were compared for the "All" and C1 search term sets, evaluating whether certain train-test split points systematically led to better or worse performance. $\mathbf{R^2}$ scores were computed across 142 overlapping models to quantify this variability.

Variant-stratified evaluation and longitudinal feature-importance tests

For the variant-stratified analyses, the COVID-19 timeline was divided into seven non-overlapping phases using inclusive start and end dates to tag each week: Pre-Alpha (March 15, 2020 to December 13, 2020), Alpha (December 14, 2020 to June 20, 2021), Delta (June 21, 2021 to December 12, 2021), Omicron BA.1 (December 13, 2021 to March 20, 2022), Omicron BA.2 (March 21, 2022 to June 19, 2022), Omicron BA.5 (June 20, 2022 to October 16, 2022), and Omicron XBB + (October 17, 2022 to May 7, 2023) [63]. Phase-specific performance metrics (RMSE and R^2) were computed using only the weekly predictions within each phase and also calculated a four-week rolling RMSE to capture short-term fluctuations. To examine whether individual search terms changed in predictive relevance, weekly Random Forest feature importances from the "ALL" model were used, and plotted in a term-by-week heatmap with vertical lines marking the variant boundaries. For each term, it was then tested whether importance

decreased after January 2022 using a one-tailed Mann–Whitney U test (pre-2022 vs post-2022), applying Benjamini–Hochberg FDR correction [64] at 0.05. Terms with a significant decline are marked with an asterisk in the heatmap (S14 Fig). This procedure quantifies when specific search behaviors (e.g., "loss of smell") lost predictive value as public perception and testing practices evolved.

Random feature selection comparisons

To test whether the C1 feature set captures meaningful behavioral patterns, its performance was compared against randomly selected feature sets of the same size and evaluated C1's percentile rank within the resulting distribution of random models.

For each flu season, sets of 9 search terms were randomly sampled (matching the number of terms in C1) and models were trained on them. This process was repeated 1000 times per season to generate a distribution of R^2 scores. The performance of C1 was then compared with the average performance of these random models and C1's percentile rank was computed within the distribution to determine the proportion of random selections with lower performance. A percentile rank of 100% indicates that C1 outperformed all random selections, while lower ranks suggest that some random sets performed equally well or better.

All models were evaluated using R^2 and bias-variance decomposition to assess predictive performance and generalizability.

Correlation analysis

Correlation analyses were conducted across multiple time periods to evaluate the relationship between search trends, confirmed cases, and media coverage. While the primary feature selection step was performed using data from the pandemic periods (March 2009 to August 2010 for H1N1, and March 2020 to May 2023 for COVID-19), correlation analyses were also extended to post-pandemic flu seasons to assess the stability of these relationships over time.

For each period, Pearson correlation coefficients were computed between the weekly search volumes of each term and the number of confirmed cases. For the flu pandemic, correlations were calculated separately for each predefined training period (P1–P6) to determine how search term relevance varied across different flu seasons. This was implemented in Python using the `scipy.stats.pearsonr` function. Additionally, correlation analyses were performed between search trends and weekly media mentions of flu and COVID-19.

To evaluate the impact of correlation-based feature selection, two approaches were tested: selecting the 10% most correlated search terms during the pandemic period, filtered for statistical significance ($p < 0.05$), and identifying the minimum number of highly correlated search terms per flu season required to achieve maximum predictive performance.

Results

Online disease-related searches show distinct patterns

The primary objective of this study was to develop a simple, cost-effective, and reliable now-casting system that balances accuracy and interpretability. Following previous research [6–9], we used Google search volumes as a proxy for epidemic activity in the population. As a first step, we examined whether seemingly similar search terms

exhibited distinct temporal patterns, as differences in search dynamics may reflect variations in underlying motivations.

To explore this, we analyzed the temporal behavior of multiple related search terms during the 2009 H1N1 and COVID-19 pandemics in the USA. Pandemic periods were selected as they amplify both actual infection-driven searches and fear or other-driven interests, providing a clearer distinction between signal (searches linked to disease incidence) and noise (searches influenced by external factors). Applying hierarchical clustering to these terms identified three distinct groups in both pandemics (Fig 1A and E). Fig 1B and 1F) illustrate the average search volume of all search terms ("All"), with gray shading highlighting periods of higher variance in search dynamics. Cluster labels were assigned after clustering from the most frequent terms and their temporal behavior and are used only to aid interpretation at the population level.

For H1N1, Cluster 1 (C1, orange) exhibited a sharp peak in early 2009, followed by a broader secondary increase. Cluster 2 (C2, purple), showed a single peak in early 2009 with little subsequent activity, and Cluster 3 (C3, green), displayed a noisier pattern, with the largest peak occurring in late 2009 (Fig 1B).

For COVID-19, C1 (orange) initially fluctuated before reaching a sharp peak in December–January 2021, coinciding with the highest number of cases (Fig 1G). C2 (purple), exhibited an early pandemic peak followed by irregular spikes, and C3 (green) showed multiple peaks in early 2020, late 2021, and winter 2022, with a smoother trajectory overall (Fig 1F).

Since Google Trends extrapolates search volumes from sampled data and periodically updates its outputs, we performed two technical controls. First, we tested whether the observed clusters were robust to changes in data retrieval timing. Across different collection periods, including those spanning multiple years, the clustering structure remained stable (S1 Fig), indicating that the observed patterns likely reflected real dynamics. Second, we further tested whether the results were an artifact of the data retrieval tool (*pytrends*), by comparing these time series with the official Google Trends CSV exports (two queries per cluster). For all tested terms, correlations were very high (all Pearson and Spearman coefficients > 0.94, Tables S7 (flu) and S8 (COVID)), and temporal profiles were similar (S8 and S9 Figs). These controls confirm the reliability of the automated pipeline in producing consistent estimates of search interest over time, supporting its use for epidemiological applications that require periodic data updates. Thus, despite largely following epidemic waves (S7 Fig in the Supporting information), different search terms exhibit distinct temporal signatures.

For both pandemics, search terms were automatically grouped into three clusters , for each disease, with the COVID-19 clusters being less well-defined than H1N1's, as shown by higher variance in centroid curves (Fig 1F) and by shorter branches in the hierarchical clustering (Fig 1E). This suggests that different search motivations contribute to these dynamics. If so, some clusters may be more associated with actual disease and those should more predictive of disease trends than other clusters (and even than individual search-terms). Still, in what follows, cluster labels are used to summarize group behavior, and should not be read as claims about individual motivations.

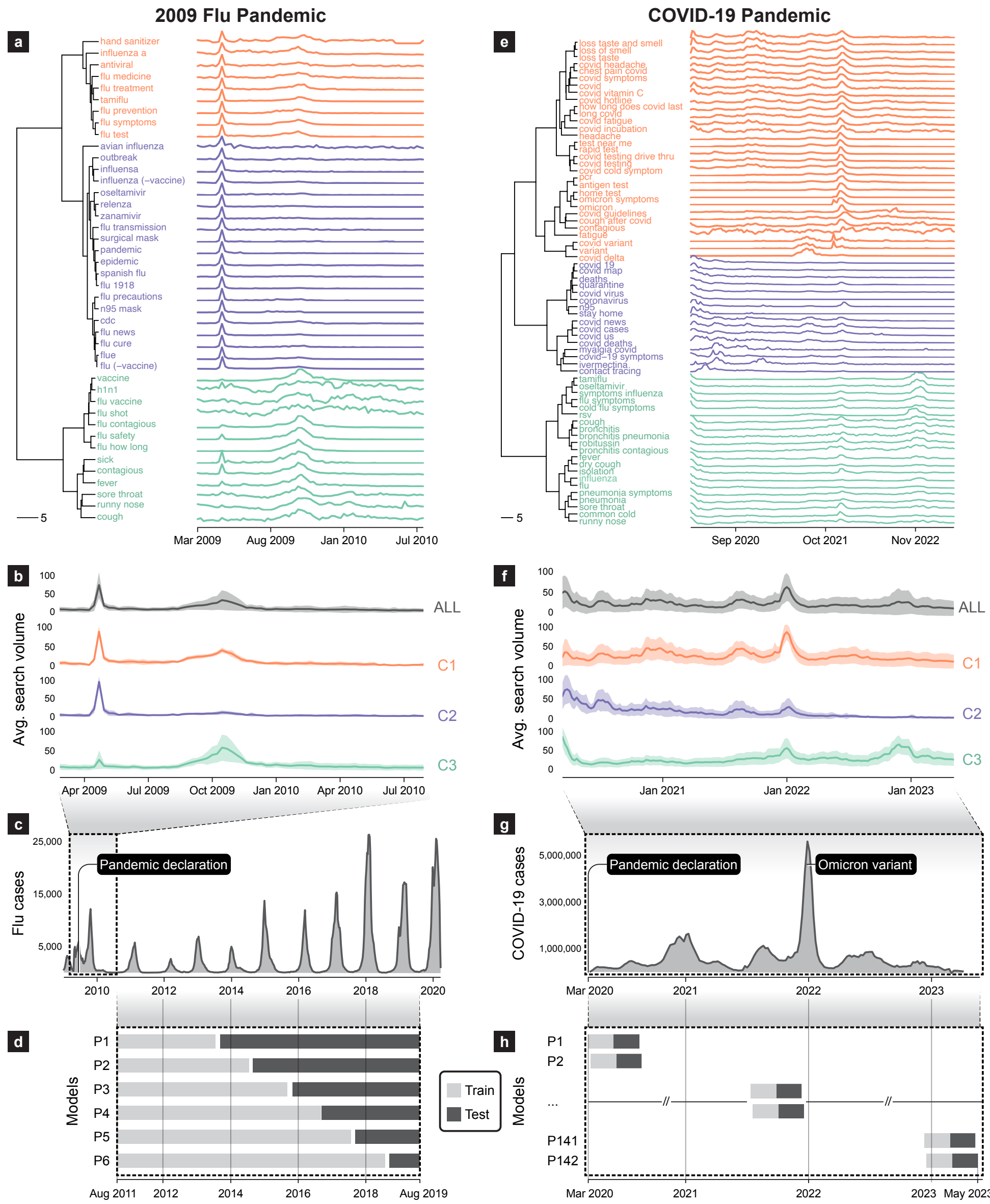

Fig 1. Search term clustering, infection trends, and modeling approach for the 2009 H1N1 and COVID-19 pandemics. (a, e) Hierarchical clustering of search terms into three clusters (C1, C2, C3) based on temporal search volume patterns. (b, f) Cluster centroids with variance indicated by shaded areas. (c, g) Reported flu (c) and COVID-19 (g) cases over time, with key pandemic milestones highlighted. Shaded areas indicate the timeframe used for clustering. (d, h) Training and testing periods for now-casting models. For H1N1 (d), post-pandemic flu seasons were used for train and test splits. For COVID-19 (h), a rolling window approach adapted models across pandemic phases.

Pandemic periods as a natural experiment for improving seasonal flu now-casting

We hypothesized that "true signal" search terms identified during pandemics remain predictive beyond them and could enhance seasonal now-casting, improving our ability

to track disease dynamics outside of crisis conditions.

To test this, we evaluated whether search terms identified during the 2009 flu pandemic could accurately predict seasonal flu trends in subsequent years. We compared models trained using all search terms with those trained using terms based on single clusters (Fig 1A). These models were validated across six seasonal flu periods (P1–P6), of different intensities (Fig 2C, D). Predictive performance was assessed using the coefficient of determination (R^2) for Linear Regression and Random Forest models (Fig 2A and B).

Overall, Linear Regression outperformed Random Forest in five out of six test cases, particularly when the validation period included severe flu seasons (i.e., seasons with high peaks of reported cases), with 2018 being the most glaring example. This can be explained by the large differences in amplitude between training and testing data. Random forests perform regression tasks by building trees where each branch partitions the dependent variable arbitrarily [65]. Since each tree will have its own cutoffs, this makes the models very good at tackling non-linear problems. However, they cannot learn any partition beyond the range present in the training data. If the features point to a very high (low) value, then the trained model will predict the highest (lowest) value present in the training data. If so, the expectation is that Random Forest will perform the poorest when the highest or lowest values were not present in the training data. Indeed, this is what S3 Fig shows, and has been observed by others (e.g. [66, 67]): the only exception to the Random Forest under-performance was P6, for which the test period did not include the unusually intense 2018 flu outbreak. Model performance and full comparisons between observed and predicted cases for each period are provided in S4 Table and S2–S4 Figs in the Supporting information.

Critically, C1 consistently achieved the highest R^2 values across models, outperforming other feature sets in 4 out of 6 seasonal flu periods (Fig 2 A). The two exceptions were P2, where C1 and "All" performed equally well, and P6, where "All" terms performed better under linear regression (see S4 Table in the Supporting information). Unlike models trained on all search terms ("All", gray), which showed greater variability in accuracy across flu seasons, C1 produced more stable performance for seasonal flu cases. The performance of C2 (purple) was intermediate, with R^2 values generally lower than C1 but higher than C3 (green).

These results suggest that search term selection based on clustering during a pandemic period can enhance seasonal flu now-casting by reducing noise and improving performance stability. In contrast, using all available search terms introduces additional variance, likely capturing both relevant and irrelevant signals, which may lead to less robust seasonal predictions. Still, before the COVID-19 pandemic, flu outbreaks had clear seasonality and similar symptoms, and these results may not hold in the case of less repetitive contexts.

Applying flu-based feature selection to improve COVID-19 now-casting

While flu-related searches have historically shown consistency between seasonal and pandemic contexts, COVID-19 presented unique challenges that complicate now-casting efforts. These include: (1) the absence of a pre-existing surveillance infrastructure, such as the sentinel doctor networks available for flu; (2) uncertainty regarding whether COVID-19 will become a seasonal disease, as its outbreak periodicity remains unclear [68, 69]; (3) rapid and evolving changes in reporting practices, testing availability, and public health responses throughout the pandemic; and (4) the lack of a long historical dataset for model training. Therefore, the described relationship between pandemic-period search terms and disease incidence for the flu may not be observed in

the case of COVID-19.

Despite these challenges, we tested whether the feature selection approach applied to flu search terms could also improve COVID-19 now-casting (see Methods). We collected official case counts from March 2020 to May 2023, marking the beginning and end of the pandemic period. Instead of using predefined seasons (or waves) to train and test models, we applied a rolling window approach with 142 train/test splits (Fig 1G and H), including all possible break points.

We then assessed the accuracy of Linear Regression and Random Forest models trained using all search terms versus the clusters identified through hierarchical clustering (Fig 1E and F). Unlike the flu models, where we used a standard linear regression approach, for COVID-19 we implemented ARGO (AutoRegression with GOogle search data) [24], which incorporates search volume and cases from previous weeks as additional predictors. In this case, the Linear Regression models also outperformed the Random Forest, but likely for different reasons than in the case of the flu. Here, both models are equally poor at forecasting the largest peak (S5–S7 Figs in the Supporting information), but the Random Forest predictions are generally worse, while the Linear Regression fails less consistently. One possible explanation is the ARGO model using past predictions as a feature, while the Random Forest does not include such auto-correlation inputs.

The results (Fig 2C and S5–S6 Figs in the Supporting information) confirm that, as in the flu analysis, C1 search terms provided better predictions than using all search terms, C2, or C3 alone. These findings further support the idea that semi-manual feature selection can enhance the performance of now-casting models, particularly in distinguishing terms that better reflect disease dynamics.

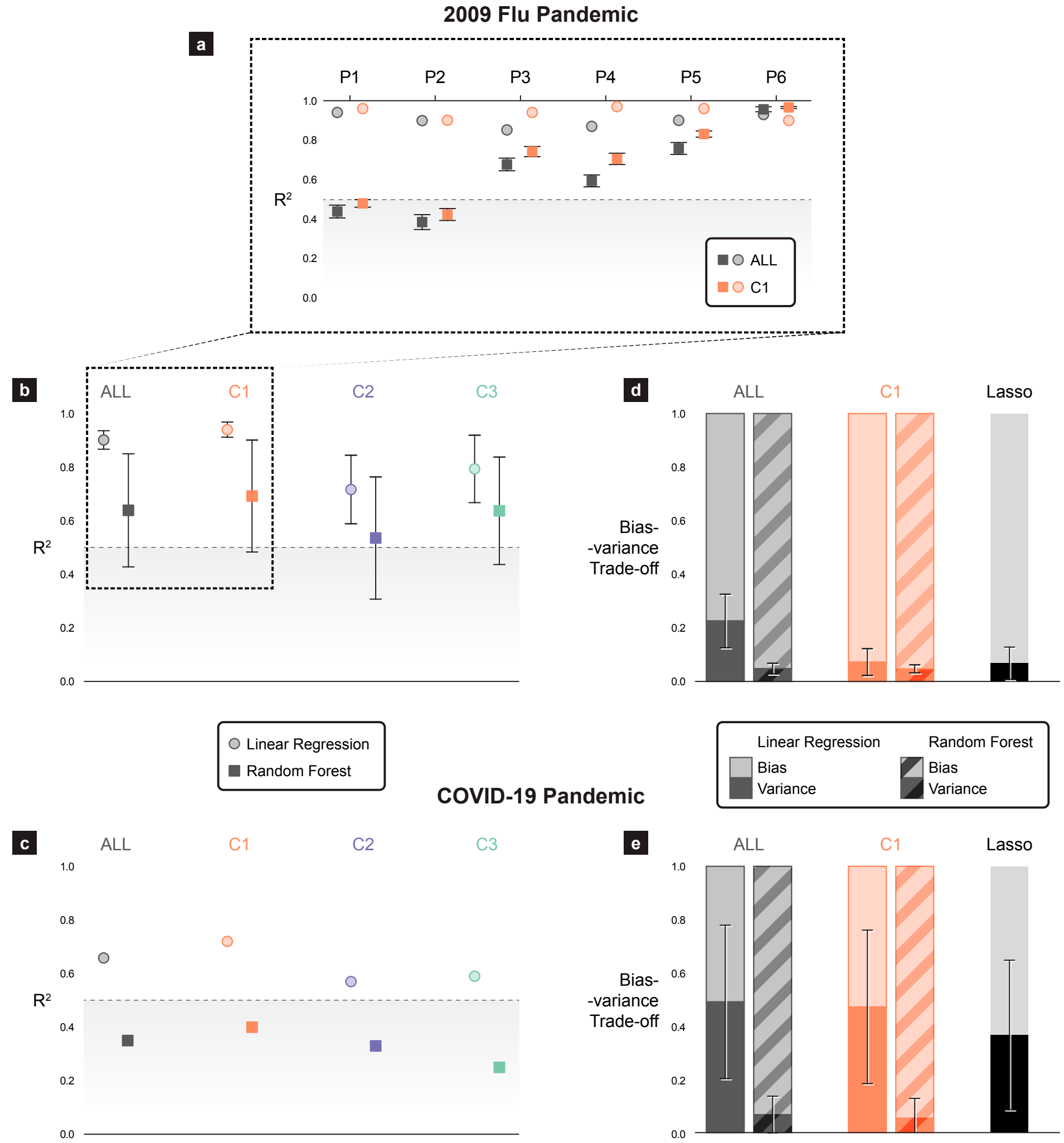

Fig 2. Comparison of now-casting model performance and bias-variance trade-off. Predictive performance of Linear Regression (circles) and Random Forest (squares) using different search-term clusters (All, C1, C2, C3) during the 2009 Flu and COVID-19 pandemics. Panels a), b), and d) correspond to flu, while c) and e) to COVID-19. For flu, error bars represent standard deviation across multiple seasons (P1–P6), reflecting model variability. Panel a) highlights C1's superior performance over "All". For COVID-19, standard deviations are not shown due to the rolling window approach, which results in overlapping training data and non-independent R^2 estimates. Instead, panel c) presents the R^2 of the mean prediction across models. Panels d) and e) illustrate the bias-variance trade-off. Random Forest has lower variance but slightly higher bias than Linear Regression. In flu (d), C1 reduces variance compared to "All", suggesting that selecting fewer, more relevant terms improves stability. In COVID-19 (e), bias remains high due to the pandemic's unpredictability. While here C1 also lowers variance relative to "All", this effect is weaker than in flu, where seasonal patterns allow for more consistent predictions. LASSO shows high bias and low variance.

Overfitting reduction, generalization, and predictive reliability in feature selection

The differences in performance observed between models using C1 search terms and those using all available terms are likely driven by overfitting: when including too many predictors, models learn patterns that do not generalize beyond the training data and exhibit high variance across different train/test splits [70, 71]. In contrast, using too few or poorly chosen features can lead to underfitting, where models miss meaningful relationships and display high bias. Managing the trade-off between reducing variance and introducing excessive bias, is essential to building robust models.To test this, we compared bias-variance trade-off of models trained on the terms from the C1 cluster versus those trained using all search terms (Fig 2D–E and S5–S6 Tables in the Supporting information).

Across both pandemics, bias tended to dominate variance, especially in the Linear Regression models, which is expected given their simplicity. Using all search terms increased variance compared to C1 for the flu, reinforcing the idea that removing less informative predictors stabilizes performance and suggesting that reducing feature count does not compromise performance but instead provides a more curated and stable feature set. As expected, Random Forest models consistently showed lower variance across conditions, regardless of the feature group used.

As choosing the right set of features is critical to balancing model complexity and predictive reliability, we further evaluated four alternative selection strategies (LASSO regression, correlation-based filtering, cluster size, and train-test split variability), also repeating the analysis by pandemic phase - temporal selection).

LASSO applies regularization by shrinking some coefficients to zero (L1), effectively performing feature selection by removing redundant terms. This typically reduces variance but can increase bias (Fig 2D and E). In both the flu and COVID-19 analyses, LASSO achieved performance levels comparable to C1 (see S9 Table in the Supporting information). However, most likely because LASSO selects terms based purely on statistical optimization, it led to inconsistent feature selection across the different flu seasons, (S8 Table). This variability reduces model interpretability, making it difficult to identify search terms that remain predictive over time.

LASSO regularization cannot be applied to the COVID-19 models in a straightforward way. Since we average the results of 142 models, and each model has its own ideal set of weights, it is unlikely any term would be removed from all models.

Second, and as C1 terms appeared to track infection trends well, we tested whether correlation-based selection could achieve similar results. The rationale is that similar results could have been achieved by simply identifying the search terms more correlated with the number of cases. We compared models using C1 and "All" pandemic-period terms to two correlation-based methods: (1) selecting the top 10% of search terms most correlated with flu cases during the pandemic period ($p < 0.05$), and (2) selecting the minimal number of terms per season needed to reach peak model performance during training.

The results show that selecting search terms based on correlation alone can yield strong predictive performance, at times exceeding C1 (see S11–S12 Tables in the Supporting information). This is consistent with recent work on dengue forecasting using Google Trends, where parsimonious search-term sets selected through statistical associations outperformed larger collections of search terms [72]. However, the effectiveness of this approach varied depending on whether selection was based on the pandemic period or adjusted for each period individually. When selecting the most correlated search terms during the pandemic period (five search terms), predictions remained strong but did not outperform seasonal selection or C1. For the seasonal selection of terms that achieve the peak model performance during training, the number

of predictors varied widely, from 34 search terms in one of the seasons to just one search term ("influenza a") in others.

This indicates that selecting features by correlation is highly sensitive to period-specific variation, suggesting that while pandemic periods help identify relevant search behaviors that are long-lasting, filtering by correlation alone does not ensure the best results in terms of performance or stability across different seasons.

Third, a simple explanation is that C1 has better results just because it includes fewer terms and this lowers model complexity. This is unlikely, as C1's size varies (smaller cluster in the case of the flu, largest in the case of COVID-19) yet systematically outperforms models trained with the other clusters (C2 and C3). Still, and to ensure that better predictive performance attributed to C1 was not simply due to the number of selected predictors, we compared performance, bias and variance of the C1 feature set against random selections of the same size. As S11 Table shows, across all flu seasons, C1 consistently outperformed models trained on random sets of nine terms. While some random selections performed well, their average R^2 was lower in every season (0.56–0.81 versus 0.90 - 0.97). Similarly, random models exhibited higher bias, indicating systematic underfitting, while C1 maintained a better bias / variance trade-off. In summary, C1 outperformed over 90% of random selections in 5 out of 6 seasons. Even in its weakest period, P6, it still performed better than 60% of random models, suggesting that any alternative high-performing sets emerge only by chance and lack interpretability. No random model using nine features matched or exceeded C1 across all seasons. We decided not to replicate the random feature control for COVID-19, as the C1 cluster in this case already included the largest number of terms, making it unlikely that its superior performance is due to model simplicity. Moreover, the flu analysis had already shown that random subsets of equal size rarely outperform behaviorally informed features, reducing the added value of repeating this analysis for COVID-19.

Fourth, we examined how train-test split variability affected model performance for COVID-19, focusing only on models trained with either the C1 feature set or all search terms. Unlike flu, where predefined seasonal periods provide a natural breakpoint structure, COVID-19 lacked clear seasonality, making model accuracy highly dependent on the specific division between training and testing data. In fact, performance varied substantially across different split points: some yielded strong predictions (R^2 ¿ 0.6), while others produced near-zero or even negative R^2 values (S6 Fig). Both C1 and "All" models followed similar trends, although the "All" models exhibited a slightly wider spread in performance, suggesting that including more search terms can enhance signal detection in some cases but also increase instability. We would like to emphasize this finding: we are aware that previous models have reached higher performance and we can replicate those results here; however, this high performance is too dependent on arbitrary choices and cannot be achieved systematically.

Finally, to further examine the temporal stability of model performance, we stratified the analysis by dominant COVID-19 variant phases (see S13–S14 Figs and S14 Table in the Supporting information). An increase in RMSE was observed during the Omicron BA.1 transition (winter 2021–2022), indicating a temporary decline in predictive performance coinciding with shifts in testing behavior and symptom perception. In parallel, a longitudinal evaluation of feature importance from the Random Forest models showed that symptom-specific terms such as *loss of smell* and *loss taste* significantly declined in predictive relevance after early 2022, whereas more general COVID-related queries (e.g., *covid cases*, *testing*) remained relatively stable. These findings suggest that changes in strain dominance coincide with changes in search patterns, emphasizing the importance of continuously incorporating behavioral dynamics into nowcasting frameworks.

Overall, our findings show that hierarchical clustering offers a practical and robust approach to feature selection. While LASSO and correlation-based methods may perform well locally, they produce inconsistent feature sets across time. C1, in contrast, offers a reliable balance between accuracy and stability. These are important qualities for models designed to operate in real-time surveillance contexts.

An *a posteriori* analysis of flu-related search terms shows that C1 mainly includes queries tied to illness perception and treatment-seeking, such as “flu symptoms,” “flu prevention,” and “flu treatment.” While similar terms appear in other clusters (e.g., “flu precautions” in C2, “flu safety” in C3), C1 is more consistently linked to self-diagnosis and prevention, suggesting that these searches are consistent with queries triggered by symptoms. For COVID-19, C1 mostly captures general symptoms, C2 includes societal-level terms, and C3 reflects testing and public health guidance. However, these interpretations are ad hoc: an *a priori* assessment would not necessarily anticipate the observed cluster structure and these clusters cannot be used to infer individual intent. Still, if certain search terms are more likely driven by personal illness and others by media or external cues, the clusters may reflect different underlying “real-world” behaviors.

To test this, we examined how each cluster correlates with confirmed case numbers and media mentions. The rationale being that if a cluster strongly correlates with case numbers, its terms are more likely to track actual disease burden, whereas a high correlation with media mentions suggests external information is shaping search behavior.

We collected media mentions of flu and COVID-19 over the same periods as case numbers and search trends (see Methods) and calculated their correlation with each search term cluster. Fig 3A and B shows that C1 exhibits the highest correlation with case numbers across both pandemics, whereas C2 is more strongly associated with media coverage. This pattern is further illustrated in S11 Fig in the Supporting information, where C1 closely follows case fluctuations while C2 aligns more with media mentions.

We previously showed that C1 offers greater stability in search term selection compared to using correlated terms alone (S11 Table).

Still, we identified the individual search terms more closely correlated with cases and media attention during the pandemic period, regardless of cluster, and examined their effect on predictive accuracy (Fig 3C and D). The left panel shows R^2 values for a linear regression model, adding search terms from most to least correlated with cases (left to right). The right panel shows R^2 values when adding search terms from least to most correlated with media coverage (right to left). Ranked search terms and R^2 cumulative values are provided in S12 Fig in the Supporting information.

Across both pandemics, model accuracy improves quickly when adding case-correlated terms but soon plateaus or declines, indicating that only a subset of terms contributes meaningfully to prediction, while additional terms introduce noise (likely due to an increase in variance as explained above). A similar pattern emerges for features correlated with media mentions (Fig 3C and D, right panel), where performance initially improves but quickly stabilizes or worsens as search terms more correlated with media are added, indicating that searches driven by media reduce model performance rather than improve it.

Looking at the order in which terms are added, we find that C3 terms (green) often appear early when ranked by case correlation, reflecting their link to general disease activity. However, these broad symptom searches (e.g., “fever,” “sore throat,” “runny nose”) offer limited predictive value on their own. They provide a baseline signal but do not drive peak performance without the inclusion of more targeted C1 terms, which appear to be more outbreak-specific. This trend is especially evident during the flu pandemic (top panels, P1 to P6), but also holds for COVID-19, where peak accuracy is

only achieved once C1 terms are incorporated. Additionally, these high levels of performance are reached with few or no C2 terms (purple), which are more closely associated with media coverage. This suggests that the clustering approach seems to effectively distinguish between behaviorally distinct search patterns, those driven by disease experience and those driven by external information, while filtering out less informative signals.

Discussion

This study aimed to develop a cost-effective and interpretable now-casting model using online search data. By applying hierarchical clustering to Google Trends during two pandemic periods (H1N1 and COVID-19), we identified consistent groups of search terms with distinct temporal dynamics. These clusters revealed that even seemingly similar search terms may be driven by different underlying motivations — some aligned with disease incidence, others with media influence or general interest. We present evidence that one of these clusters systematically outperforms more common purely statistical approaches, not only in terms of accuracy but also in terms of stability.

These findings contribute to a growing body of work that seeks to leverage digital traces, particularly search data, for real-time disease surveillance, while tackling two central challenges: 1) distinguishing meaningful signals from behavioral or media-driven noise, and 2) model overfitting and performance instability.

Our results demonstrate that behaviorally informed feature selection, especially when guided by temporal clustering, offers a promising path for improving the reliability and generalizability of now-casting models, reinforcing the key empirical lesson that more data is not always better. In fact, including all available features can introduce variance that overwhelms the true signal. Instead, our approach prioritizes coherence, identifying subsets of search terms that behave consistently during outbreaks (clusters) and remain predictive beyond crisis contexts. This aligns with recent calls for more interpretable, behavior-aware modeling strategies in digital health surveillance [73]. Importantly, we show that pandemic periods, often treated as outliers in epidemiological modeling, can function as natural experiments for feature selection. These periods amplify both disease-related and attention-related search activity, making it easier to differentiate terms that reflect disease from those driven by news cycles or societal anxiety. That these pandemic-identified terms (particularly those in C1) continue to outperform others across seasonal flu periods suggests they capture underlying behavioral regularities that are robust across time. This finding stands in contrast to purely statistical feature selection methods, such as correlation filtering or LASSO regression, which may perform well within narrow windows but often yield unstable and inconsistent results across datasets. Consistent with this view, the variant-stratified analysis highlights that model performance can shift with behavior and symptom frequency (e.g., declining value of loss of smell after Omicron). This also reinforces that feature selection alone does not guarantee temporal robustness; as search behaviour evolves, maintaining temporal robustness will require approaches that monitor changes in search patterns (e.g., feature drift) and rely on domain expertise for feature interpretation and validation.

In summary, this study offers a practical middle ground between high-dimensional, black-box models and rigid feature engineering. This is consistent with recent perspectives arguing that, in epidemiology, machine learning methods should be selected according to the question being addressed rather than solely on predictive performance [74]. Clustering based on temporal dynamics reduces dimensionality while preserving interpretability, a crucial balance in public health settings where decisions must be explainable and traceable. While both model types benefited, the advantages were especially clear for Linear Regression, a model class whose transparency makes it

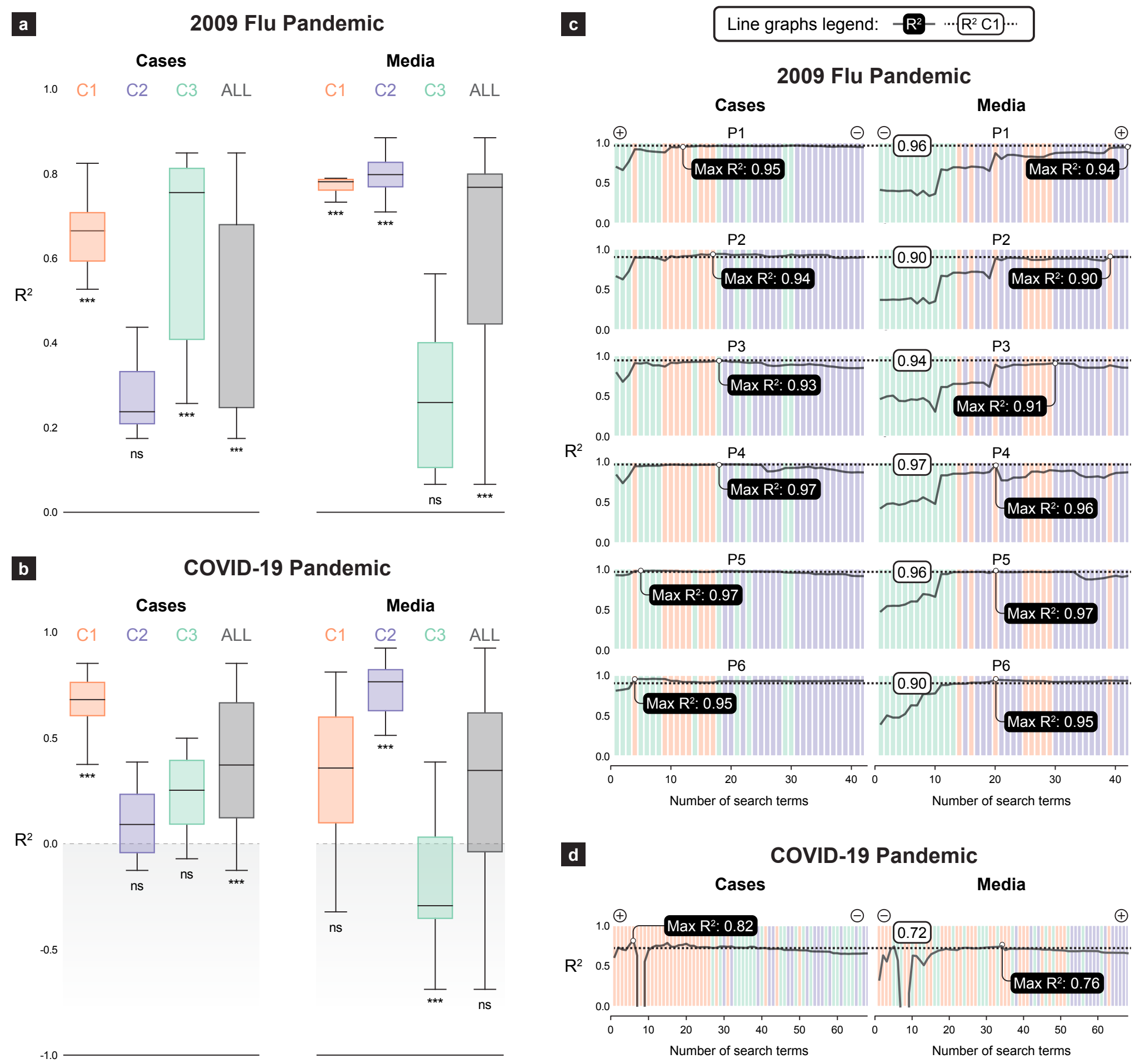

Fig 3. Correlation between search terms, infection cases, and media mentions. a) and b) show the Pearson correlation between each cluster and infection cases (left) and media mentions (right) during the pandemic period for both the flu (top) and COVID-19 (bottom). In contrast, c) and d) present the Linear Regression model's R^2 performance. The asterisks represent the p-value of a t-test with the null hypothesis that mean correlation is zero (*$0.01 < p < 0.05$; **$0.001 < p < 0.01$; ***$p < 0.0001$; ns $p > 0.05$). In a) and b), correlations can be both positive and negative, indicating whether search volumes increase or decrease with cases or media mentions. In c) and d), however, only the absolute correlation values are considered, as predictive power depends on correlation strength rather than direction. Also, c) and d) illustrates the cumulative contribution of individual search terms to the model's performance. Terms are added sequentially from the most to the least correlated with infection cases (left to right, in *Cases* panels) and from the least to the most correlated with media mentions (left to right, in *Media* panels). The black line represents the Linear Regression R^2, showing how predictive performance evolves as new terms are added, while the dashed line shows the R^2 obtained for C1 as a reference. We focus on the Linear Regression model here as it provides a more interpretable baseline for assessing the contribution of different feature sets. The colors of the vertical bars correspond to the clusters each term belongs to, allowing a comparison of cluster contributions.

particularly suitable for deployment in policy contexts. Importantly, as digital data grows in volume and heterogeneity, the risk of overfitting grows in parallel: prioritizing stable, interpretable features becomes increasingly relevant, particularly for emerging diseases with limited training data. By linking clusters to real-world signals such as case counts and media coverage, this approach supports more trustworthy, actionable machine learning in public health. Nevertheless, this method is not without limitations and we list five. First, while feature curation reduced variance and improved prediction for flu, the gains were more modest for COVID-19. This reflects a broader issue in digital surveillance: model performance is heavily dependent on the stability of underlying behaviors. In contexts with shifting testing protocols, public awareness, or policy environments, as seen in COVID-19, the mapping between search behavior and disease incidence becomes less predictable. This underscores the need for adaptive models that can recalibrate as behavioral and epidemiological baselines evolve (e.g., large scale vaccination, social distancing). Second, our clustering methodology relies on distinct temporal patterns (e.g., clear peaks), which may not exist for all outbreaks. In such cases, complementary approaches that incorporate additional contextual data, such as mobility patterns or syndromic surveillance, may enhance predictive performance. Third, while clustering reduces the inclusion of noisy predictors, it does not inherently infer causality. Some search terms may correlate with disease trends due to external factors rather than direct epidemiological relevance, as we saw with media mentions. Careful validation against traditional surveillance data remains essential to ensure reliability. Fourth, this study focused on a large country with broad access to online tools. Digital indicators may behave differently at finer spatial resolutions, and factors such as internet access, digital behavior, and public health communication strategies can influence the reliability of using search-terms in different geographic contexts. However, the inexpensive nature and scalability of this method may make it particularly useful in low-resource settings where traditional disease surveillance is limited [73]. Indeed, if deployed responsibly, this method also offers promise to help reduce health inequities by providing rapid insights. Curated search terms, if adjusted for local behaviors and disparities in digital access, can improve disease surveillance without reinforcing existing digital divides. Future research might assess the effectiveness of this approach across diverse geographic and socio-economic contexts. Finally, for public health use, analysis must remain consistent and reproducible. A method that relies on private platform data, as is the case of Google Trends and its API, risks interruptions or instability from changing access paths, shifting policies, quota limits, pricing, and even basic availability. Although interface sampling can introduce minor run-to-run variation, it can minimize some of these issues. In this work, repeated exports show that comparative results remain stable (see S8–S9 Figs in the Supporting information), and preference should be given to pipelines resilient to platform variability. Still, they do not solve the fundamental problem of relying on non-public data. Overall, future work should explore the integration of additional data sources, such as mobility data, syndromic surveillance, and social media signals, to enhance robustness and generalizability.

In sum, this study demonstrates that thoughtful, behaviorally grounded feature selection can improve the robustness, stability, and transparency of digital now-casting systems, and expanding this framework to real-time monitoring could further support public health decision-making, for both seasonal and emerging infectious diseases.

Supporting information

S1 Fig. Stability of clustering and centroid patterns across independent Google Trends data extractions.

S2 Fig. Predicted vs. observed cases for the 2009 Flu pandemic (Linear Regression).

S3 Fig. Predicted vs. observed cases for the 2009 Flu pandemic (Random Forest).

S4 Fig. Residual distribution for the flu predictions.

S5 Fig. Predicted vs. observed cases for the COVID-19 pandemic (Linear Regression).

S6 Fig. Predicted vs. observed cases for the COVID-19 pandemic (Random Forest).

S7 Fig. Residual distribution for the COVID-19 predictions.

S8 Fig. Flu reproducibility: Pytrends versus official Google Trends exports.

S9 Fig. COVID-19 reproducibility: Pytrends versus official Google Trends exports.

S10 Fig. Distribution of R^2 across 142 rolling train–test splits.

S11 Fig. Media mentions, cases, and search-term clusters over time (Flu and COVID-19).

S12 Fig. Ranking of search terms by correlation with cases and media mentions.

S13 Fig. Temporal instability across COVID-19 variants.

S14 Fig. Temporal evolution of Random Forest feature importance across variant phases.

S1 Table. Search term clusters for the Flu pandemic.

S2 Table. Search term clusters for the COVID-19 pandemic.

S3 Table. Training and test period splits for seasonal prediction using Flu-period terms.

S4 Table. R^2 values for Linear Regression and Random Forest (Flu and COVID-19).

S5 Table. Bias–Variance trade-off for Flu.

S6 Table. Bias–Variance trade-off for COVID-19.

S7 Table. Reproducibility of Google Trends extraction for Flu (pytrends vs. official exports).

S8 Table. Reproducibility of Google Trends extraction for COVID-19 (pytrends vs. official exports).

S9 Table. Lasso Regression performance across periods (P1–P6).

S10 Table. Lasso weights per search term across periods (clustered by C1–C3).

S11 Table. Random feature selection (9 features) vs. C1-only across seasons.

S12 Table. Performance when selecting terms by correlation with cases (pandemic period).

S13 Table. Performance when selecting terms by seasonal correlation (P1–P6).

S14 Table. Mean RMSE and R^2 by dominant COVID-19 variant phase (Linear Regression models).

Acknowledgments

We thank the members of the Social Physics and Complexity (SPAC) group at LIP for their comments and critical reading of the manuscript.

References

1. Hay SI, George DB, Moyes CL, Brownstein JS. Big data opportunities for global infectious disease surveillance. PLoS med. 2013;10(4):e1001413.

2. Ferretti L, Wymant C, Kendall M, Zhao L, Nurtay A, Abeler-Dörner L, et al. Quantifying SARS-CoV-2 transmission suggests epidemic control with digital contact tracing. Science. 2020;368(6491).

3. Salathe M, Bengtsson L, Bodnar TJ, Brewer DD, Brownstein JS, Buckee C, et al. Digital epidemiology. PLoS Comput Biol. 2012;8(7):e1002616.

4. Du H, Zhao Y, Zhao J, Xu S, Lin X, Chen Y, et al. Advancing real-time infectious disease forecasting using large language models. Nature Computational Science. 2025;5(6):467-80.

5. Fox S. Online health search 2006. Pew Internet & American Life Project; 2006.

6. Gluskin RT, Johansson MA, Santillana M, Brownstein JS. Evaluation of Internet-based dengue query data: Google Dengue Trends. PLoS neglected tropical diseases. 2014;8(2):e2713.

7. Chan EH, Sahai V, Conrad C, Brownstein JS. Using web search query data to monitor dengue epidemics: a new model for neglected tropical disease surveillance. PLoS neglected tropical diseases. 2011;5(5):e1206.

8. Althouse BM, Ng YY, Cummings DA. Prediction of dengue incidence using search query surveillance. PLoS Negl Trop Dis. 2011;5(8):e1258.

9. Husnayain A, Fuad A, Lazuardi L. Correlation between Google Trends on dengue fever and national surveillance report in Indonesia. Global Health Action. 2019;12(1):1552652.

10. Chen Y, Zhang Y, Xu Z, Wang X, Lu J, Hu W. Avian Influenza A (H7N9) and related Internet search query data in China. Scientific reports. 2019;9(1):10434.

11. Teng Y, Bi D, Xie G, Jin Y, Huang Y, Lin B, et al. Dynamic forecasting of Zika epidemics using Google trends. PloS one. 2017;12(1):e0165085.

12. Google Flu Trends. Google Flu Trends [Internet]. Google.org; 2013 [cited 2020 Dec 22]. Available from: `https://web.archive.org/web/20121022154915/http://www.google.org/flutrends/about/how.html`.

13. Ginsberg J, Mohebbi MH, Patel RS, Brammer L, Smolinski MS, Brilliant L. Detecting influenza epidemics using search engine query data. Nature. 2009;457(7232):1012-4.

14. Won M, Marques-Pita M, Louro C, Gonçalves-Sá J. Early and real-time detection of seasonal influenza onset. PLoS computational biology. 2017;13(2):e1005330.

15. De Toni G, Consonni C, Montresor A. A general method for estimating the prevalence of influenza-like-symptoms with Wikipedia data. Plos one. 2021;16(8):e0256858.

16. Liu J, Suzuki S. Real-Time Detection of Flu Season Onset: A Novel Approach to Flu Surveillance. International Journal of Environmental Research and Public Health. 2022;19(6):3681.

17. Morris M, Hayes P, Cox IJ, Lampos V. Neural network models for influenza forecasting with associated uncertainty using Web search activity trends. PLoS Computational Biology. 2023;19(8):e1011392.

18. Ning S, Hussain A, Wang Q. Incorporating connectivity among Internet search data for enhanced influenza-like illness tracking. Plos one. 2024;19(8):e0305579.

19. Princess PJB, et al. LLM-Assisted Keyword Curation for Early Disease Outbreak Detection Using Search Trends. In: 2025 4th International Conference on Applied Artificial Intelligence and Computing (ICAAIC). IEEE; 2025. p. 478-83.

20. Lazer D, Kennedy R, King G, Vespignani A. The parable of Google Flu: traps in big data analysis. Science. 2014;343(6176):1203-5.

21. Towers S, Afzal S, Bernal G, Bliss N, Brown S, Espinoza B, et al. Mass media and the contagion of fear: the case of Ebola in America. PloS one. 2015;10(6):e0129179.

22. Olson DR, Konty KJ, Paladini M, Viboud C, Simonsen L. Reassessing Google Flu Trends data for detection of seasonal and pandemic influenza: a comparative epidemiological study at three geographic scales. PLoS Comput Biol. 2013;9(10):e1003256.

23. Tizzoni M, Panisson A, Paolotti D, Cattuto C. The impact of news exposure on collective attention in the United States during the 2016 Zika epidemic. PLoS computational biology. 2020;16(3):e1007633.

24. Yang S, Santillana M, Kou SC. Accurate estimation of influenza epidemics using Google search data via ARGO. Proceedings of the National Academy of Sciences. 2015;112(47):14473-8.

25. Mena I, Nelson MI, Quezada-Monroy F, Dutta J, Cortes-Fernández R, Lara-Puente JH, et al. Origins of the 2009 H1N1 influenza pandemic in swine in Mexico. Elife. 2016;5:e16777.

26. Brammer L, Blanton L, Epperson S, Mustaquim D, Bishop A, Kniss K, et al. Surveillance for influenza during the 2009 influenza A (H1N1) pandemic–United States, April 2009–March 2010. Clinical Infectious Diseases. 2011;52(suppl_1):S27-35.

27. Devaux I, Kreidl P, Penttinen P, Salminen M, Zucs P, Ammon A, et al. Initial surveillance of 2009 influenza A (H1N1) pandemic in the European Union and European economic area, April–September 2009. Eurosurveillance. 2010;15(49):19740.

28. Fox SJ, Miller JC, Meyers LA. Seasonality in risk of pandemic influenza emergence. PLoS computational biology. 2017;13(10):e1005749.

29. World Health Organization. Pandemic (H1N1) 2009 – update 112. World Health Organization; 2010. Accessed: YYYY-MM-DD. Available from: `https://iris.who.int/bitstream/handle/10665/78414/9789241503051_eng.pdf?sequence=1`.

30. Dawood FS, Iuliano AD, Reed C, Meltzer MI, Shay DK, Cheng PY, et al. Estimated global mortality associated with the first 12 months of 2009 pandemic influenza A H1N1 virus circulation: a modelling study. The Lancet infectious diseases. 2012;12(9):687-95.

31. World Health Organization. WHO COVID-19 Dashboard: Deaths [Internet]. World Health Organization; 2025 [cited 2025 Feb 11]. Available from: `https://data.who.int/dashboards/covid19/deaths`.

32. Panning M, Eickmann M, Landt O, Monazahian M, Ölschläger S, Baumgarte S, et al. Detection of influenza A (H1N1) v virus by real-time RT-PCR. Eurosurveillance. 2009;14(36):19329.

33. Teymouri M, Mollazadeh S, Mortazavi H, Ghale-Noie ZN, Keyvani V, Aghababaei F, et al. Recent advances and challenges of RT-PCR tests for the diagnosis of COVID-19. Pathology-Research and Practice. 2021.

34. Rosen JM, Warzecha HR. Coronavirus Variant Emergence and Epidemiology. In: The COVID-19 Pandemic: Science, Technology, and the Future of Healthcare Delivery. Springer; 2024. p. 193-206.

35. Wang Y, Tang CY, Wan XF. Antigenic characterization of influenza and SARS-CoV-2 viruses. Analytical and Bioanalytical Chemistry. 2022;414(9):2841-81.

36. World Health Organization. Pandemic influenza vaccine manufacturing process and timeline [Internet]. World Health Organization; 2009 [cited 2025 Feb 11]. Available from: `https://www.who.int/news/item/06-08-2009-pandemic-influenza-vaccine-manufacturing-process-and-timeline`.

37. King ML. How manufacturing won or lost the COVID-19 vaccine race. Vaccine. 2024.

38. Da Costa VG, Saivish MV, Santos DER, de Lima Silva RF, Moreli ML. Comparative epidemiology between the 2009 H1N1 influenza and COVID-19 pandemics. Journal of infection and public health. 2020;13(12):1797-804.

39. Wasim A, Truong J, Bakshi S, Majid U. A systematic review of fear, stigma, and mental health outcomes of pandemics. Journal of Mental Health. 2023;32(5):920-34.

40. Peláez-Ballestas I, Infante-Castañeda C, Giraldo-Rodríguez L. Comparison between Covid-19 and influenza A (H1N1) pandemic experiences and risk perception in a Mexican university. salud pública de méxico. 2021;63(5):619-29.

41. Kogan NE, Clemente L, Liautaud P, Kaashoek J, Link NB, Nguyen AT, et al. An early warning approach to monitor COVID-19 activity with multiple digital traces in near real-time. arXiv preprint arXiv:200700756. 2020.

42. Dewhurst DR, Alshaabi T, Arnold MV, Minot JR, Danforth CM, Dodds PS. Divergent modes of online collective attention to the COVID-19 pandemic are associated with future caseload variance. arXiv preprint arXiv:200403516. 2020.

43. Liu D, Clemente L, Poirier C, Ding X, Chinazzi M, Davis JT, et al. A machine learning methodology for real-time forecasting of the 2019-2020 COVID-19 outbreak using Internet searches, news alerts, and estimates from mechanistic models. arXiv preprint arXiv:200404019. 2020.

44. Ayyoubzadeh SM, Ayyoubzadeh SM, Zahedi H, Ahmadi M, Kalhori SRN. Predicting COVID-19 incidence through analysis of google trends data in iran: data mining and deep learning pilot study. JMIR Public Health and Surveillance. 2020;6(2):e18828.

45. Lu T, Reis BY. Internet Search Patterns Reveal Clinical Course of Disease Progression for COVID-19 and Predict Pandemic Spread in 32 Countries. medRxiv. 2020.

46. Effenberger M, Kronbichler A, Shin JI, Mayer G, Tilg H, Perco P. Association of the COVID-19 pandemic with internet search volumes: a google trendstm analysis. International Journal of Infectious Diseases. 2020.

47. Grasso KL, Bell RA. Understanding health information seeking: A test of the risk perception attitude framework. Journal of health communication. 2015;20(12):1406-14.

48. Weaver III JB, Mays D, Weaver SS, Hopkins GL, Eroğlu D, Bernhardt JM. Health information–seeking behaviors, health indicators, and health risks. American journal of public health. 2010;100(8):1520-5.

49. Hone T, Palladino R, Filippidis FT. Association of searching for health-related information online with self-rated health in the European Union. The European Journal of Public Health. 2016;26(5):748-53.

50. Link E, Baumann E, Klimmt C. Explaining online information seeking behaviors in people with different health statuses: German representative cross-sectional survey. Journal of medical Internet research. 2021;23(12):e25963.

51. Li J, Zheng H. Online information seeking and disease prevention intent during COVID-19 outbreak. Journalism & Mass Communication Quarterly. 2022;99(1):69-88.

52. Kamalov F, Sulieman H, Alzaatreh A, Emarly M, Chamlal H, Safaraliev M. Mathematical methods in feature selection: A review. Mathematics. 2025;13(6):996.

53. Li Y, Li T, Liu H. Recent advances in feature selection and its applications. Knowledge and Information Systems. 2017;53(3):551-77.

54. FluNet;. Accessed: 2020-06-18. https://www.who.int/influenza/gisrs_laboratory/flunet/en/.

55. Our World in data;. Accessed: 2020-08-20. https://github.com/owid/covid-19-data/tree/master/public/data.

56. Google Trends. Google Trends; 2025. Accessed: 2025-02-10. Available from: https://trends.google.com/trends/.

57. Sood G, Laohaprapanon S. Vanderbilt TV News Abstracts. Harvard Dataverse; 2020. Available from: https://doi.org/10.7910/DVN/BP2JXU. doi:10.7910/DVN/BP2JXU.

58. Cloud M. Media Cloud: Global News Database; 2023. Accessed: 2023-05-15. https://search.mediacloud.org/search.

59. SciPy Community. SciPy: Scientific Computing Tools for Python; 2023. Accessed: 2025-02-09. Available from: https://scipy.org.

60. LinearRegression;. https://scikit-learn.org/stable/modules/generated/sklearn.linear_model.LinearRegression.htmll.

61. RandomForestRegressor;. https://scikit-learn.org/stable/modules/generated/sklearn.ensemble.RandomForestRegressor.html.

62. Raschka S. Mlxtend: A library of Python tools and extensions for data science; 2023. Version retrieved: 2023-05-15. Available from: https://pypi.org/project/mlxtend/.

63. Ma KC. Genomic surveillance for SARS-CoV-2 variants: circulation of omicron lineages—United States, January 2022–May 2023. MMWR Morbidity and mortality weekly report. 2023;72.

64. Benjamini Y, Hochberg Y. Controlling the false discovery rate: a practical and powerful approach to multiple testing. Journal of the Royal statistical society: series B (Methodological). 1995;57(1):289-300.

65. Breiman L, Friedman J, Olshen RA, Stone CJ. Classification and regression trees. Chapman and Hall/CRC; 2017.

66. Meyer H, Pebesma E. Predicting into unknown space? Estimating the area of applicability of spatial prediction models. Methods in Ecology and Evolution. 2021;12(9):1620-33.

67. Hengl T, Nussbaum M, Wright MN, Heuvelink GB, Gräler B. Random forest as a generic framework for predictive modeling of spatial and spatio-temporal variables. PeerJ. 2018;6:e5518.

68. Wiemken TL, Khan F, Puzniak L, Yang W, Simmering J, Polgreen P, et al. Seasonal trends in COVID-19 cases, hospitalizations, and mortality in the United States and Europe. Scientific reports. 2023;13(1):3886.

69. Quinn GA, Connolly M, Fenton NE, Hatfill SJ, Hynds P, Ohaiseadha C, et al. Influence of seasonality and public-health interventions on the COVID-19 pandemic in northern Europe. Journal of Clinical Medicine. 2024;13(2):334.

70. Doupe P, Faghmous J, Basu S. Machine learning for health services researchers. Value in Health. 2019;22(7):808-15.

71. Lever J, Krzywinski M, Altman N. Points of significance: model selection and overfitting. Nature methods. 2016;13(9):703-5.

72. Cheong KH, Li K, Yu D, Zhao X. Forecasting dengue cases through time-series modeling with Google Trends and deep neural networks. Chaos, Solitons & Fractals. 2025;201:117290.

73. Mesquita S, Perfeito L, Paolotti D, Gonçalves-Sá J. Epidemiological methods in transition: Minimizing biases in classical and digital approaches. PLOS Digital Health. 2025;4(1):e0000670.

74. Atias D, Ashri S, Goldbourt U, Benyamini Y, Gilad-Bachrach R, Hasin T, et al. Machine learning in epidemiology: An introduction, comparison with traditional methods, and a case study of predicting extreme longevity. Annals of Epidemiology. 2025.

We thank the Academic Editor and the reviewers for the time and effort invested in the review process and for their relevant comments and suggestions. We have carefully considered all comments and have revised the manuscript accordingly. Below, in blue, we provide a detailed, point-by-point response to each comment. Changes made to the manuscript are indicated in italics throughout this document and in blue in the manuscript.

Reviewer 3

The paper titled "Learning from Pandemics: Feature Selection for Robust Digital Disease Nowcasting" requires essential improvements. Some sections of the present version of the revision remained unclear and then reviewer is worrying about scientific and originality of the paper contents.

We thank R3 for the detailed comments and while we agree with several suggestions we respectfully try to justify why some others might be outside of the scope of the current work.

1-In abstract section, authors need to mention which Machine Learning Models (MLMs) were used in this study with their main purposes. The main findings along with its conditions of datasets can be merged to the abstract section.

To comply both with the reviewer's request and with the journal's word formatting requirements, the following sentence has been added to the abstract (in italics):

"We propose a hybrid approach to improve prediction that combines model evaluation with behavioral insight. *We apply simple supervised and unsupervised methods to different* flu and COVID-19 *datasets*, using the 2009 H1N1 and 2020 COVID-19 pandemics as training periods. In both cases, selecting a curated subset of search terms improved model performance, reduced overfitting, and increased stability, consistently outperforming models that used search terms indiscriminately."

2-Introduction section requires diverse related research works, describing application of MLMs in the case of feature selection and capability of robust MLMs. Reviewer is now considering table containing literature review in this area whereas this table need a lot of major modifications.

For Feature Selection:
-Unsupervised feature selection via graph-based proximity and structured autoencoder-like NMF
-Joint sample-feature subspace learning via bidirectional reconstruction for feature selection
For robustness:
-Robust oblique projection and weighted NMF for hyperspectral unmixing
-Simultaneous outlier detection and elimination in hyperspectral unmixing via weighted non-negative matrix tri-factorization

We thank the reviewer for these suggestions and carefully considered the recommended references. While some of these articles fall outside the scope of the present study, as the manuscript focuses on the application of machine learning methods to digital epidemiology and disease nowcasting rather than on methodological developments in machine learning algorithms, we see how the paper could benefit from a broader overview of recent work in this area. Accordingly, we have updated it with references covering (i) applications of machine learning in epidemiology, (ii) recent developments in feature selection methodologies, and (iii) recent advances in infectious disease forecasting using digital data. In particular, we have added:

1 - LLM-Assisted Keyword Curation for Early Disease Outbreak Detection Using Search Trends, by Brahmos and Princess (2025), which reinforces the importance of careful feature selection in search-based epidemiology and provides a complementary, LLM-assisted approach to keyword curation.

Added to the Introduction (Lines 19-23): "*As the use of online search data for disease surveillance has matured, attention has shifted from whether these data are useful to how the most informative search terms should be selected. Even with recent advances in artificial intelligence, careful keyword curation remains essential for identifying the search terms that carry the most relevant information for disease prediction* [19]."

2 - "Machine learning in epidemiology: An introduction, comparison with traditional methods, and a case study of predicting extreme longevity", a recent review by Goldstein et al., which discusses the application of machine learning in epidemiology and reinforces the importance of selecting methods according to the epidemiological question being addressed

Added to the Discussion (Lines 663–665): "*This is consistent with recent perspectives arguing that, in epidemiology, machine learning methods should be selected according to the question being addressed rather than solely on predictive performance* [74]."

3 – "Mathematical methods in feature selection: A review", by Kamalov et al. (2025), which classifies feature selection methods according to their mathematical foundations and discusses their respective strengths, limitations, and areas of application, providing additional context for the approaches evaluated in the present study.

4 – "Recent advances in feature selection and its applications", by Li et al., which reviews recent developments in feature selection and highlights the importance of selecting relevant and non-redundant features to improve both predictive performance and model interpretability. This provides additional theoretical context for the behaviourally informed feature selection strategy proposed in the present study.

Citations 3 and 4 were added to the Introduction (Lines 88—90): "*Recent reviews have emphasized that effective feature selection should identify informative predictors while reducing redundancy, thereby improving predictive performance, robustness, and model interpretability* [52, 53]."

5 - Advancing real-time infectious disease forecasting using large language models, by Du et al. (2025), which provides a recent overview of advances in infectious disease forecasting using artificial intelligence and highlights the growing integration of digital and heterogeneous data sources to complement traditional surveillance systems.

Added to the Introduction (Lines 7--10): "*As surveillance mechanisms relying solely on highly trained personnel are often expensive, limited, and slow, it has been extensively argued that traditional methods should be complemented with 'Digital Era' tools, such as online activity, mobility patterns, or digital contact-tracing* [1, 2, 3, 4]."

6 - Forecasting dengue cases through time-series modeling with Google Trends and deep neural networks, by Cheong et al. (2025), which demonstrates that parsimonious Google Trends feature sets can outperform larger collections of search terms in infectious disease forecasting. While their feature selection is based on statistical associations,

our work provides a complementary behavioural perspective that helps explain why the indiscriminate inclusion of search terms may reduce predictive performance.

Added to the Results (Lines 507--509): "*This is consistent with recent work on dengue forecasting using Google Trends, where parsimonious search-term sets selected through statistical associations outperformed larger collections of search terms* [72].*"*

3-Research gap and novelty should be deeply discussed in the introduction section.

We agree that clearly defining the research gap and novelty is important. To address this comment, we revised the final paragraph of the Introduction to more clearly distinguish our contribution from previous work. Specifically, we now explicitly identify the research gap and clarify the novelty of the proposed approach. The Introduction now reads:

"*Recent reviews have emphasized that effective feature selection should identify informative predictors while reducing redundancy, thereby improving predictive performance, robustness, and model interpretability. However, relatively little attention has been paid to whether pandemic periods themselves can be exploited to improve feature selection. To address this gap, we propose a simple and explainable feature selection framework that leverages the high-intensity periods of pandemics as natural experiments to identify search terms with greater temporal robustness for disease nowcasting*."(Lines 88–95).

4-Research organization, known as the last paragraph of introduction section should be deeply provided.

To address this comment, we expanded the final paragraph of the Introduction to include a brief overview of the organization of the manuscript. After introducing the research gap and the proposed method, we now briefly describe how the study is structured, from the presentation of the data sources and modelling approach to the evaluation of the proposed method and the discussion of its implications.

The Introduction now concludes with:

"To evaluate this approach, we first describe the data sources and modelling approach, then assess its performance across influenza and COVID-19 case studies, and finally discuss its implications for the design of robust and interpretable disease nowcasting systems." (Lines 98-101)

5- Delete subsections in the introduction section. And more, all the subsections should have had only single name as "Introduction" in which includes (i) importance of subject and background theorem, (ii) literature review with emphasize on major drawbacks of empirical/traditional methodologies equations, (iii) research gap and novelty, and (iv) research organization in which overview of the present research should be mentioned in a paragraph.

We ask the reviewer to confirm whether the current version of the manuscript complies with that request or offer more detail on how it should be improved.

6-Details of case study and some important discussions on the datasets should be detailed.

We ask the reviewer to confirm whether the current version of the manuscript complies with that request or offer more detail on how it should be improved.

7-Authors are emphatically recommended to use MLMs such as KNN, Model Tree (MT), AdaBoost, XGBoost, CatBoost, LightBoost, and RF for the training and testing stages.

Algorithms such as KNN, Model Trees, AdaBoost, XGBoost, CatBoost, and LightGBM are indeed powerful machine learning methods and can provide great predictive performance in many applications. However, since the primary objective of the present study was to evaluate whether behaviourally informed feature selection improves the robustness and generalizability of disease nowcasting models, we decided against testing a broad range of models and selected two complementary and widely used approaches: Linear Regression, as a simple and fully interpretable baseline, and Random Forest, as a nonlinear ensemble method capable of capturing more complex relationships.

The fact that the proposed feature selection strategy consistently improved performance across both model classes suggests that the observed gains arise from the quality of the selected features and not from a particular algorithm. While evaluating additional algorithms such as gradient boosting methods would certainly be an interesting direction for future work, we respectfully argue that it falls outside the scope of the present study and would not substantially alter its main conclusions.

9-Descriptions on every MLM should be presented in a separate subsection along with the setting parameters of each predictive models and the corresponding justifications. In this section, authors should use AdaBoost, CatBoost, LighGBoost, XGBoost, KNN, SVM, RF, and other regression models based on machine learning techniques (ElasNet, Lasso, and Ridge).

To improve this section, authors need to use the material of the following papers:

- Vulnerability of the rip current phenomenon in marine environments using machine learning models

- Assessment of flood susceptibility in Minab County, Iran, through the integration of topographic, climatic, and land-surface indices using ensemble machine learning models

- Ultrasonic flowmeter fault early warning method based on Bayesian optimization XGBoost

- Evaluation of river water quality index using remote sensing and artificial intelligence models

- A Stacked Machine Learning Algorithm for Multi-Step Ahead Prediction of Soil Moisture

Please see our response to Comment 7 regarding the rationale for the selection of the predictive models evaluated in this study, as the same considerations apply here.

10- Instead using R^2, authors are emphatically recommended to use Correlation of Coefficient (CC), RMSE, Mean Absolute Error (MAE), Mean Bias Error (MBE), Discrepancy Ratio (DR), and Scatter Index (SI) to this section. These statistical measures should be computed for the training and testing stages of the present predictive models. All the formulations and acceptable ranges of these statistical measures should be merged to this section. For this purpose, read the following papers and add them to the present revisions.

-A hybrid hourly natural gas demand forecasting method based on the integration of wavelet transform and enhanced Deep-RNN model

-A deep reinforcement learning-based method for predictive management of demand response in natural gas pipeline networks

-A Combinatorial Machine Learning Models -Based Method for Predicting the Viscosity-Temperature Relationship of Crude Oil

-Receiving More Accurate Predictions for Longitudinal Dispersion Coefficient in Water Pipelines: Training Group Method of Data Handling Using Extreme Learning Machine Conceptions

-A Method for Predicting the Gel Point of Mixed Crude Oil Based on Deep Learning

- A Large Language Model Based Knowledge Mining Method for Improving the Reliability of Fire Water Systems

- Optimization Method of Natural Gas Pipeline Network Emergency Dispatch Based on Particle Swarm Optimization

-Data-driven reliability assessment method of Integrated Energy Systems based on probabilistic deep learning and Gaussian mixture Model-Hidden Markov Model

-Interpretable Combinatorial Machine Learning-Based Shale Fracability Evaluation Methods

-Dynamic Prediction of Natural Gas Calorific Value Based on Deep Learning

-Water quality monitoring for coastal hypoxia: Integration of satellite imagery and machine learning models

-Dynamic damage functions for scour protection at mono-pile foundations: Application of ensemble machine learning models

-A new correlation for calculating carbon dioxide minimum miscibility pressure based on multi-gene genetic programming

We ask the reviewer to confirm whether the current version of the manuscript complies with that request. In addition to R2, and partially also in response to reviewer R1, we added bias-variance decomposition (Methods, Bias-variance trade-off subsection), and RMSE-based analyses, including phase-specific and rolling RMSE evaluations (Methods, Variant-stratified evaluation subsection; Results, Overfitting reduction, generalization, and predictive reliability in feature selection subsection; S14 Table).

11-Use Scatter plots, Taylor's diagrams, heat maps, and violin plots to illustrate the results of the training and testing stages.

We agree that visual assessment is very useful when evaluating models. In the current manuscript and Supporting Information, we already provide several complementary visualizations of model performance, including line plots comparing observed and predicted trajectories for both Linear Regression and Random Forest models in the flu and COVID-19 analyses (S2–S3 and S5–S6 Figs), scatter plots relating residuals to observed incidence, line plots showing the temporal evolution of residuals, histograms illustrating residual and incidence distributions, and binned summary plots of mean residuals across incidence levels (S4 and S7 Figs). In the main manuscript, we also compare model performance across feature sets (Fig. 2). In addition, the manuscript includes heatmaps illustrating the longitudinal evolution of feature importance across SARS-CoV-2 variant periods, as requested by another reviewer.

It is our assessment that the current set of figures provides sufficient visual information to evaluate model performance and prediction errors across the training and testing settings. While Taylor diagrams and violin plots could provide an alternative way of visualizing some of these results, we are not sure that they would provide additional insight. If the reviewer feels that a particular aspect of model performance would benefit from additional visualization, we would appreciate more specific clarification so that we can better understand the concern and address it as effectively as possible.

12-The present results should be compared with those related papers (use more than 15 papers at least with quantitative and qualitative comparisons).

Please see answer to comment 2.

Reviewer #6:

Based on the manuscript contents and contexts, all the above questions seem to fulfill the PLOS requirements.

We thank the reviewer for the positive and constructive comments and overall recommendation to publish.

However, there are a few comments to the authors for consideration, which include:

1. The manuscript title is "Learning from Pandemics: Feature Selection for Robust Digital Disease Nowcasting." Discussions are focused on how to improve prediction that combines model evaluation with behavioral insight using curated features. Since hierarchical clustering is applied in data processing and the clustering results (C1, C2, C3) become the input for the rest of the analysis for 2009 H1N1, while for Covid-19 the predefined period (P1, P2, ..., P6) is applied, I would suggest revising the title to use "Feature Clustering" instead. For the "Feature Selection" to be used, discussions shall highlight how the number of searched terms is reduced in quantity (leaving only the significant terms) that would help improve the prediction. Consider this if it is viable.

We thank the reviewer for the comment. It is not only viable, but it also offers a better description of the work. We have updated the title as suggested.

2. Searched terms in the online may be an intercept between different aspects of symptoms, treatment, personal care, institutions, and pandemic-specific terms. As this led to redundancy, how does this study see this as a threat to the accuracy of nowcasting? Add your comments in the discussion if it has not been addressed.

We agree both with the note that search terms can have different natures and underlying motivations, and that redundancy is often a problem. We cluster similar terms and include two types of analysis (in the supplementary materials) in line with this point: LASSO regression and performance when selecting terms by correlation (pandemic and seasonal periods). We now also mention this in the discussion, as suggested.

It reads: (Lines 641–654) “In fact, including all available features can introduce variance that overwhelms the true signal. Instead, our approach prioritizes coherence, identifying subsets of search terms that behave consistently during outbreaks *(clusters)* and remain predictive beyond crisis contexts (...) That these pandemic-identified terms (particularly those in C1) continue to outperform others across seasonal flu periods suggests they capture underlying behavioral regularities that are robust across time. This finding stands in contrast to purely statistical feature selection methods, such as correlation filtering or LASSO regression, which may perform well within narrow windows but often yield unstable and inconsistent results across datasets”.

3. Redundancy among the searched terms is seen to be the source of bias-variance. For future nowcasting implementation, how is this issue to be addressed technically to increase predictability and accuracy? Include this in the discussion.

This is a very relevant point and in line with a previous comment from reviewer R1. We have added the following sentence to the discussion (in italics): "*This also reinforces that feature selection alone does not guarantee temporal robustness; as search behaviour evolves, maintaining temporal robustness will require approaches that monitor changes in search patterns (e.g., feature drift) and rely on domain expertise for feature interpretation and validation.*" (Lines 658-660)

We also clarify that redundancy can be a source of bias-variance and the results section also reads (new text in italics): "when including too many *or redundant* predictors, models learn patterns that do not generalize beyond the training data" (Lines 466-467)

Again, we thank both the Editors and the Reviewers for their comments and hope that our answers address all the points raised during the review process.
