# Supplementary material for: Learning from pandemics: Feature selection and clustering for robust digital disease nowcasting
Source: PLoS One. 2026 Oct 1;21(10):e0358063. doi: 10.1371/journal.pone.0358063 (PMC13630223; doi:10.1371/journal.pone.0358063)
Supplement: Supplementary file 34 [file pone.0358063.s030.pdf]

We would like to thank the reviewer for the careful reading and recommendation to submit. Below we address the indicated weaknesses and suggestions one by one. All new text is marked in blue in the "'Revised Manuscript with Track Changes'".

1. Cluster validation lacks empirical grounding. Claims like "C1 = symptom-driven" rely on correlations; integrate user surveys (e.g., "Did you search 'fever' due to symptoms?") to confirm behavioral intent.

The issue of empirical grounding for the clusters and the interpretation of behavioral intent is an important one and we thank the reviewer for pointing it out. Indeed, direct validation (e.g., through surveys) was not done, and we think that it carries its own limitations, such as recall bias and lack of representativeness. Instead, our validation relied on independent behavioral proxies, specifically, correlation with case counts and media coverage, which consistently distinguish clusters tracking real disease burden (C1) from those dominated by media-driven attention (C2). We reasoned that this provides a pragmatic, population-level behavioral grounding aligned with the study's epidemiological focus.

We do agree that the previous framing risked possible over-interpretation and/or inferring behavioral intent at the individual level. Therefore, we took four main steps: (1) Clarify that cluster labels (e.g., "C1 = symptom-related") are used solely as population-level summaries and are not intended to infer individual motives; (2) Make explicit that our approach is data-driven and descriptive rather than behavioral in the survey sense; (3) cite previous work showing that different motivations lead to different information-seeking behaviors and to different search patterns (for example, showing that symptom-related health queries are often prompted by personal or family illness, whereas searches about institutions and public measures are more associated with information exposure, risk perception, and collective concern; these patterns have been documented via query logs, surveys, and mixed-methods studies); and (4) removed possible causal phrasing and made the descriptive scope more explicit.

Specifically, in the Introduction we added:

"*Beyond volume, evidence shows that both real risk and risk perception shape online health information seeking [45], and that searching behaviors differ for people who feel ill versus healthy [46, 47]. Queries about institutions or public measures, by contrast, often rise with information exposure and broader public concern. Online searching is also positively associated with intentions to take preventive measures [48, 49].*"

The Results section now reads:

"*Cluster labels were assigned after clustering from the most frequent terms and their temporal behavior and are used only to aid interpretation at the population level.*" (...) "*Still, in what*

follows, cluster labels are used to summarize group behavior, and should not be read as claims about individual motivations." (...) "*Different online search patterns can be explained by different offline drivers*." (...) "*However, these interpretations are ad hoc; an* a priori *assessment would not necessarily anticipate the observed cluster structure, and these clusters cannot be used to infer individual intent.*"

And the Methods Section includes:

"*Cluster labels were assigned after clustering based on the most frequent terms in each cluster and their temporal behavior, and are used only to aid interpretation at the population level. These labels do not make claims about individual motives. They were not used as targets or inputs during model training and do not affect model estimation or evaluation; they serve only to describe groups and to define the C1, C2, and C3 feature sets*."

We hope that these changes address the reviewer's concerns.

2. COVID-19 instability is underexplored. Performance dips during policy/variant shifts (e.g., Omicron); stratify analysis by pandemic phases to test if terms like "loss of smell" lose relevance post-2022.

This is a very important observation, and we appreciate the suggestion. We have now expanded the COVID-19 analysis to examine model performance and feature relevance across dominant variant phases through two complementary analyses (1) Phase-stratified performance: we report RMSE and R^2 within each major variant period and show a clear dip in performance during the Omicron BA.1 transition (winter 2021–2022), consistent with shifts in testing behavior and symptom perception. (2) Term-level feature relevance over time: using weekly Random Forest importances and one-tailed Mann–Whitney U tests with Benjamini–Hochberg correction, we find significant post-2022 declines for symptom-specific queries (e.g., *loss of smell*, *loss taste*), whereas general COVID-related terms (e.g., *covid cases*, *testing*) remain comparatively stable.

Together, these results support the reviewer's prediction that model stability was disrupted during the Omicron phase and that symptom-related behaviors became less predictive in later stages of the pandemic.

This is detailed in the new Supporting information section "Temporal instability and variant-specific performance during the COVID-19 pandemic", which includes two figures(S13–S14) and one Table (S14).

We have also referenced these additions in three sections of the revised manuscript.

The Results section now reads:

"*Finally, to further examine the temporal stability of model performance, we stratified the analysis by dominant COVID-19 variant phases (see S13–S14 Figs and S14 Table in the Supporting information). An increase in RMSE was observed during the Omicron BA.1 transition (winter 2021–2022), indicating a temporary decline in predictive performance coinciding with shifts in testing behavior and symptom perception. In parallel, a longitudinal evaluation of feature importance from the Random Forest models showed that symptom-specific terms such as* loss of smell *and* loss taste *significantly declined in predictive relevance after early 2022, whereas more general COVID-related queries (e.g.,* covid cases, testing*) remained relatively stable. These findings suggest that changes in strain dominance coincide with changes in search patterns, emphasizing the importance of continuously incorporating behavioral dynamics into nowcasting frameworks.*"

In the Discussion, we have added:

"*Consistent with this view, the variant-stratified analysis highlights that model performance can shift with behavior and symptom frequency (e.g., declining value of loss of smell after Omicron). This also reinforces that feature selection alone does not guarantee temporal robustness; models should incorporate continuous mechanisms for behavior-aware adaptation (e.g., monitoring feature drift and refreshing feature sets) to remain reliable in real-life settings.*"

The Methods section includes a new subsection (**"**Variant-stratified performance and feature-importance tests"), which reads:

"*For the variant-stratified analyses, the COVID-19 timeline was divided into seven non-overlapping phases using inclusive start and end dates to tag each week: Pre-Alpha (March 15, 2020 to December 13, 2020), Alpha (December 14, 2020 to June 20, 2021), Delta (June 21, 2021 to December 12, 2021), Omicron BA.1 (December 13, 2021 to March 20, 2022), Omicron BA.2 (March 21, 2022 to June 19, 2022), Omicron BA.5 (June 20, 2022 to October 16, 2022), and Omicron XBB+ (October 17, 2022 to May 7, 2023) [59]. Phase-specific performance metrics (RMSE and R^2) were computed using only the weekly predictions within each phase, and a four-week rolling RMSE was also calculated to capture short-term fluctuations. To examine whether individual search terms changed in predictive relevance, weekly Random Forest feature importances from the 'ALL' model were used and plotted in a term-by-week heatmap with vertical lines marking the variant boundaries. For each term, we then tested whether importance decreased after January 2022 using a one-tailed Mann–Whitney U test (pre-2022 vs post-2022), applying Benjamini–Hochberg FDR correction [60] at 0.05. Terms with a significant decline are marked with an asterisk in the heatmap (S14 Fig). This procedure quantifies when*

specific search behaviors (e.g., 'loss of smell') lost predictive value as public perception and testing practices evolved."

We believe that the new analysis strengthened a central argument of the paper, that topical knowledge and behavioral dynamics must be accounted for in reliable nowcasting.

3. Reproducibility risks from unofficial data tools. Benchmark against official Google Trends exports for high-impact periods (e.g., 2018 flu) to quantify sampling biases.

We thank the reviewer for another relevant point. Indeed, there are advantages and disadvantages of each methodology, which were not appropriately discussed. Our analysis was done at a time of unreliable official access - across different online platforms-, and we opted to avoid systems of privileged gatekeeping, such as proprietary APIs, that could change or be discontinued. Still, this choice should have been benchmarked.

Therefore, we have now compared the *pytrends*-based with the official *Google Trends* CSV exports downloaded directly from the web interface. We did this for both H1N1 (2009–2010) and COVID-19 (2020–2023), including terms from all three clusters.

We note that these two extractions differ in timing and native resolution: the original extraction was done months to years earlier and used an "extended" pipeline that combines monthly and daily pulls to construct a consistent weekly series (please see Methods); the new exports were download this month and provide weekly values directly. However, and despite these differences, the two sources closely align (S7–S8 Tables and S8–S9 Figs in the Supporting information), with correlations consistently high (median Pearson > 0.98) and with minimal mean absolute differences. This indicates that *pytrends* reproduces the official Google Trends signal, with negligible impact from sampling variability, retrieval date, or construction method.

Concretely, following the reviewer's comment, the revised version of the manuscript describes these analyses in detail in the Supporting information which also includes two tables (S7 and S8 Tables - Reproducibility of Google Trends extraction for Flu and COVID-19 respectively, -*pytrends* vs official exports), and two figures (S8 and S9 Fig. Reproducibility: pytrends vs official exports for flu and COVID-19,, respectively).

These steps and results are referenced in the Main Manuscript, while arguing that, for a public-health tool, relying on a pipeline that performs robustly even under standard UI/API sampling noise is more sustainable than one that depends on special or unstable access.

Concretely, we added/clarified the Results section to read:

"*Second, we further tested whether the results were an artifact of the data retrieval tool (*pytrends*), by comparing these time series with the official Google Trends CSV exports (two queries per cluster). For all tested terms, correlations were very high (all Pearson and Spearman coefficients > 0.94; S7 Table for flu and S8 Table for COVID-19), and temporal profiles were similar (S8 and S9 Figs). These controls confirm the reliability of the automated pipeline in producing consistent estimates of search interest over time, supporting its use for epidemiological applications that require periodic data updates*."

The Methods now also include:

"*To verify consistency, we benchmarked pytrends outputs against manual downloads from the Google Trends website for the same queries, locations, and dates (see S8–S9 Figs in the Supporting information).*"

And the Discussion has the following reproducibility note:

"*Finally, for public health use, analysis must remain consistent and reproducible. A method that relies on private platform data, as is the case of Google Trends and its API, risks interruptions or instability from changing access paths, shifting policies, quota limits, pricing, and even basic availability. Although interface sampling can introduce minor run-to-run variation, it can minimize some of these issues. In this work, repeated exports show that comparative results remain stable (see S8–S9 Figs in the Supporting information), and preference should be given to pipelines resilient to platform variability. Still, they do not solve the fundamental problem of relying on non-public data*."

4. Linear Regression superiority unexplained. Diagnose Random Forest failures during outbreaks (e.g., 2018 flu overprediction) via residual analysis to justify simplicity.

Another important point, one that we discussed internally and debated whether to include in the first submission. We have now attempted to clarify how and why the linear regression model outperforms the random forest. Specifically, what we observe (and what is expected from previous work, new references [61-63]), is an underprediction by the random forest when the test dataset includes values that are outside the range of values in the training dataset. In other words, the expectation is that the random forest fails in periods of very high intensity. We performed these comparisons and the results are shown in the supplementary figures S4 (flu) and S7 (COVID-19). S4 shows that the errors of the random forest model are especially large when incidence is high, while the linear regression residuals do not depend on incidence so

much. In the case of COVID-19, both models have difficulties predicting high incidence values (figure S7). We offer possible explanations in the manuscript.

The Results section now reads, regarding the flu predictions: *"Overall, Linear Regression outperformed Random Forest in five out of six test cases, particularly when the validation period included severe flu seasons (i.e., seasons with high peaks of reported cases), with 2018 being the most glaring example. This can be explained by the large differences in amplitude between training and testing data. Random forests perform regression tasks by building trees where each branch partitions the dependent variable arbitrarily [61]. Since each tree will have its own cutoffs, this makes the models very good at tackling non-linear problems. However, they cannot learn any partition beyond the range present in the training data. If the features point to a very high (low) value, then the trained model will predict the highest (lowest) value present in the training data. If so, the expectation is that Random Forest will perform the poorest when the highest or lowest values were not present in the training data. Indeed, this is what S3 Fig shows, and has been observed by others (e.g. [62, 63]): the only exception to the Random Forest under-performance was P6, for which the test period did not include the unusually intense 2018 flu outbreak. Model performance and full comparisons between observed and predicted cases for each period are provided in S4 Table and S2–S4 Figs in the Supporting information."*

And regarding the COVID-19 predictions: *"In this case, the Linear Regression models also outperformed the Random Forest, but likely for different reasons than in the case of the flu. Here, both models are equally poor at forecasting the largest peak (figures S5, S6 and S7), but the Random Forest predictions are generally worse, while the Linear Regression fails less consistently. One possible explanation is the ARGO model using past predictions as a feature, while the Random Forest does not include such auto-correlation inputs."*

5. Ethical gaps. Add brief guidance on privacy (e.g., aggregate geographic data) and digital-access equity to ensure responsible deployment.

This is a very important note, and we apologize for the oversight.

The following text has been added to the top of the Methods section:

"*Health data is considered sensitive under the EU GDPR. In this study only previously anonymized and aggregated data was used*."

Under section "Search Trend data" we have added:

"*Data extracted from GT returns the normalized, scaled and aggregated total number of searches for a particular query, in a specified geographical region and time range. All data is anonymous and it is virtually impossible to identify individual searchers*."

In the Discussion section we have added:

"*Indeed, if deployed responsibly, this method also offers promise to help reduce health inequities by providing rapid insights. Curated search terms, if adjusted for local behaviors and disparities in digital access, can improve disease surveillance without reinforcing existing digital divides*."

Again, we thank the Reviewer for the thoughtful comments, believe to have addressed all concerns, and find that our works was greatly strengthened by the revision.

We have also carefully revised to manuscript to correct grammar inconsistencies (e.g. verb tenses in the Methods), and minor discrepancies (e.g. consistent use of italics or capital letters).
