# Supplementary material for: Learning from pandemics: Feature selection and clustering for robust digital disease nowcasting
Source: PLoS One. 2026 Oct 1;21(10):e0358063. doi: 10.1371/journal.pone.0358063 (PMC13630223; doi:10.1371/journal.pone.0358063)
Supplement: Supplementary file 36 [file pone.0358063.s033.pdf]

We thank the Academic Editor and the reviewers for the time and effort invested in the review process and for their relevant comments and suggestions. We have carefully considered all comments and have revised the manuscript accordingly. Below, in blue, we provide a detailed, point-by-point response to each comment. Changes made to the manuscript are indicated in italics throughout this document and in blue in the manuscript.

Reviewer 3

The paper titled "Learning from Pandemics: Feature Selection for Robust Digital Disease Nowcasting" requires essential improvements. Some sections of the present version of the revision remained unclear and then reviewer is worrying about scientific and originality of the paper contents.

We thank R3 for the detailed comments and while we agree with several suggestions we respectfully try to justify why some others might be outside of the scope of the current work.

1-In abstract section, authors need to mention which Machine Learning Models (MLMs) were used in this study with their main purposes. The main findings along with its conditions of datasets can be merged to the abstract section.

To comply both with the reviewer's request and with the journal's word formatting requirements, the following sentence has been added to the abstract (in italics):

"We propose a hybrid approach to improve prediction that combines model evaluation with behavioral insight. *We apply simple supervised and unsupervised methods to different* flu and COVID-19 *datasets*, using the 2009 H1N1 and 2020 COVID-19 pandemics as training periods. In both cases, selecting a curated subset of search terms improved model performance, reduced overfitting, and increased stability, consistently outperforming models that used search terms indiscriminately."

2-Introduction section requires diverse related research works, describing application of MLMs in the case of feature selection and capability of robust MLMs. Reviewer is now considering table containing literature review in this area whereas this table need a lot of major modifications.

For Feature Selection:
-Unsupervised feature selection via graph-based proximity and structured autoencoder-like NMF
-Joint sample-feature subspace learning via bidirectional reconstruction for feature selection
For robustness:
-Robust oblique projection and weighted NMF for hyperspectral unmixing
-Simultaneous outlier detection and elimination in hyperspectral unmixing via weighted non-negative matrix tri-factorization

We thank the reviewer for these suggestions and carefully considered the recommended references. While some of these articles fall outside the scope of the present study, as the manuscript focuses on the application of machine learning methods to digital epidemiology and disease nowcasting rather than on methodological developments in machine learning algorithms, we see how the paper could benefit from a broader overview of recent work in this area. Accordingly, we have updated it with references covering (i) applications of machine learning in epidemiology, (ii) recent developments in feature selection methodologies, and (iii) recent advances in infectious disease forecasting using digital data. In particular, we have added:

1 - LLM-Assisted Keyword Curation for Early Disease Outbreak Detection Using Search Trends, by Brahmos and Princess (2025), which reinforces the importance of careful feature selection in search-based epidemiology and provides a complementary, LLM-assisted approach to keyword curation.

Added to the Introduction (Lines 19-23): "*As the use of online search data for disease surveillance has matured, attention has shifted from whether these data are useful to how the most informative search terms should be selected. Even with recent advances in artificial intelligence, careful keyword curation remains essential for identifying the search terms that carry the most relevant information for disease prediction* [19]."

2 - "Machine learning in epidemiology: An introduction, comparison with traditional methods, and a case study of predicting extreme longevity", a recent review by Goldstein et al., which discusses the application of machine learning in epidemiology and reinforces the importance of selecting methods according to the epidemiological question being addressed

Added to the Discussion (Lines 663–665): "*This is consistent with recent perspectives arguing that, in epidemiology, machine learning methods should be selected according to the question being addressed rather than solely on predictive performance* [74]."

3 – "Mathematical methods in feature selection: A review", by Kamalov et al. (2025), which classifies feature selection methods according to their mathematical foundations and discusses their respective strengths, limitations, and areas of application, providing additional context for the approaches evaluated in the present study.

4 – "Recent advances in feature selection and its applications", by Li et al., which reviews recent developments in feature selection and highlights the importance of selecting relevant and non-redundant features to improve both predictive performance and model interpretability. This provides additional theoretical context for the behaviourally informed feature selection strategy proposed in the present study.

Citations 3 and 4 were added to the Introduction (Lines 88—90): "*Recent reviews have emphasized that effective feature selection should identify informative predictors while reducing redundancy, thereby improving predictive performance, robustness, and model interpretability* [52, 53]."

5 - Advancing real-time infectious disease forecasting using large language models, by Du et al. (2025), which provides a recent overview of advances in infectious disease forecasting using artificial intelligence and highlights the growing integration of digital and heterogeneous data sources to complement traditional surveillance systems.

Added to the Introduction (Lines 7--10): "*As surveillance mechanisms relying solely on highly trained personnel are often expensive, limited, and slow, it has been extensively argued that traditional methods should be complemented with 'Digital Era' tools, such as online activity, mobility patterns, or digital contact-tracing* [1, 2, 3, 4]."

6 - Forecasting dengue cases through time-series modeling with Google Trends and deep neural networks, by Cheong et al. (2025), which demonstrates that parsimonious Google Trends feature sets can outperform larger collections of search terms in infectious disease forecasting. While their feature selection is based on statistical associations,

our work provides a complementary behavioural perspective that helps explain why the indiscriminate inclusion of search terms may reduce predictive performance.

Added to the Results (Lines 507--509): "*This is consistent with recent work on dengue forecasting using Google Trends, where parsimonious search-term sets selected through statistical associations outperformed larger collections of search terms* [72]*.*"

3-Research gap and novelty should be deeply discussed in the introduction section.

We agree that clearly defining the research gap and novelty is important. To address this comment, we revised the final paragraph of the Introduction to more clearly distinguish our contribution from previous work. Specifically, we now explicitly identify the research gap and clarify the novelty of the proposed approach. The Introduction now reads:

"*Recent reviews have emphasized that effective feature selection should identify informative predictors while reducing redundancy, thereby improving predictive performance, robustness, and model interpretability. However, relatively little attention has been paid to whether pandemic periods themselves can be exploited to improve feature selection. To address this gap, we propose a simple and explainable feature selection framework that leverages the high-intensity periods of pandemics as natural experiments to identify search terms with greater temporal robustness for disease nowcasting*."(Lines 88–95).

4-Research organization, known as the last paragraph of introduction section should be deeply provided.

To address this comment, we expanded the final paragraph of the Introduction to include a brief overview of the organization of the manuscript. After introducing the research gap and the proposed method, we now briefly describe how the study is structured, from the presentation of the data sources and modelling approach to the evaluation of the proposed method and the discussion of its implications.

The Introduction now concludes with:

"To evaluate this approach, we first describe the data sources and modelling approach, then assess its performance across influenza and COVID-19 case studies, and finally discuss its implications for the design of robust and interpretable disease nowcasting systems." (Lines 98-101)

5- Delete subsections in the introduction section. And more, all the subsections should have had only single name as "Introduction" in which includes (i) importance of subject and background theorem, (ii) literature review with emphasize on major drawbacks of empirical/traditional methodologies equations, (iii) research gap and novelty, and (iv) research organization in which overview of the present research should be mentioned in a paragraph.

We ask the reviewer to confirm whether the current version of the manuscript complies with that request or offer more detail on how it should be improved.

6-Details of case study and some important discussions on the datasets should be detailed.

We ask the reviewer to confirm whether the current version of the manuscript complies with that request or offer more detail on how it should be improved.

7-Authors are emphatically recommended to use MLMs such as KNN, Model Tree (MT), AdaBoost, XGBoost, CatBoost, LightBoost, and RF for the training and testing stages.

Algorithms such as KNN, Model Trees, AdaBoost, XGBoost, CatBoost, and LightGBM are indeed powerful machine learning methods and can provide great predictive performance in many applications. However, since the primary objective of the present study was to evaluate whether behaviourally informed feature selection improves the robustness and generalizability of disease nowcasting models, we decided against testing a broad range of models and selected two complementary and widely used approaches: Linear Regression, as a simple and fully interpretable baseline, and Random Forest, as a nonlinear ensemble method capable of capturing more complex relationships.

The fact that the proposed feature selection strategy consistently improved performance across both model classes suggests that the observed gains arise from the quality of the selected features and not from a particular algorithm. While evaluating additional algorithms such as gradient boosting methods would certainly be an interesting direction for future work, we respectfully argue that it falls outside the scope of the present study and would not substantially alter its main conclusions.

9-Descriptions on every MLM should be presented in a separate subsection along with the setting parameters of each predictive models and the corresponding justifications. In this section, authors should use AdaBoost, CatBoost, LighGBoost, XGBoost, KNN, SVM, RF, and other regression models based on machine learning techniques (ElasNet, Lasso, and Ridge).

To improve this section, authors need to use the material of the following papers:

- Vulnerability of the rip current phenomenon in marine environments using machine learning models

- Assessment of flood susceptibility in Minab County, Iran, through the integration of topographic, climatic, and land-surface indices using ensemble machine learning models

- Ultrasonic flowmeter fault early warning method based on Bayesian optimization XGBoost

- Evaluation of river water quality index using remote sensing and artificial intelligence models

- A Stacked Machine Learning Algorithm for Multi-Step Ahead Prediction of Soil Moisture

Please see our response to Comment 7 regarding the rationale for the selection of the predictive models evaluated in this study, as the same considerations apply here.

10- Instead using R^2, authors are emphatically recommended to use Correlation of Coefficient (CC), RMSE, Mean Absolute Error (MAE), Mean Bias Error (MBE), Discrepancy Ratio (DR), and Scatter Index (SI) to this section. These statistical measures should be computed for the training and testing stages of the present predictive models. All the formulations and acceptable ranges of these statistical measures should be merged to this section. For this purpose, read the following papers and add them to the present revisions.

-A hybrid hourly natural gas demand forecasting method based on the integration of wavelet transform and enhanced Deep-RNN model

-A deep reinforcement learning-based method for predictive management of demand response in natural gas pipeline networks

-A Combinatorial Machine Learning Models -Based Method for Predicting the Viscosity-Temperature Relationship of Crude Oil

-Receiving More Accurate Predictions for Longitudinal Dispersion Coefficient in Water Pipelines: Training Group Method of Data Handling Using Extreme Learning Machine Conceptions

-A Method for Predicting the Gel Point of Mixed Crude Oil Based on Deep Learning

- A Large Language Model Based Knowledge Mining Method for Improving the Reliability of Fire Water Systems

- Optimization Method of Natural Gas Pipeline Network Emergency Dispatch Based on Particle Swarm Optimization

-Data-driven reliability assessment method of Integrated Energy Systems based on probabilistic deep learning and Gaussian mixture Model-Hidden Markov Model

-Interpretable Combinatorial Machine Learning-Based Shale Fracability Evaluation Methods

-Dynamic Prediction of Natural Gas Calorific Value Based on Deep Learning

-Water quality monitoring for coastal hypoxia: Integration of satellite imagery and machine learning models

-Dynamic damage functions for scour protection at mono-pile foundations: Application of ensemble machine learning models

-A new correlation for calculating carbon dioxide minimum miscibility pressure based on multi-gene genetic programming

We ask the reviewer to confirm whether the current version of the manuscript complies with that request. In addition to R2, and partially also in response to reviewer R1, we added bias-variance decomposition (Methods, Bias-variance trade-off subsection), and RMSE-based analyses, including phase-specific and rolling RMSE evaluations (Methods, Variant-stratified evaluation subsection; Results, Overfitting reduction, generalization, and predictive reliability in feature selection subsection; S14 Table).

11-Use Scatter plots, Taylor's diagrams, heat maps, and violin plots to illustrate the results of the training and testing stages.

We agree that visual assessment is very useful when evaluating models. In the current manuscript and Supporting Information, we already provide several complementary visualizations of model performance, including line plots comparing observed and predicted trajectories for both Linear Regression and Random Forest models in the flu and COVID-19 analyses (S2–S3 and S5–S6 Figs), scatter plots relating residuals to observed incidence, line plots showing the temporal evolution of residuals, histograms illustrating residual and incidence distributions, and binned summary plots of mean residuals across incidence levels (S4 and S7 Figs). In the main manuscript, we also compare model performance across feature sets (Fig. 2). In addition, the manuscript includes heatmaps illustrating the longitudinal evolution of feature importance across SARS-CoV-2 variant periods, as requested by another reviewer.

It is our assessment that the current set of figures provides sufficient visual information to evaluate model performance and prediction errors across the training and testing settings. While Taylor diagrams and violin plots could provide an alternative way of visualizing some of these results, we are not sure that they would provide additional insight. If the reviewer feels that a particular aspect of model performance would benefit from additional visualization, we would appreciate more specific clarification so that we can better understand the concern and address it as effectively as possible.

12-The present results should be compared with those related papers (use more than 15 papers at least with quantitative and qualitative comparisons).

Please see answer to comment 2.

Reviewer #6:

Based on the manuscript contents and contexts, all the above questions seem to fulfill the PLOS requirements.

We thank the reviewer for the positive and constructive comments and overall recommendation to publish.

However, there are a few comments to the authors for consideration, which include:

1. The manuscript title is "Learning from Pandemics: Feature Selection for Robust Digital Disease Nowcasting." Discussions are focused on how to improve prediction that combines model evaluation with behavioral insight using curated features. Since hierarchical clustering is applied in data processing and the clustering results (C1, C2, C3) become the input for the rest of the analysis for 2009 H1N1, while for Covid-19 the predefined period (P1, P2, ..., P6) is applied, I would suggest revising the title to use "Feature Clustering" instead. For the "Feature Selection" to be used, discussions shall highlight how the number of searched terms is reduced in quantity (leaving only the significant terms) that would help improve the prediction. Consider this if it is viable.

We thank the reviewer for the comment. It is not only viable, but it also offers a better description of the work. We have updated the title as suggested.

2. Searched terms in the online may be an intercept between different aspects of symptoms, treatment, personal care, institutions, and pandemic-specific terms. As this led to redundancy, how does this study see this as a threat to the accuracy of nowcasting? Add your comments in the discussion if it has not been addressed.

We agree both with the note that search terms can have different natures and underlying motivations, and that redundancy is often a problem. We cluster similar terms and include two types of analysis (in the supplementary materials) in line with this point: LASSO regression and performance when selecting terms by correlation (pandemic and seasonal periods). We now also mention this in the discussion, as suggested.

It reads: (Lines 641–654) “In fact, including all available features can introduce variance that overwhelms the true signal. Instead, our approach prioritizes coherence, identifying subsets of search terms that behave consistently during outbreaks *(clusters)* and remain predictive beyond crisis contexts (...) That these pandemic-identified terms (particularly those in C1) continue to outperform others across seasonal flu periods suggests they capture underlying behavioral regularities that are robust across time. This finding stands in contrast to purely statistical feature selection methods, such as correlation filtering or LASSO regression, which may perform well within narrow windows but often yield unstable and inconsistent results across datasets”.

3. Redundancy among the searched terms is seen to be the source of bias-variance. For future nowcasting implementation, how is this issue to be addressed technically to increase predictability and accuracy? Include this in the discussion.

This is a very relevant point and in line with a previous comment from reviewer R1. We have added the following sentence to the discussion (in italics): "*This also reinforces that feature selection alone does not guarantee temporal robustness; as search behaviour evolves, maintaining temporal robustness will require approaches that monitor changes in search patterns (e.g., feature drift) and rely on domain expertise for feature interpretation and validation.*" (Lines 658-660)

We also clarify that redundancy can be a source of bias-variance and the results section also reads (new text in italics): "when including too many *or redundant* predictors, models learn patterns that do not generalize beyond the training data" (Lines 466-467)

Again, we thank both the Editors and the Reviewers for their comments and hope that our answers address all the points raised during the review process.
