# Supplementary material for: Learning from pandemics: Feature selection and clustering for robust digital disease nowcasting
Source: PLoS One. 2026 Oct 1;21(10):e0358063. doi: 10.1371/journal.pone.0358063 (PMC13630223; doi:10.1371/journal.pone.0358063)
Supplement: Supplementary file 37 [file pone.0358063.s031.docx]

The paper titled “Learning from Pandemics: Feature Selection for Robust Digital Disease Nowcasting” requires essential improvements. Some sections of the present version of the revision remained unclear and then reviewer is worrying about scientific and originality of the paper contents.

1-In abstract section, authors need to mention which Machine Learning Models (MLMs) were used in this study with their main purposes. The main findings along with its conditions of datasets can be merged to the abstract section.

2-Introduction section requires diverse related research works, describing application of MLMs in the case of feature selection and capability of robust MLMs. Reviewer is now considering table containing literature review in this area whereas this table need a lot of major modifications.

For Feature Selection:

-Unsupervised feature selection via graph-based proximity and structured autoencoder-like NMF

-Joint sample-feature subspace learning via bidirectional reconstruction for feature selection

For robustness:

-Robust oblique projection and weighted NMF for hyperspectral unmixing

-Simultaneous outlier detection and elimination in hyperspectral unmixing via weighted non-negative matrix tri-factorization

3-Research gap and novelty should be deeply discussed in the introduction section.

4-Research organization, known as the last paragraph of introduction section should be deeply provided.

5- Delete subsections in the introduction section. And more, all the subsections should have had only single name as “Introduction” in which includes (i) importance of subject and background theorem, (ii) literature review with emphasize on major drawbacks of empirical/traditional methodologies equations, (iii) research gap and novelty, and (iv) research organization in which overview of the present research should be mentioned in a paragraph.

6-Details of case study and some important discussions on the datasets should be detailed.

7-Authors are emphatically recommended to use MLMs such as KNN, Model Tree (MT), AdaBoost, XGBoost, CatBoost, LightBoost, and RF for the training and testing stages.

9-Descriptions on every MLM should be presented in a separate subsection along with the setting parameters of each predictive models and the corresponding justifications. In this section, authors should use AdaBoost, CatBoost, LighGBoost, XGBoost, KNN, SVM, RF, and other regression models based on machine learning techniques (ElasNet, Lasso, and Ridge).

To improve this section, authors need to use the material of the following papers:

*Vulnerability of the rip current phenomenon in marine environments using machine learning models

*[Assessment of flood susceptibility in Minab County, Iran, through the integration of topographic, climatic, and land-surface indices using ensemble machine learning models](https://scholar.google.com/citations?view_op=view_citation&hl=en&user=S5mF7z4AAAAJ&sortby=pubdate&citation_for_view=S5mF7z4AAAAJ:4vMrXwiscB8C)

*Ultrasonic flowmeter fault early warning method based on Bayesian optimization XGBoost

*[Evaluation of river water quality index using remote sensing and artificial intelligence models](https://scholar.google.com/citations?view_op=view_citation&hl=en&user=S5mF7z4AAAAJ&citation_for_view=S5mF7z4AAAAJ:edDO8Oi4QzsC)

*[A Stacked Machine Learning Algorithm for Multi-Step Ahead Prediction of Soil Moisture](https://scholar.google.com/citations?view_op=view_citation&hl=en&user=S5mF7z4AAAAJ&cstart=20&pagesize=80&citation_for_view=S5mF7z4AAAAJ:Dip1O2bNi0gC)

10- Instead using R^2^, authors are emphatically recommended to use Correlation of Coefficient (CC), RMSE, Mean Absolute Error (MAE), Mean Bias Error (MBE), Discrepancy Ratio (DR), and Scatter Index (SI) to this section. These statistical measures should be computed for the training and testing stages of the present predictive models. All the formulations and acceptable ranges of these statistical measures should be merged to this section. For this purpose, read the following papers and add them to the present revisions.

-A hybrid hourly natural gas demand forecasting method based on the integration of wavelet transform and enhanced Deep-RNN model

-A deep reinforcement learning-based method for predictive management of demand response in natural gas pipeline networks

-A Combinatorial Machine Learning Models -Based Method for Predicting the Viscosity-Temperature Relationship of Crude Oil

-Receiving More Accurate Predictions for Longitudinal Dispersion Coefficient in Water Pipelines: Training Group Method of Data Handling Using Extreme Learning Machine Conceptions

-A Method for Predicting the Gel Point of Mixed Crude Oil Based on Deep Learning

- A Large Language Model Based Knowledge Mining Method for Improving the Reliability of Fire Water Systems

- Optimization Method of Natural Gas Pipeline Network Emergency Dispatch Based on Particle Swarm Optimization

-Data-driven reliability assessment method of Integrated Energy Systems based on probabilistic deep learning and Gaussian mixture Model-Hidden Markov Model

-Interpretable Combinatorial Machine Learning-Based Shale Fracability Evaluation Methods

-Dynamic Prediction of Natural Gas Calorific Value Based on Deep Learning

-[Water quality monitoring for coastal hypoxia: Integration of satellite imagery and machine learning models](https://scholar.google.com/citations?view_op=view_citation&hl=en&user=S5mF7z4AAAAJ&sortby=pubdate&citation_for_view=S5mF7z4AAAAJ:WJVC3Jt7v1AC)

-[Dynamic damage functions for scour protection at mono-pile foundations: Application of ensemble machine learning models](https://scholar.google.com/citations?view_op=view_citation&hl=en&user=S5mF7z4AAAAJ&sortby=pubdate&citation_for_view=S5mF7z4AAAAJ:NXb4pA-qfm4C)

-[A new correlation for calculating carbon dioxide minimum miscibility pressure based on multi-gene genetic programming](https://scholar.google.com/citations?view_op=view_citation&hl=en&user=S5mF7z4AAAAJ&cstart=20&pagesize=80&citation_for_view=S5mF7z4AAAAJ:K3LRdlH-MEoC)

11-Use Scatter plots, Taylor’s diagrams, heat maps, and violin plots to illustrate the results of the training and testing stages.
