# Supplementary material for: Learning from pandemics: Feature selection and clustering for robust digital disease nowcasting
Source: PLoS One. 2026 Oct 1;21(10):e0358063. doi: 10.1371/journal.pone.0358063 (PMC13630223; doi:10.1371/journal.pone.0358063)
Supplement: Peer review history

# Peer review: Decision Letter 0

**DOI:** 10.1371/journal.pone.0358063.r001

**Authors:** Miquel Vall-llosera Camps

25 Sep 2025

Dear Dr. Gonçalves-Sá,

Thank you for submitting your manuscript to PLOS ONE. After careful consideration, we feel that it has merit but does not fully meet PLOS ONE’s publication criteria as it currently stands. Therefore, we invite you to submit a revised version of the manuscript that addresses the points raised during the review process.

I would like to sincerely apologise for the delay you have incurred with your submission. It has been exceptionally difficult to secure reviewers to evaluate your study. We have now received two completed reviews; the comments are available below. The reviewers have raised significant scientific concerns about the study that need to be addressed in a revision.

Please revise the manuscript to address all the reviewer's comments in a point-by-point response in order to ensure it is meeting the journal's publication criteria. Please note that the revised manuscript will need to undergo further review, we thus cannot at this point anticipate the outcome of the evaluation process.

Please submit your revised manuscript by Nov 08 2025 11:59PM. If you will need more time than this to complete your revisions, please reply to this message or contact the journal office at plosone@plos.org. When you're ready to submit your revision, log on to https://www.editorialmanager.com/pone/ and select the 'Submissions Needing Revision' folder to locate your manuscript file.

- A rebuttal letter that responds to each point raised by the academic editor and reviewer(s). You should upload this letter as a separate file labeled 'Response to Reviewers'.
- A marked-up copy of your manuscript that highlights changes made to the original version. You should upload this as a separate file labeled 'Revised Manuscript with Track Changes'.
- An unmarked version of your revised paper without tracked changes. You should upload this as a separate file labeled 'Manuscript'.

If you would like to make changes to your financial disclosure, please include your updated statement in your cover letter. Guidelines for resubmitting your figure files are available below the reviewer comments at the end of this letter.

If applicable, we recommend that you deposit your laboratory protocols in protocols.io to enhance the reproducibility of your results. Protocols.io assigns your protocol its own identifier (DOI) so that it can be cited independently in the future. For instructions see: https://journals.plos.org/plosone/s/submission-guidelines#loc-laboratory-protocols. Additionally, PLOS ONE offers an option for publishing peer-reviewed Lab Protocol articles, which describe protocols hosted on protocols.io. Read more information on sharing protocols at https://plos.org/protocols?utm_medium=editorial-email&utm_source=authorletters&utm_campaign=protocols.

We look forward to receiving your revised manuscript.

Kind regards,

Miquel Vall-llosera Camps

Senior Staff Editor

PLOS ONE

Journal Requirements:

When submitting your revision, we need you to address these additional requirements.

1. Please ensure that your manuscript meets PLOS ONE's style requirements, including those for file naming. The PLOS ONE style templates can be found at

https://journals.plos.org/plosone/s/file?id=wjVg/PLOSOne_formatting_sample_main_body.pdf and https://journals.plos.org/plosone/s/file?id=ba62/PLOSOne_formatting_sample_title_authors_affiliations.pdf

2. Thank you for stating in your Funding Statement:

[This study was funded by the Portuguese Foundation for Science and Technology (FCT) through the PhD fellowships SFRH/BD/139322/2018 and 2020.10157.BD awarded to CHV and SM, respectively. Additional support was provided by the FCT grant DSAIPA/AI/0087/2018 to JGS, and by the European Union – NextGenerationEU through the project grant 2024.07331.IACDC.].

Please provide an amended statement that declares *all* the funding or sources of support (whether external or internal to your organization) received during this study, as detailed online in our guide for authors at http://journals.plos.org/plosone/s/submit-now.  Please also include the statement “There was no additional external funding received for this study.” in your updated Funding Statement.

Please include your amended Funding Statement within your cover letter. We will change the online submission form on your behalf.

3. Thank you for stating the following in the Acknowledgments Section of your manuscript:

[We thank the members of the Social Physics and Complexity (SPAC) group at LIP for their comments and critical reading of the manuscript. This study was funded by the Portuguese Foundation for Science and Technology (FCT) through the PhD fellowships SFRH/BD/139322/2018 and 2020.10157.BD awarded to CHV and SM, respectively. Additional support was provided by the FCT grant DSAIPA/AI/0087/2018 to JGS, and by the European Union – NextGenerationEU through the project grant 2024.07331.IACDC. The funders had no role in the study design, data collection, analysis, interpretation of results, or manuscript preparation.]

We note that you have provided funding information that is currently declared in your Funding Statement. However, funding information should not appear in the Acknowledgments section or other areas of your manuscript. We will only publish funding information present in the Funding Statement section of the online submission form.

Please remove any funding-related text from the manuscript and let us know how you would like to update your Funding Statement. Currently, your Funding Statement reads as follows:

[This study was funded by the Portuguese Foundation for Science and Technology (FCT) through the PhD fellowships SFRH/BD/139322/2018 and 2020.10157.BD awarded to CHV and SM, respectively. Additional support was provided by the FCT grant DSAIPA/AI/0087/2018 to JGS, and by the European Union – NextGenerationEU through the project grant 2024.07331.IACDC.]

Please include your amended statements within your cover letter; we will change the online submission form on your behalf.

4. When completing the data availability statement of the submission form, you indicated that you will make your data available on acceptance. We strongly recommend all authors decide on a data sharing plan before acceptance, as the process can be lengthy and hold up publication timelines. Please note that, though access restrictions are acceptable now, your entire data will need to be made freely accessible if your manuscript is accepted for publication. This policy applies to all data except where public deposition would breach compliance with the protocol approved by your research ethics board. If you are unable to adhere to our open data policy, please kindly revise your statement to explain your reasoning and we will seek the editor's input on an exemption. Please be assured that, once you have provided your new statement, the assessment of your exemption will not hold up the peer review process.

5. We are unable to open your Figure files [Figure1.eps, Figure2.eps, Figure3.eps]. Please kindly revise as necessary and re-upload.

6. Please upload a copy of Supplementary Figures S1, S2, S3, S4, S5, S6, S7, and S8.

If the reviewer comments include a recommendation to cite specific previously published works, please review and evaluate these publications to determine whether they are relevant and should be cited. There is no requirement to cite these works unless the editor has indicated otherwise.

[Note: HTML markup is below. Please do not edit.]

Reviewers' comments:

Reviewer's Responses to Questions

**Comments to the Author**

1. Is the manuscript technically sound, and do the data support the conclusions?

Reviewer #1: Yes

Reviewer #2: Yes

**********

2. Has the statistical analysis been performed appropriately and rigorously?

Reviewer #1: Yes

Reviewer #2: Yes

**********

3. Have the authors made all data underlying the findings in their manuscript fully available?

The PLOS Data policy

Reviewer #1: Yes

Reviewer #2: Yes

**********

4. Is the manuscript presented in an intelligible fashion and written in standard English?

Reviewer #1: Yes

Reviewer #2: Yes

**********

Reviewer #1: Strengths: The study introduces a valuable hybrid approach using pandemic-driven behavioral insights (via hierarchical clustering) to curate search term subsets (e.g., symptom-focused "C1"). This significantly enhances nowcasting robustness for seasonal flu (reduced overfitting, 5/6 seasons) and COVID-19, outperforming bulk-term models. The framework is interpretable and scalable for public health.

Weaknesses & Suggestions:

Cluster validation lacks empirical grounding. Claims like "C1 = symptom-driven" rely on correlations; integrate user surveys (e.g., "Did you search 'fever' due to symptoms?") to confirm behavioral intent.

COVID-19 instability is underexplored. Performance dips during policy/variant shifts (e.g., Omicron); stratify analysis by pandemic phases to test if terms like "loss of smell" lose relevance post-2022.

Reproducibility risks from unofficial data tools. Benchmark against official Google Trends exports for high-impact periods (e.g., 2018 flu) to quantify sampling biases.

Linear Regression superiority unexplained. Diagnose Random Forest failures during outbreaks (e.g., 2018 flu overprediction) via residual analysis to justify simplicity.

Ethical gaps. Add brief guidance on privacy (e.g., aggregate geographic data) and digital-access equity to ensure responsible deployment.

Recommendation: Accept with minor revisions. Address behavioral validation and COVID-19 dynamics to solidify claims. The method’s emphasis on stability/interpretability advances digital epidemiology.

Reviewer #2: Please see the attached file.

I am pleased to review this article as I have been involved previously in textual Big Data analytics using API-access to Google texts global data storage.

https://link.springer.com/article/10.1007/s11625-020-00889-5

This kind of study can be called API-sociology, it combines computational linguistics approach with computational social science and (or) digital epidemiology.

https://accscience.com/journal/GHES/2/1/10.36922/ghes.2148

In my opinion, this research was done well, but the results of the work have too narrow a scope of the entire spectrum of new conclusions that could have been made. The authors identified an improved list of search words compared to Google Flu Trends, and also, by applying several mathematical tools, the authors identified the most relevant cluster of words C1 for predicting the onset of a seasonal flu or COVID epidemic. And although the authors mention social behavior in the article, which is determined through text analytics, and highlight differences in word clusters in their relationship to actual disease incidence or reactions to media reports (disease-related and attention-related search activity), this analytical aspect of the study is presented sparingly in comparison with the exhaustive technical part of the article. At the current stage of text Big Data analytics and digital trace analysis, the very use of cluster analysis is not the new to scientific research. Moreover, in similar studies, various authors emphasize the possibility of combining different Big Data sources. And of particular interest is the search for new digital traces – Dark Data – that have never been analyzed before and that have for the first time demonstrated correlations relevant to the problem of epidemics.

The authors themselves state in the article that «future work should explore the integration of additional data sources, such as mobility data, syndromic surveillance, and social media signals, to enhance robustness and generalizability» of model. But the authors did not propose the extended model with other types of data. Also, the authors mention that «the inexpensive nature and scalability of this method (search words) may make it particularly useful in low-resource settings where traditional disease surveillance is limited». I don't think it's very logical to contrast digital trace analysis methods with the lack of traditional resources, since the digital traces of a growing digital infrastructure of society are not so much about cheaper analysis, but rather about fundamentally new opportunities for obtaining insights faster and with greater predictive potential than analog studies. Big digital data, its velocity, variety, and ever-growing volume, should reveal the new discoveries in the collective behavior of society. There is also a lack of a linguistic approach to analyzing the social processes behind a particular search term, which is especially important given the plasticity of language and the change of terms over time. Words can change, and processes then need to be guessed through new words.

It should be noted that this expression is not entirely correct: « The rationale being that if a cluster strongly correlates with case numbers, its terms are more likely to track actual disease burden, whereas a high correlation with media mentions suggests external information is shaping search behavior». The authors apparently meant that if a cluster strongly correlates with case numbers, its terms are more likely to actual the epidemiological burden on the population.

Overall, it is important to note the strong positive aspect of the study, which lies in the use of mathematical tools and the identification of relevant clusters of search terms. However, given the development of diverse digital infrastructure in the world and the existing complex models, a simple model with only search queries seems insufficient. The study requires major revision.

Olga Kolesnichenko, M.D., Candidate of Medical Sciences, Moscow

https://orcid.org/0000-0002-4523-6485

https://www.researchgate.net/profile/Olga-Kolesnichenko

**********

what does this mean?). If published, this will include your full peer review and any attached files.

If you choose “no”, your identity will remain anonymous but your review may still be made public.

**Do you want your identity to be public for this peer review?**  For information about this choice, including consent withdrawal, please see our Privacy Policy

Reviewer #1: No

Reviewer #2: **Yes:**  Olga Kolesnichenko, M.D., Candidate of Medical Sciences

**********

[NOTE: If reviewer comments were submitted as an attachment file, they will be attached to this email and accessible via the submission site. Please log into your account, locate the manuscript record, and check for the action link "View Attachments". If this link does not appear, there are no attachment files.]

While revising your submission, please upload your figure files to the Preflight Analysis and Conversion Engine (PACE) digital diagnostic tool, https://pacev2.apexcovantage.com/. PACE helps ensure that figures meet PLOS requirements. To use PACE, you must first register as a user. Registration is free. Then, login and navigate to the UPLOAD tab, where you will find detailed instructions on how to use the tool. If you encounter any issues or have any questions when using PACE, please email PLOS at figures@plos.org. Please note that Supporting Information files do not need this step.

Attachment Submitted filename: Review Kolesnichenko Olga.pdf

# Author comment: Author response to Decision Letter 1

**DOI:** 10.1371/journal.pone.0358063.r002

18 Nov 2025

We would like to thank the reviewer for the careful reading and recommendation to submit. Below we address the indicated weaknesses and suggestions one by one. All new text is marked in blue in the “'Revised Manuscript with Track Changes'”.

1. Cluster validation lacks empirical grounding. Claims like "C1 = symptom-driven" rely on correlations; integrate user surveys (e.g., "Did you search 'fever' due to symptoms?") to confirm behavioral intent.

The issue of empirical grounding for the clusters and the interpretation of behavioral intent is an important one and we thank the reviewer for pointing it out. Indeed, direct validation (e.g., through surveys) was not done, and we think that it carries its own limitations, such as recall bias and lack of representativeness. Instead, our validation relied on independent behavioral proxies, specifically, correlation with case counts and media coverage, which consistently distinguish clusters tracking real disease burden (C1) from those dominated by media-driven attention (C2). We reasoned that this provides a pragmatic, population-level behavioral grounding aligned with the study’s epidemiological focus.

We do agree that the previous framing risked possible over-interpretation and/or inferring behavioral intent at the individual level. Therefore, we took four main steps: (1) Clarify that cluster labels (e.g., “C1 = symptom-related”) are used solely as population-level summaries and are not intended to infer individual motives; (2) Make explicit that our approach is data-driven and descriptive rather than behavioral in the survey sense; (3) cite previous work showing that different motivations lead to different information-seeking behaviors and to different search patterns (for example, showing that symptom-related health queries are often prompted by personal or family illness, whereas searches about institutions and public measures are more associated with information exposure, risk perception, and collective concern; these patterns have been documented via query logs, surveys, and mixed-methods studies); and (4) removed possible causal phrasing and made the descriptive scope more explicit.

Specifically, in the Introduction we added:

“Beyond volume, evidence shows that both real risk and risk perception shape online health information seeking [45], and that searching behaviors differ for people who feel ill versus healthy [46, 47]. Queries about institutions or public measures, by contrast, often rise with information exposure and broader public concern. Online searching is also positively associated with intentions to take preventive measures [48, 49].”

The Results section now reads:

“Cluster labels were assigned after clustering from the most frequent terms and their temporal behavior and are used only to aid interpretation at the population level.” (...) “Still, in what follows, cluster labels are used to summarize group behavior, and should not be read as claims about individual motivations.” (...) “Different online search patterns can be explained by different offline drivers.” (...) “However, these interpretations are ad hoc; an a priori assessment would not necessarily anticipate the observed cluster structure, and these clusters cannot be used to infer individual intent.”

And the Methods Section includes:

“Cluster labels were assigned after clustering based on the most frequent terms in each cluster and their temporal behavior, and are used only to aid interpretation at the population level. These labels do not make claims about individual motives. They were not used as targets or inputs during model training and do not affect model estimation or evaluation; they serve only to describe groups and to define the C1, C2, and C3 feature sets.”

We hope that these changes address the reviewer’s concerns.

2. COVID-19 instability is underexplored. Performance dips during policy/variant shifts (e.g., Omicron); stratify analysis by pandemic phases to test if terms like "loss of smell" lose relevance post-2022.

This is a very important observation, and we appreciate the suggestion. We have now expanded the COVID-19 analysis to examine model performance and feature relevance across dominant variant phases through two complementary analyses (1) Phase-stratified performance: we report RMSE and R² within each major variant period and show a clear dip in performance during the Omicron BA.1 transition (winter 2021–2022), consistent with shifts in testing behavior and symptom perception. (2) Term-level feature relevance over time: using weekly Random Forest importances and one-tailed Mann–Whitney U tests with Benjamini–Hochberg correction, we find significant post-2022 declines for symptom-specific queries (e.g., loss of smell, loss taste), whereas general COVID-related terms (e.g., covid cases, testing) remain comparatively stable.

Together, these results support the reviewer’s prediction that model stability was disrupted during the Omicron phase and that symptom-related behaviors became less predictive in later stages of the pandemic.

This is detailed in the new Supporting information section “Temporal instability and variant-specific performance during the COVID-19 pandemic”, which includes two figures(S13–S14) and one Table (S14).

We have also referenced these additions in three sections of the revised manuscript.

The Results section now reads:

“Finally, to further examine the temporal stability of model performance, we stratified the analysis by dominant COVID-19 variant phases (see S13–S14 Figs and S14 Table in the Supporting information). An increase in RMSE was observed during the Omicron BA.1 transition (winter 2021–2022), indicating a temporary decline in predictive performance coinciding with shifts in testing behavior and symptom perception. In parallel, a longitudinal evaluation of feature importance from the Random Forest models showed that symptom-specific terms such as loss of smell and loss taste significantly declined in predictive relevance after early 2022, whereas more general COVID-related queries (e.g., covid cases, testing) remained relatively stable. These findings suggest that changes in strain dominance coincide with changes in search patterns, emphasizing the importance of continuously incorporating behavioral dynamics into nowcasting frameworks.”

In the Discussion, we have added:

“Consistent with this view, the variant-stratified analysis highlights that model performance can shift with behavior and symptom frequency (e.g., declining value of loss of smell after Omicron). This also reinforces that feature selection alone does not guarantee temporal robustness; models should incorporate continuous mechanisms for behavior-aware adaptation (e.g., monitoring feature drift and refreshing feature sets) to remain reliable in real-life settings.”

The Methods section includes a new subsection (“Variant-stratified performance and feature-importance tests”), which reads:

“For the variant-stratified analyses, the COVID-19 timeline was divided into seven non-overlapping phases using inclusive start and end dates to tag each week: Pre-Alpha (March 15, 2020 to December 13, 2020), Alpha (December 14, 2020 to June 20, 2021), Delta (June 21, 2021 to December 12, 2021), Omicron BA.1 (December 13, 2021 to March 20, 2022), Omicron BA.2 (March 21, 2022 to June 19, 2022), Omicron BA.5 (June 20, 2022 to October 16, 2022), and Omicron XBB+ (October 17, 2022 to May 7, 2023) [59]. Phase-specific performance metrics (RMSE and R2) were computed using only the weekly predictions within each phase, and a four-week rolling RMSE was also calculated to capture short-term fluctuations. To examine whether individual search terms changed in predictive relevance, weekly Random Forest feature importances from the ‘ALL’ model were used and plotted in a term-by-week heatmap with vertical lines marking the variant boundaries. For each term, we then tested whether importance decreased after January 2022 using a one-tailed Mann–Whitney U test (pre-2022 vs post-2022), applying Benjamini–Hochberg FDR correction [60] at 0.05. Terms with a significant decline are marked with an asterisk in the heatmap (S14 Fig). This procedure quantifies when specific search behaviors (e.g., ‘loss of smell’) lost predictive value as public perception and testing practices evolved.”

We believe that the new analysis strengthened a central argument of the paper, that topical knowledge and behavioral dynamics must be accounted for in reliable nowcasting.

3. Reproducibility risks from unofficial data tools. Benchmark against official Google Trends exports for high-impact periods (e.g., 2018 flu) to quantify sampling biases.

We thank the reviewer for another relevant point. Indeed, there are advantages and disadvantages of each methodology, which were not appropriately discussed. Our analysis was done at a time of unreliable official access - across different online platforms-, and we opted to avoid systems of privileged gatekeeping, such as proprietary APIs, that could change or be discontinued. Still, this choice should have been benchmarked.

Therefore, we have now compared the pytrends-based with the official Google Trends CSV exports downloaded directly from the web interface. We did this for both H1N1 (2009–2010) and COVID-19 (2020–2023), including terms from all three clusters.

We note that these two extractions differ in timing and native resolution: the original extraction was done months to years earlier and used an “extended” pipeline that combines monthly and daily pulls to construct a consistent weekly series (please see Methods); the new exports were download this month and provide weekly values directly. However, and despite these differences, the two sources closely align (S7–S8 Tables and S8–S9 Figs in the Supporting information), with correlations consistently high (median Pearson > 0.98) and with minimal mean absolute differences. This indicates that pytrends reproduces the official Google Trends signal, with negligible impact from sampling variability, retrieval date, or construction method.

Concretely, following the reviewer’s comment, the revised version of the manuscript describes these analyses in detail in the Supporting information which also includes two tables (S7 and S8 Tables - Reproducibility of Google Trends extraction for Flu and COVID-19 respectively, -pytrends vs official exports), and two figures (S8 and S9 Fig. Reproducibility: pytrends vs official exports for flu and COVID-19,, respectively).

These steps and results are referenced in the Main Manuscript, while arguing that, for a public-health tool, relying on a pipeline that performs robustly even under standard UI/API sampling noise is more sustainable than one that depends on special or unstable access.

Concretely, we added/clarified the Results section to read:

“Second, we further tested whether the results were an artifact of the data retrieval tool (pytrends), by comparing these time series with the official Google Trends CSV exports (two queries per cluster). For all tested terms, correlations were very high (all Pearson and Spearman coefficients > 0.94; S7 Table for flu and S8 Table for COVID-19), and temporal profiles were similar (S8 and S9 Figs). These controls confirm the reliability of the automated pipeline in producing consistent estimates of search interest over time, supporting its use for epidemiological applications that require periodic data updates.”

The Methods now also include:

“To verify consistency, we benchmarked pytrends outputs against manual downloads from the Google Trends website for the same queries, locations, and dates (see S8–S9 Figs in the Supporting information).”

And the Discussion has the following reproducibility note:

“Finally, for public health use, analysis must remain consistent and reproducible. A method that relies on private platform data, as is the case of Google Trends and its API, risks interruptions or instability from changing access paths, shifting policies, quota limits, pricing, and even basic availability. Although interface sampling can introduce minor run-to-run variation, it can minimize some of these issues. In this work, repeated exports show that comparative results remain stable (see S8–S9 Figs in the Supporting information), and preference should be given to pipelines resilient to platform variability. Still, they do not solve the fundamental problem of relying on non-public data.”

4. Linear Regression superiority unexplained. Diagnose Random Forest failures during outbreaks (e.g., 2018 flu overprediction) via residual analysis to justify simplicity.

Another important point, one that we discussed internally and debated whether to include in the first submission. We have now attempted to clarify how and why the linear regression model outperforms the random forest. Specifically, what we observe (and what is expected from previous work, new references [61-63]), is an underprediction by the random forest when the test dataset includes values that are outside the range of values in the training dataset. In other words, the expectation is that the random forest fails in periods of very high intensity. We performed these comparisons and the results are shown in the supplementary figures S4 (flu) and S7 (COVID-19). S4 shows that the errors of the random forest model are especially large when incidence is high, while the linear regression residuals do not depend on incidence so much. In the case of COVID-19, both models have difficulties predicting high incidence values (figure S7). We offer possible explanations in the manuscript.

The Results section now reads, regarding the flu predictions: “Overall, Linear Regression outperformed Random Forest in five out of six test cases, particularly when the validation period included severe flu seasons (i.e., seasons with high peaks of reported cases), with 2018 being the most glaring example. This can be explained by the large differences in amplitude between training and testing data. Random forests perform regression tasks by building trees where each branch partitions the dependent variable arbitrarily [61]. Since each tree will have its own cutoffs, this makes the models very good at tackling non-linear problems. However, they cannot learn any partition beyond the range present in the training data. If the features point to a very high (low) value, then the trained model will predict the highest (lowest) value present in the training data. If so, the expectation is that Random Forest will perform the poorest when the highest or lowest values were not present in the training data. Indeed, this is what S3 Fig shows, and has been observed by others (e.g. [62, 63]): the only exception to the Random Forest under-performance was P6, for which the test period did not include the unusually intense 2018 flu outbreak. Model performance and full comparisons between observed and predicted cases for each period are provided in S4 Table and S2–S4 Figs in the Supporting information.”

And regarding the COVID-19 predictions: “In this case, the Linear Regression models also outperformed the Random Forest, but likely for different reasons than in the case of the flu. Here, both models are equally poor at forecasting the largest peak (figures S5, S6 and S7), but the Random Forest predictions are generally worse, while the Linear Regression fails less consistently. One possible explanation is the ARGO model using past predictions as a feature, while the Random Forest does not include such auto-correlation inputs.”

5. Ethical gaps. Add brief guidance on privacy (e.g., aggregate geographic data) and digital-access equity to ensure responsible deployment.

This is a very important note, and we apologize for the oversight.

The following text has been added to the top of the Methods section:

“Health data is considered sensitive under the EU GDPR. In this study only previously anonymized and aggregated data was used.”

Under section “Search Trend data” we have added:

“Data extracted from GT returns the normalized, scaled and aggregated total number of searches for a particular query, in a specified geographical region and time range. All data is anonymous and it is virtually impossible to identify individual searchers.”

In the Discussion section we have added:

“Indeed, if deployed responsibly, this method also offers promise to help reduce health inequities by providing rapid insights. Curated search terms, if adjusted for local behaviors and disparities in digital access, can improve disease surveillance without reinforcing existing digital divides.”

Again, we thank the Reviewer for the thoughtful comments, believe to have addressed all concerns, and find that our works was greatly strengthened by the revision.

Attachment Submitted filename: PLoSONE_Response to Reviewers.pdf

# Peer review: Decision Letter 1

**DOI:** 10.1371/journal.pone.0358063.r003

**Authors:** Kyungwon Kim

8 Jun 2026

Dear Dr. Gonçalves-Sá,

Thank you for submitting your manuscript to PLOS ONE. After careful consideration, we feel that it has merit but does not fully meet PLOS ONE’s publication criteria as it currently stands. Therefore, we invite you to submit a revised version of the manuscript that addresses the points raised during the review process.

==============================

**ACADEMIC EDITOR:**

As the Academic Editor, I would like to commend you on your revised manuscript. Reviewers #4 and #5 are fully satisfied with your revisions, and Reviewer #6 has provided a few constructive suggestions to further polish your work.

I am recommending a **Minor Revision** . Please address the following points in your next submission:

**1. Addressing Reviewer #6's Comments (Required):**  Please carefully review and incorporate the valuable suggestions from Reviewer #6. Specifically:

- Consider the suggestion regarding the manuscript title (e.g., using "Feature Clustering" instead of "Feature Selection") to better reflect your methodology, if you find it viable.
- Expand your Discussion section to address how redundancy among searched terms might threaten nowcasting accuracy and how this bias-variance issue could be technically addressed in future implementations.

**2. Academic Editor's Note Regarding Reviewer #3 (Overruled):**  Please note that I have carefully evaluated the extensive requests made by Reviewer #3. The demands to implement entirely new suites of machine learning models (e.g., CatBoost, XGBoost, AdaBoost, etc.), introduce numerous new statistical metrics, and mandatorily cite 15+ specific papers fall outside the original scope of your study and are disproportionate for this stage of revision.

- **Action:**  You are **NOT required**  to perform new experiments, add these new algorithms/metrics, or cite the specific papers requested by Reviewer #3. You may briefly address their structural comments (e.g., clarifying the abstract/introduction) if you find it helpful for the manuscript's flow, but you may respectfully decline the methodological and heavy citation demands in your rebuttal letter.

**3. Data Availability Verification:**  Although Reviewer #5 approved the paper, they marked "No" for Data Availability. Please double-check that your data availability statement fully complies with PLOS ONE's policies and that all underlying data are accessible without restriction.

Please submit a "Response to Reviewers" letter detailing how you have addressed Reviewer #6's points.

==============================

Please submit your revised manuscript by Jul 23 2026 11:59PM. If you will need more time than this to complete your revisions, please reply to this message or contact the journal office at plosone@plos.org. When you're ready to submit your revision, log on to https://www.editorialmanager.com/pone/ and select the 'Submissions Needing Revision' folder to locate your manuscript file.

Please include the following items when submitting your revised manuscript:

- A letter that responds to each point raised by the academic editor and reviewer(s). You should upload this letter as a separate file labeled 'Response to Reviewers'.
- A marked-up copy of your manuscript that highlights changes made to the original version. You should upload this as a separate file labeled 'Revised Manuscript with Track Changes'.
- An unmarked version of your revised paper without tracked changes. You should upload this as a separate file labeled 'Manuscript'.

If you would like to make changes to your financial disclosure, please include your updated statement in your cover letter. Guidelines for resubmitting your figure files are available below the reviewer comments at the end of this letter.

If applicable, we recommend that you deposit your laboratory protocols in protocols.io to enhance the reproducibility of your results. Protocols.io assigns your protocol its own identifier (DOI) so that it can be cited independently in the future. For instructions see: https://journals.plos.org/plosone/s/submission-guidelines#loc-laboratory-protocols. Additionally, PLOS ONE offers an option for publishing peer-reviewed Lab Protocol articles, which describe protocols hosted on protocols.io. Read more information on sharing protocols at https://plos.org/protocols?utm_medium=editorial-email&utm_source=authorletters&utm_campaign=protocols.

As the corresponding author, your ORCID iD is verified in the submission system and will appear in the published article. PLOS supports the use of ORCID, and we encourage all coauthors to register for an ORCID iD and use it as well. Please encourage your coauthors to verify their ORCID iD within the submission system before final acceptance, as unverified ORCID iDs will not appear in the published article. *Only*  the individual author can complete the verification step; PLOS staff *cannot*  verify ORCID iDs on behalf of authors.

We look forward to receiving your revised manuscript.

Kind regards,

Kyungwon Kim

Academic Editor

PLOS One

Journal Requirements:

1. If the reviewer comments include a recommendation to cite specific previously published works, please review and evaluate these publications to determine whether they are relevant and should be cited. There is no requirement to cite these works unless the editor has indicated otherwise.

2. Please review your reference list to ensure that it is complete and correct. If you have cited papers that have been retracted, please include the rationale for doing so in the manuscript text, or remove these references and replace them with relevant current references. Any changes to the reference list should be mentioned in the rebuttal letter that accompanies your revised manuscript. If you need to cite a retracted article, indicate the article’s retracted status in the References list and also include a citation and full reference for the retraction notice.

[Note: HTML markup is below. Please do not edit.]

Reviewers' comments:

Reviewer's Responses to Questions

**Comments to the Author**

Reviewer #3: (No Response)

Reviewer #4: All comments have been addressed

Reviewer #5: All comments have been addressed

Reviewer #6: All comments have been addressed

**********

2. Is the manuscript technically sound, and do the data support the conclusions?

Reviewer #3: No

Reviewer #4: Yes

Reviewer #5: Yes

Reviewer #6: Yes

**********

3. Has the statistical analysis been performed appropriately and rigorously?

Reviewer #3: No

Reviewer #4: Yes

Reviewer #5: Yes

Reviewer #6: Yes

**********

4. Have the authors made all data underlying the findings in their manuscript fully available?

The PLOS Data policy

Reviewer #3: No

Reviewer #4: Yes

Reviewer #5: No

Reviewer #6: Yes

**********

5. Is the manuscript presented in an intelligible fashion and written in standard English?

Reviewer #3: No

Reviewer #4: Yes

Reviewer #5: Yes

Reviewer #6: Yes

**********

Reviewer #3: The paper titled “Learning from Pandemics: Feature Selection for Robust Digital Disease Nowcasting” requires essential improvements. Some sections of the present version of the revision remained unclear and then reviewer is worrying about scientific and originality of the paper contents.

1-In abstract section, authors need to mention which Machine Learning Models (MLMs) were used in this study with their main purposes. The main findings along with its conditions of datasets can be merged to the abstract section.

2-Introduction section requires diverse related research works, describing application of MLMs in the case of feature selection and capability of robust MLMs. Reviewer is now considering table containing literature review in this area whereas this table need a lot of major modifications. Authors need to consider attachments and those highlights were indicated in the paper.

3-Research gap and novelty should be deeply discussed in the introduction section.

4-Research organization, known as the last paragraph of introduction section should be deeply provided.

5- Delete subsections in the introduction section. And more, all the subsections should have had only single name as “Introduction” in which includes (i) importance of subject and background theorem, (ii) literature review with emphasize on major drawbacks of empirical/traditional methodologies equations, (iii) research gap and novelty, and (iv) research organization in which overview of the present research should be mentioned in a paragraph.

6-Details of case study and some important discussions on the datasets should be detailed.

7-Authors are emphatically recommended to use MLMs such as KNN, Model Tree (MT), AdaBoost, XGBoost, CatBoost, LightBoost, and RF for the training and testing stages.

9-Descriptions on every MLM should be presented in a separate subsection along with the setting parameters of each predictive models and the corresponding justifications. In this section, authors should use AdaBoost, CatBoost, LighGBoost, XGBoost, KNN, SVM, RF, and other regression models based on machine learning techniques (ElasNet, Lasso, and Ridge).

10- Instead using R2, authors are emphatically recommended to use Correlation of Coefficient (CC), RMSE, Mean Absolute Error (MAE), Mean Bias Error (MBE), Discrepancy Ratio (DR), and Scatter Index (SI) to this section. These statistical measures should be computed for the training and testing stages of the present predictive models. All the formulations and acceptable ranges of these statistical measures should be merged to this section. For this purpose, read the following papers and add them to the present revisions.

11-Use Scatter plots, Taylor’s diagrams, heat maps, and violin plots to illustrate the results of the training and testing stages.

12-The present results should be compared with those related papers (use more than 15 papers at least with quantitative and qualitative comparisons).

Reviewer #4: (No Response)

Reviewer #5: The previous reviewers’ comments have been addressed, and I believe the paper is acceptable.

Reviewer #6: Based on the manuscript contents and contexts, all the above questions seem to fulfill the PLOS requirements. However, there are a few comments to the authors for consideration, which include:

1. The manuscript title is "Learning from Pandemics: Feature Selection for Robust Digital Disease Nowcasting." Discussions are focused on how to improve prediction that combines model evaluation with behavioral insight using curated features. Since hierarchical clustering is applied in data processing and the clustering results (C1, C2, C3) become the input for the rest of the analysis for 2009 H1N1, while for Covid-19 the predefined period (P1, P2, ..., P6) is applied, I would suggest revising the title to use "Feature Clustering" instead. For the "Feature Selection" to be used, discussions shall highlight how the number of searched terms is reduced in quantity (leaving only the significant terms) that would help improve the prediction. Consider this if it is viable.

2. Searched terms in the online may be an intercept between different aspects of symptoms, treatment, personal care, institutions, and pandemic-specific terms. As this led to redundancy, how does this study see this as a threat to the accuracy of nowcasting? Add your comments in the discussion if it has not been addressed.

3. Redundancy among the searched terms is seen to be the source of bias-variance. For future nowcasting implementation, how is this issue to be addressed technically to increase predictability and accuracy? Include this in the discussion.

**********

what does this mean?). If published, this will include your full peer review and any attached files.

If you choose “no”, your identity will remain anonymous but your review may still be made public.

**Do you want your identity to be public for this peer review?**  For information about this choice, including consent withdrawal, please see our Privacy Policy

Reviewer #3: No

Reviewer #4: No

Reviewer #5: No

Reviewer #6: No

**********

[NOTE: If reviewer comments were submitted as an attachment file, they will be attached to this email and accessible via the submission site. Please log into your account, locate the manuscript record, and check for the action link "View Attachments". If this link does not appear, there are no attachment files.]

To ensure your figures meet our technical requirements, please review our figure guidelines: https://journals.plos.org/plosone/s/figures

You may also use PLOS’s free figure tool, NAAS, to help you prepare publication quality figures: https://journals.plos.org/plosone/s/figures#loc-tools-for-figure-preparation.

NAAS will assess whether your figures meet our technical requirements by comparing each figure against our figure specifications.

Attachment Submitted filename: Comments on PONE-2026-28523.docx

Attachment Submitted filename: Comments on PONE-2026-28523.pdf

# Author comment: Author response to Decision Letter 2

**DOI:** 10.1371/journal.pone.0358063.r004

10 Jul 2026

We thank the Academic Editor and the reviewers for the time and effort invested in the review process and for their relevant comments and suggestions. We have carefully considered all comments and have revised the manuscript accordingly. Below, in blue, we provide a detailed, point-by-point response to each comment. Changes made to the manuscript are indicated in italics throughout this document and in blue in the manuscript.

Reviewer 3

The paper titled “Learning from Pandemics: Feature Selection for Robust Digital Disease Nowcasting” requires essential improvements. Some sections of the present version of the revision remained unclear and then reviewer is worrying about scientific and originality of the paper contents.

We thank R3 for the detailed comments and while we agree with several suggestions we respectfully try to justify why some others might be outside of the scope of the current work.

1-In abstract section, authors need to mention which Machine Learning Models (MLMs) were used in this study with their main purposes. The main findings along with its conditions of datasets can be merged to the abstract section.

To comply both with the reviewer’s request and with the journal’s word formatting requirements, the following sentence has been added to the abstract (in italics):

“We propose a hybrid approach to improve prediction that combines model evaluation with behavioral insight. We apply simple supervised and unsupervised methods to different flu and COVID-19 datasets, using the 2009 H1N1 and 2020 COVID-19 pandemics as training periods. In both cases, selecting a curated subset of search terms improved model performance, reduced overfitting, and increased stability, consistently outperforming models that used search terms indiscriminately.”

2-Introduction section requires diverse related research works, describing application of MLMs in the case of feature selection and capability of robust MLMs. Reviewer is now considering table containing literature review in this area whereas this table need a lot of major modifications.

For Feature Selection: -Unsupervised feature selection via graph-based proximity and structured autoencoder-like NMF -Joint sample-feature subspace learning via bidirectional reconstruction for feature selection For robustness: -Robust oblique projection and weighted NMF for hyperspectral unmixing -Simultaneous outlier detection and elimination in hyperspectral unmixing via weighted non-negative matrix tri-factorization

We thank the reviewer for these suggestions and carefully considered the recommended references. While some of these articles fall outside the scope of the present study, as the manuscript focuses on the application of machine learning methods to digital epidemiology and disease nowcasting rather than on methodological developments in machine learning algorithms, we see how the paper could benefit from a broader overview of recent work in this area. Accordingly, we have updated it with references covering (i) applications of machine learning in epidemiology, (ii) recent developments in feature selection methodologies, and (iii) recent advances in infectious disease forecasting using digital data. In particular, we have added:

1 - LLM-Assisted Keyword Curation for Early Disease Outbreak Detection Using Search Trends, by Brahmos and Princess (2025), which reinforces the importance of careful feature selection in search-based epidemiology and provides a complementary, LLM-assisted approach to keyword curation.

Added to the Introduction (Lines 19-23): "As the use of online search data for disease surveillance has matured, attention has shifted from whether these data are useful to how the most informative search terms should be selected. Even with recent advances in artificial intelligence, careful keyword curation remains essential for identifying the search terms that carry the most relevant information for disease prediction [19].”

2 - “Machine learning in epidemiology: An introduction, comparison with traditional methods, and a case study of predicting extreme longevity”, a recent review by Goldstein et al., which discusses the application of machine learning in epidemiology and reinforces the importance of selecting methods according to the epidemiological question being addressed

Added to the Discussion (Lines 663–665): “This is consistent with recent perspectives arguing that, in epidemiology, machine learning methods should be selected according to the question being addressed rather than solely on predictive performance [74].”

3 – “Mathematical methods in feature selection: A review”, by Kamalov et al. (2025), which classifies feature selection methods according to their mathematical foundations and discusses their respective strengths, limitations, and areas of application, providing additional context for the approaches evaluated in the present study.

4 – “Recent advances in feature selection and its applications”, by Li et al., which reviews recent developments in feature selection and highlights the importance of selecting relevant and non-redundant features to improve both predictive performance and model interpretability. This provides additional theoretical context for the behaviourally informed feature selection strategy proposed in the present study.

Citations 3 and 4 were added to the Introduction (Lines 88—90): "Recent reviews have emphasized that effective feature selection should identify informative predictors while reducing redundancy, thereby improving predictive performance, robustness, and model interpretability [52, 53].”

5 - Advancing real-time infectious disease forecasting using large language models, by Du et al. (2025), which provides a recent overview of advances in infectious disease forecasting using artificial intelligence and highlights the growing integration of digital and heterogeneous data sources to complement traditional surveillance systems.

Added to the Introduction (Lines 7--10): "As surveillance mechanisms relying solely on highly trained personnel are often expensive, limited, and slow, it has been extensively argued that traditional methods should be complemented with 'Digital Era' tools, such as online activity, mobility patterns, or digital contact-tracing [1, 2, 3, 4]."

6 - Forecasting dengue cases through time-series modeling with Google

Trends and deep neural networks, by Cheong et al. (2025), which demonstrates that parsimonious Google Trends feature sets can outperform larger collections of search terms in infectious disease forecasting. While their feature selection is based on statistical associations,

our work provides a complementary behavioural perspective that helps explain why the indiscriminate inclusion of search terms may reduce predictive performance.

Added to the Results (Lines 507--509): “This is consistent with recent work on dengue forecasting using Google Trends, where parsimonious search-term sets selected through statistical associations outperformed larger collections of search terms [72].”

3-Research gap and novelty should be deeply discussed in the introduction section.

We agree that clearly defining the research gap and novelty is important. To address this comment, we revised the final paragraph of the Introduction to more clearly distinguish our contribution from previous work. Specifically, we now explicitly identify the research gap and clarify the novelty of the proposed approach. The Introduction now reads:

“Recent reviews have emphasized that effective feature selection should identify informative predictors while reducing redundancy, thereby improving predictive performance, robustness, and model interpretability. However, relatively little attention has been paid to whether pandemic periods themselves can be exploited to improve feature selection. To address this gap, we propose a simple and explainable feature selection framework that leverages the high-intensity periods of pandemics as natural experiments to identify search terms with greater temporal robustness for disease nowcasting.”(Lines 88–95).

4-Research organization, known as the last paragraph of introduction section should be deeply provided.

To address this comment, we expanded the final paragraph of the Introduction to include a brief overview of the organization of the manuscript. After introducing the research gap and the proposed method, we now briefly describe how the study is structured, from the presentation of the data sources and modelling approach to the evaluation of the proposed method and the discussion of its implications.

The Introduction now concludes with:

"To evaluate this approach, we first describe the data sources and modelling approach, then assess its performance across influenza and COVID-19 case studies, and finally discuss its implications for the design of robust and interpretable disease nowcasting systems." (Lines 98-101)

5- Delete subsections in the introduction section. And more, all the subsections should have had only single name as “Introduction” in which includes (i) importance of subject and background theorem, (ii) literature review with emphasize on major drawbacks of empirical/traditional methodologies equations, (iii) research gap and novelty, and (iv) research organization in which overview of the present research should be mentioned in a paragraph.

We ask the reviewer to confirm whether the current version of the manuscript complies with that request or offer more detail on how it should be improved.

6-Details of case study and some important discussions on the datasets should be detailed.

We ask the reviewer to confirm whether the current version of the manuscript complies with that request or offer more detail on how it should be improved.

7-Authors are emphatically recommended to use MLMs such as KNN, Model Tree (MT), AdaBoost, XGBoost, CatBoost, LightBoost, and RF for the training and testing stages.

Algorithms such as KNN, Model Trees, AdaBoost, XGBoost, CatBoost, and LightGBM are indeed powerful machine learning methods and can provide great predictive performance in many applications. However, since the primary objective of the present study was to evaluate whether behaviourally informed feature selection improves the robustness and generalizability of disease nowcasting models, we decided against testing a broad range of models and selected two complementary and widely used approaches: Linear Regression, as a simple and fully interpretable baseline, and Random Forest, as a nonlinear ensemble method capable of capturing more complex relationships.

The fact that the proposed feature selection strategy consistently improved performance across both model classes suggests that the observed gains arise from the quality of the selected features and not from a particular algorithm. While evaluating additional algorithms such as gradient boosting methods would certainly be an interesting direction for future work, we respectfully argue that it falls outside the scope of the present study and would not substantially alter its main conclusions.

9-Descriptions on every MLM should be presented in a separate subsection along with the setting parameters of each predictive models and the corresponding justifications. In this section, authors should use AdaBoost, CatBoost, LighGBoost, XGBoost, KNN, SVM, RF, and other regression models based on machine learning techniques (ElasNet, Lasso, and Ridge).

To improve this section, authors need to use the material of the following papers:

- Vulnerability of the rip current phenomenon in marine environments using machine learning models

- Assessment of flood susceptibility in Minab County, Iran, through the integration of topographic, climatic, and land-surface indices using ensemble machine learning models

- Ultrasonic flowmeter fault early warning method based on Bayesian optimization XGBoost

- Evaluation of river water quality index using remote sensing and artificial intelligence models

- A Stacked Machine Learning Algorithm for Multi-Step Ahead Prediction of Soil Moisture

Please see our response to Comment 7 regarding the rationale for the selection of the predictive models evaluated in this study, as the same considerations apply here.

10- Instead using R2, authors are emphatically recommended to use Correlation of Coefficient (CC), RMSE, Mean Absolute Error (MAE), Mean Bias Error (MBE), Discrepancy Ratio (DR), and Scatter Index (SI) to this section. These statistical measures should be computed for the training and testing stages of the present predictive models. All the formulations and acceptable ranges of these statistical measures should be merged to this section. For this purpose, read the following papers and add them to the present revisions.

-A hybrid hourly natural gas demand forecasting method based on the integration of wavelet transform and enhanced Deep-RNN model

-A deep reinforcement learning-based method for predictive management of demand response in natural gas pipeline networks

-A Combinatorial Machine Learning Models -Based Method for Predicting the Viscosity-Temperature Relationship of Crude Oil

-Receiving More Accurate Predictions for Longitudinal Dispersion Coefficient in Water Pipelines: Training Group Method of Data Handling Using Extreme Learning Machine Conceptions

-A Method for Predicting the Gel Point of Mixed Crude Oil Based on Deep Learning

- A Large Language Model Based Knowledge Mining Method for Improving the Reliability of Fire Water Systems

- Optimization Method of Natural Gas Pipeline Network Emergency Dispatch Based on Particle Swarm Optimization

-Data-driven reliability assessment method of Integrated Energy Systems based on probabilistic deep learning and Gaussian mixture Model-Hidden Markov Model

-Interpretable Combinatorial Machine Learning-Based Shale Fracability Evaluation Methods

-Dynamic Prediction of Natural Gas Calorific Value Based on Deep Learning

-Water quality monitoring for coastal hypoxia: Integration of satellite imagery and machine learning models

-Dynamic damage functions for scour protection at mono-pile foundations: Application of ensemble machine learning models

-A new correlation for calculating carbon dioxide minimum miscibility pressure based on multi-gene genetic programming

We ask the reviewer to confirm whether the current version of the manuscript complies with that request. In addition to R2, and partially also in response to reviewer R1, we added bias-variance decomposition (Methods, Bias-variance trade-off subsection), and RMSE-based analyses, including phase-specific and rolling RMSE evaluations (Methods, Variant-stratified evaluation subsection; Results, Overfitting reduction, generalization, and predictive reliability in feature selection subsection; S14 Table).

11-Use Scatter plots, Taylor’s diagrams, heat maps, and violin plots to illustrate the results of the training and testing stages.

We agree that visual assessment is very useful when evaluating models. In the current manuscript and Supporting Information, we already provide several complementary visualizations of model performance, including line plots comparing observed and predicted trajectories for both Linear Regression and Random Forest models in the flu and COVID-19 analyses (S2–S3 and S5–S6 Figs), scatter plots relating residuals to observed incidence, line plots showing the temporal evolution of residuals, histograms illustrating residual and incidence distributions, and binned summary plots of mean residuals across incidence levels (S4 and S7 Figs). In the main manuscript, we also compare model performance across feature sets (Fig. 2). In addition, the manuscript includes heatmaps illustrating the longitudinal evolution of feature importance across SARS-CoV-2 variant periods, as requested by another reviewer.

It is our assessment that the current set of figures provides sufficient visual information to evaluate model performance and prediction errors across the training and testing settings. While Taylor diagrams and violin plots could provide an alternative way of visualizing some of these results, we are not sure that they would provide additional insight. If the reviewer feels that a particular aspect of model performance would benefit from additional visualization, we would appreciate more specific clarification so that we can better understand the concern and address it as effectively as possible.

12-The present results should be compared with those related papers (use more than 15 papers at least with quantitative and qualitative comparisons).

Please see answer to comment 2.

Reviewer #6:

Based on the manuscript contents and contexts, all the above questions seem to fulfill the PLOS requirements.

We thank the reviewer for the positive and constructive comments and overall recommendation to publish.

However, there are a few comments to the authors for consideration, which include: 1. The manuscript title is "Learning from Pandemics: Feature Selection for Robust Digital Disease Nowcasting." Discussions are focused on how to improve prediction that combines model evaluation with behavioral insight using curated features. Since hierarchical clustering is applied in data processing and the clustering results (C1, C2, C3) become the input for the rest of the analysis for 2009 H1N1, while for Covid-19 the predefined period (P1, P2, ..., P6) is applied, I would suggest revising the title to use "Feature Clustering" instead. For the "Feature Selection" to be used, discussions shall highlight how the number of searched terms is reduced in quantity (leaving only the significant terms) that would help improve the prediction. Consider this if it is viable.

We thank the reviewer for the comment. It is not only viable, but it also offers a better description of the work. We have updated the title as suggested.

2. Searched terms in the online may be an intercept between different aspects of symptoms, treatment, personal care, institutions, and pandemic-specific terms. As this led to redundancy, how does this study see this as a threat to the accuracy of nowcasting? Add your comments in the discussion if it has not been addressed.

We agree both with the note that search terms can have different natures and underlying motivations, and that redundancy is often a problem. We cluster similar terms and include two types of analysis (in the supplementary materials) in line with this point: LASSO regression and performance when selecting terms by correlation (pandemic and seasonal periods). We now also mention this in the discussion, as suggested.

It reads: (Lines 641–654) “In fact, including all available features can introduce variance that overwhelms the true signal. Instead, our approach prioritizes coherence, identifying subsets of search terms that behave consistently during outbreaks (clusters) and remain predictive beyond crisis contexts (...) That these pandemic-identified terms (particularly those in C1) continue to outperform others across seasonal flu periods suggests they capture underlying behavioral regularities that are robust across time. This finding stands in contrast to purely statistical feature selection methods, such as correlation filtering or LASSO regression, which may perform well within narrow windows but often yield unstable and inconsistent results across datasets”.

3. Redundancy among the searched terms is seen to be the source of bias-variance. For future nowcasting implementation, how is this issue to be addressed technically to increase predictability and accuracy? Include this in the discussion.

This is a very relevant point and in line with a previous comment from reviewer R1. We have added the following sentence to the discussion (in italics): “This also reinforces that feature selection alone does not guarantee temporal robustness; as search behaviour evolves, maintaining temporal robustness will require approaches that monitor changes in search patterns (e.g., feature drift) and rely on domain expertise for feature interpretation and validation.” (Lines 658-660)

We also clarify that redundancy can be a source of bias-variance and the results section also reads (new text in italics): “when including too many or redundant predictors, models learn patterns that do not generalize beyond the training data” (Lines 466-467)

Again, we thank both the Editors and the Reviewers for their comments and hope that our answers address all the points raised during the review process.

Attachment Submitted filename: PLOS_One_Response_to_Reviewers.pdf

# Peer review: Decision Letter 2

**DOI:** 10.1371/journal.pone.0358063.r005

**Authors:** Kyungwon Kim

10 Aug 2026

Dear Dr. Gonçalves-Sá,

Thank you for submitting your manuscript to PLOS ONE. After careful consideration, we feel that it has merit but does not fully meet PLOS ONE’s publication criteria as it currently stands. Therefore, we invite you to submit a revised version of the manuscript that addresses the points raised during the review process.

==============================

**ACADEMIC EDITOR:**

As the Academic Editor, I am pleased to inform you that your manuscript is now extremely close to acceptance.

Reviewer #6 has confirmed that your previous revisions were satisfactory and has now only provided a list of minor typographical, punctuation, and formatting corrections, along with a constructive suggestion for your title.

Regarding Reviewer #3's response: As explicitly communicated in the previous decision letter, I have already overruled Reviewer #3's extensive methodological and citation demands. Reviewer #3's current refusal to review is a reaction to that editorial decision. **You may completely disregard Reviewer #3's comments in this round. No further action is required on your part regarding Reviewer #3.**

Therefore, I am recommending a final **Minor Revision** . To proceed directly to formal acceptance, please address the following specific points raised by Reviewer #6:

**1. Title Modification:**  Please consider Reviewer #6's suggestion to update the title to: *"Learning from Pandemics: Feature Selection and Clustering for Robust Digital Disease Nowcasting."*  to better reflect your complete methodology.

**2. Typographical and Formatting Corrections:**  Please carefully go through the 18 specific minor corrections listed by Reviewer #6 (e.g., fixing straight vs. smart quotes, italicizing mathematical notations like *R²* , correcting spacing, and fixing typo symbols like <code data-index-in-node="263" data-path-to-node="15">¿</code>).

Please submit a brief "Response to Reviewers" letter confirming that these typographical corrections have been made. I look forward to receiving your prompt revision and formally accepting your manuscript.

==============================

Please submit your revised manuscript by Sep 24 2026 11:59PM. If you will need more time than this to complete your revisions, please reply to this message or contact the journal office at plosone@plos.org. When you're ready to submit your revision, log on to https://www.editorialmanager.com/pone/ and select the 'Submissions Needing Revision' folder to locate your manuscript file.

- A letter that responds to each point raised by the academic editor and reviewer(s). You should upload this letter as a separate file labeled 'Response to Reviewers'.
- A marked-up copy of your manuscript that highlights changes made to the original version. You should upload this as a separate file labeled 'Revised Manuscript with Track Changes'.
- An unmarked version of your revised paper without tracked changes. You should upload this as a separate file labeled 'Manuscript'.

If you would like to make changes to your financial disclosure, please include your updated statement in your cover letter. Guidelines for resubmitting your figure files are available below the reviewer comments at the end of this letter.

If applicable, we recommend that you deposit your laboratory protocols in protocols.io to enhance the reproducibility of your results. Protocols.io assigns your protocol its own identifier (DOI) so that it can be cited independently in the future. For instructions see: https://journals.plos.org/plosone/s/submission-guidelines#loc-laboratory-protocols. Additionally, PLOS ONE offers an option for publishing peer-reviewed Lab Protocol articles, which describe protocols hosted on protocols.io. Read more information on sharing protocols at https://plos.org/protocols?utm_medium=editorial-email&utm_source=authorletters&utm_campaign=protocols.

As the corresponding author, your ORCID iD is verified in the submission system and will appear in the published article. PLOS supports the use of ORCID, and we encourage all coauthors to register for an ORCID iD and use it as well. Please encourage your coauthors to verify their ORCID iD within the submission system before final acceptance, as unverified ORCID iDs will not appear in the published article. *Only*  the individual author can complete the verification step; PLOS staff *cannot*  verify ORCID iDs on behalf of authors.

We look forward to receiving your revised manuscript.

Kind regards,

Kyungwon Kim

Academic Editor

PLOS One

Journal Requirements:

1. If the reviewer comments include a recommendation to cite specific previously published works, please review and evaluate these publications to determine whether they are relevant and should be cited. There is no requirement to cite these works unless the editor has indicated otherwise.

2. Please review your reference list to ensure that it is complete and correct. If you have cited papers that have been retracted, please include the rationale for doing so in the manuscript text, or remove these references and replace them with relevant current references. Any changes to the reference list should be mentioned in the rebuttal letter that accompanies your revised manuscript. If you need to cite a retracted article, indicate the article’s retracted status in the References list and also include a citation and full reference for the retraction notice.

[Note: HTML markup is below. Please do not edit.]

Reviewers' comments:

Reviewer's Responses to Questions

**Comments to the Author**

Reviewer #3: (No Response)

Reviewer #6: All comments have been addressed

**********

2. Is the manuscript technically sound, and do the data support the conclusions?

Reviewer #3: Yes

Reviewer #6: Yes

**********

3. Has the statistical analysis been performed appropriately and rigorously?

Reviewer #3: No

Reviewer #6: N/A

**********

4. Have the authors made all data underlying the findings in their manuscript fully available?

The PLOS Data policy

Reviewer #3: Yes

Reviewer #6: Yes

**********

5. Is the manuscript presented in an intelligible fashion and written in standard English?

Reviewer #3: Yes

Reviewer #6: Yes

**********

Reviewer #3: I can not review the manuscript. Authors can reconsider comments in the previous round of revision..

Reviewer #6: After careful consideration and evaluation based on the updated manuscript with regards to my suggestion to modify the title of your publication, I would recommend using "Feature Selection and Clustering" as your highlight. Your suggested new title will be "Learning from Pandemics: Feature Selection and Clustering for Robust Digital Disease Nowcasting."

Correction on punctuations:

1. Line 9: … -”-Digital Era” … please use the correct punctuation for -”-

2. Line 132: … -’-TrendReq’ … please choose the correct punctuation

3. Line 145: … -”-en-us” … please choose the correct punctuation for -”-

4. Line 143: … -’-related queries’ … please choose the correct punctuation for -'-

5. Line 150: … -’-flu’ or ’influenza’ … please choose the correct punctuations for -'-

6. Line 154: … -”-COVID-19” … please choose the correct punctuation for -”-

7. Line 182: … ’--Extended Google Queries’ … please choose the correct punctuation for -'-

8. Line 247: … -”-auto”, -”-sqrt” … please choose the correct punctuation for -”-

9. Lines 277, 282, 395, 413, 419, 599, 601, 602, page 18: -R-² please italicized mathematical notation

10. Line 28: … -”-All” … please choose the correct punctuation for -”-

11. Line 376: … clusters_, … delete unintended space before comma

12. Line 387: … -”-true signal” … please choose the correct punctuation for -”-

13. Line 417: … -”-All” … please choose the correct punctuation for -”-

14. Line 450: GOogle search data) … does the “O” capitalized?

15. Page 13 (Fig. 2): … -”-All” … please choose the correct punctuation for -”-

16. Line 471: … models.To test … add space after fullstop

17. Line 547: … (R2 ¿ 0.6) … please check whether the symbol is correct ¿

18. Line 549: … -”-All” … please choose the correct punctuation for -”-

**********

what does this mean?). If published, this will include your full peer review and any attached files.

If you choose “no”, your identity will remain anonymous but your review may still be made public.

**Do you want your identity to be public for this peer review?**  For information about this choice, including consent withdrawal, please see our Privacy Policy

Reviewer #3: No

Reviewer #6: No

**********

[NOTE: If reviewer comments were submitted as an attachment file, they will be attached to this email and accessible via the submission site. Please log into your account, locate the manuscript record, and check for the action link "View Attachments". If this link does not appear, there are no attachment files.]

To ensure your figures meet our technical requirements, please review our figure guidelines: https://journals.plos.org/plosone/s/figures

You may also use PLOS’s free figure tool, NAAS, to help you prepare publication quality figures: https://journals.plos.org/plosone/s/figures#loc-tools-for-figure-preparation.

NAAS will assess whether your figures meet our technical requirements by comparing each figure against our figure specifications.

Attachment Submitted filename: PONE-D-25-28523_R2 2nd Correction with Comments.pdf

# Author comment: Author response to Decision Letter 3

**DOI:** 10.1371/journal.pone.0358063.r006

19 Aug 2026

Dear Editors,

We sincerely thank the Academic Editor and Reviewer #6 for their detailed review of the manuscript and for the positive assessment of our previous revisions.

We have made the following changes:

- In line with the recommendation, we have revised the title of the manuscript to: “Learning from Pandemics: Feature Selection and Clustering for Robust Digital Disease Nowcasting.”

- We carefully reviewed the manuscript and addressed all the corrections identified by Reviewer #6, including formatting, mathematical notation, spacing, and typographical symbols.

- For the technical terms representing literal parameter or option values (e.g., hl="en-us", related queries, "auto", and "sqrt"), we have formatted these as code to distinguish them from ordinary quoted text while preserving their original syntax.

- We have also revised the bibliography to:

a) add one relevant and very recent paper (48. Costa-Gomes B, Tolmachev P, Taysom E, Sounderajah V, Richardson H, Schoenegger P, et al. Public use of a generalist LLM chatbot for health queries. Nature Health. 2026:1-8.);

b) update references to preprints that have since been published, replacing the preprint citations with the corresponding final journal citations;

c) correct the access dates where appropriate;

d) confirm that the reference formatting adheres to PLoS guidelines.

A marked-up version comparing the previous and revised manuscripts is provided alongside the clean revised manuscript.

Regarding the reviewer’s comment about “GOogle” in AutoRegression with GOogle search data, we have retained the capitalization, as this is intentional and corresponds to the original expansion of the ARGO acronym.

Again, we thank both the Editor and the Reviewers for their comments and hope that the revised manuscript now addresses all remaining points.

On behalf of all authors,

Joana Gonçalves-Sá

# Editor report: Decision Letter 3

**DOI:** 10.1371/journal.pone.0358063.r007

**Authors:** Kyungwon Kim

27 Aug 2026

Learning from Pandemics: Feature Selection and Clustering for Robust Digital Disease Nowcasting

PONE-D-25-28523R3

Dear Dr. Gonçalves-Sá,

We’re pleased to inform you that your manuscript has been judged scientifically suitable for publication and will be formally accepted for publication once it meets all outstanding technical requirements.

Within one week, you’ll receive an e-mail detailing the required amendments. When these have been addressed, you’ll receive a formal acceptance letter and your manuscript will be scheduled for publication.

An invoice will be generated when your article is formally accepted. Please note, if your institution has a publishing partnership with PLOS and your article meets the relevant criteria, all or part of your publication costs will be covered. Please make sure your user information is up-to-date by logging into Editorial Manager at Editorial Manager® and clicking the ‘Update My Information' link at the top of the page. For questions related to billing, please contact billing support.

If your institution or institutions have a press office, please notify them about your upcoming paper to help maximize its impact. If they’ll be preparing press materials, please inform our press team as soon as possible -- no later than 48 hours after receiving the formal acceptance. Your manuscript will remain under strict press embargo until 2 pm Eastern Time on the date of publication. For more information, please contact onepress@plos.org.

Kind regards,

Kyungwon Kim

Academic Editor

PLOS One

Additional Editor Comments (optional):

Dear Authors,

Congratulations on the acceptance of your manuscript! I would like to commend you on your diligent efforts throughout the peer-review process. You have successfully addressed the final minor typographical and formatting corrections, and your updated title excellently reflects the comprehensive scope of your methodology.

Thank you for choosing to publish your valuable research with PLOS ONE. I wish you continued success in your future academic endeavors.

Reviewers' comments:

# Editor report: Acceptance letter

**DOI:** 10.1371/journal.pone.0358063.r008

**Authors:** Kyungwon Kim

PONE-D-25-28523R3

PLOS One

Dear Dr. Gonçalves-Sá,

I'm pleased to inform you that your manuscript has been deemed suitable for publication in PLOS One. Congratulations! Your manuscript is now being handed over to our production team.

At this stage, our production department will prepare your paper for publication. This includes ensuring the following:

* All references, tables, and figures are properly cited

* All relevant supporting information is included in the manuscript submission,

* There are no issues that prevent the paper from being properly typeset

You will receive further instructions from the production team, including instructions on how to review your proof when it is ready. Please keep in mind that we are working through a large volume of accepted articles, so please give us a few days to review your paper and let you know the next and final steps.

Lastly, if your institution or institutions have a press office, please let them know about your upcoming paper now to help maximize its impact. If they'll be preparing press materials, please inform our press team within the next 48 hours. Your manuscript will remain under strict press embargo until 2 pm Eastern Time on the date of publication. For more information, please contact onepress@plos.org.

You will receive an invoice from PLOS for your publication fee after your manuscript has reached the completed accept phase. If you receive an email requesting payment before acceptance or for any other service, this may be a phishing scheme. Learn how to identify phishing emails and protect your accounts at https://explore.plos.org/phishing.

If we can help with anything else, please email us at customercare@plos.org.

Thank you for submitting your work to PLOS One and supporting open access.

Kind regards,

PLOS One Editorial Office Staff

on behalf of

Professor Kyungwon Kim

Academic Editor

PLOS One
